# The presence of broadly neutralizing anti-SARS-CoV-2 RBD antibodies elicited by primary series and booster dose of COVID-19 vaccine

**DOI:** 10.1371/journal.ppat.1012246

**Published:** 2024-06-10

**Authors:** Xiaorui Chen, Arpita Mohapatra, Hong Thuy Vy Nguyen, Lisa Schimanski, Tiong Kit Tan, Pramila Rijal, Cheng-Pin Chen, Shu-Hsing Cheng, Wen-Hsin Lee, Yu-Chi Chou, Alain R. Townsend, Che Ma, Kuan-Ying A. Huang

**Affiliations:** 1 Genomics Research Center, Academia Sinica, Taipei, Taiwan; 2 Chemical Biology and Molecular Biophysics program, Taiwan International Graduate Program, Academia Sinica, Taipei, Taiwan; 3 Institute of Biochemical Sciences, National Taiwan University, Taipei, Taiwan; 4 MRC Human Immunology Unit, Weatherall Institute of Molecular Medicine, University of Oxford, John Radcliffe Hospital, Oxford, United Kingdom; 5 Chinese Academy of Medical Science (CAMS) Oxford Institute (COI), University of Oxford, Oxford, United Kingdom; 6 Department of Infectious Diseases, Taoyuan General Hospital, Ministry of Health and Welfare, Taoyuan, and Institute of Clinical Medicine, National Yang Ming Chiao Tung University, Taipei, Taiwan; 7 Department of Infectious Diseases, Taoyuan General Hospital, Ministry of Health and Welfare, Taoyuan, and School of Public Health, Taipei Medical University, Taipei, Taiwan; 8 Biomedical Translation Research Center, Academia Sinica, Taipei, Taiwan; 9 Graduate Institute of Immunology and Department of Pediatrics, National Taiwan University Hospital, College of Medicine, National Taiwan University, Taipei, Taiwan; National Institute for Communicable Diseases, SOUTH AFRICA

## Abstract

Antibody-mediated immunity plays a key role in protection against SARS-CoV-2. We characterized B-cell-derived anti-SARS-CoV-2 RBD antibody repertoires from vaccinated and infected individuals and elucidate the mechanism of action of broadly neutralizing antibodies and dissect antibodies at the epitope level. The breadth and clonality of anti-RBD B cell response varies among individuals. The majority of neutralizing antibody clones lose or exhibit reduced activities against Beta, Delta, and Omicron variants. Nevertheless, a portion of anti-RBD antibody clones that develops after a primary series or booster dose of COVID-19 vaccination exhibit broad neutralization against emerging Omicron BA.2, BA.4, BA.5, BQ.1.1, XBB.1.5 and XBB.1.16 variants. These broadly neutralizing antibodies share genetic features including a conserved usage of the IGHV3-53 and 3–9 genes and recognize three clustered epitopes of the RBD, including epitopes that partially overlap the classically defined set identified early in the pandemic. The Fab-RBD crystal and Fab-Spike complex structures corroborate the epitope grouping of antibodies and reveal the detailed binding mode of broadly neutralizing antibodies. Structure-guided mutagenesis improves binding and neutralization potency of antibody with Omicron variants via a single amino-substitution. Together, these results provide an immunological basis for partial protection against severe COVID-19 by the ancestral strain-based vaccine and indicate guidance for next generation monoclonal antibody development and vaccine design.

## Author summary

Antibody-mediated immunity serves as a key element of protection against SARS-CoV-2. B-cell-derived anti-SARS-CoV-2 RBD antibody repertoires from vaccinated and infected individuals were characterized and genetic and molecular features of antibody breadth and potency against viruses were elucidated. A portion of anti-RBD antibody clones that develops after a primary series or booster dose of ancestral strain-based COVID-19 vaccination exhibit broad neutralization against Omicron variants. For example, antibody JL-8C recognizes a distinct epitope (Class 3b epitope) below the tip of RBD opposite to the epitope targeted by FD-11A or S309, and potently neutralizes Omicron variants BA.4/5, XBB.1.5 and XBB.1.16. Our findings suggest that with repeated antigen exposures the antibody repertoire had broadened with epitopes partially overlapping the classically defined set identified early in the pandemic.

## Introduction

SARS-CoV-2 emerged in 2019, spread rapidly around the world and now causes endemic respiratory illness. As immune protection correlates with neutralizing antibody responses, the breadth and potency of antibodies in humans have been constantly challenged by the emergence of new SARS-CoV-2 variants [1–4]. Since late 2021 when the first Omicron strains emerged, until the currently circulating XBB strains that arose at the end of 2022 to the beginning of 2023, the SARS-CoV-2 variants have been exhibiting a notable increase in virus fitness, with enhanced ACE2 binding, transmission and immune evasion [5,6].

The spike glycoprotein is the major target for neutralizing antibodies, which makes it a key candidate for both vaccine development and immunotherapy [7]. B cell responses in COVID-19 patients have been detected concomitantly with follicular helper T cell responses from week 1 after illness onset [8,9]. In the T cell-dependent humoral response, the germinal center reaction promotes competitive selection and clonal expansion of high-affinity antigen-specific B cells, leading to the establishment of antibody immunity to viral infections in humans. The mapping of spike-specific antibody responses, from polyclonal serology to single cell sequencing, in convalescent patients who had acute SARS-CoV-2 infection and vaccinated persons, has shown that neutralizing antibodies can target many different epitopes [10–13]. The most potent antibodies tend to target the receptor-binding domain (RBD) of spike and interfere with the binding of RBD with the human ACE2 receptor [14].

The accumulation of mutations in the RBD of spike, i.e., N501Y in Alpha variant, E484K and K417N in Beta variant, L452R and T478K in Delta variant, not only affect the virus phenotype but also facilitate its immune evasion [15]. These residue substitutions are also found in the Omicron (B.1.1.529) variant and its subvariants (including BA.2, BA.4, BA.5, BA.2.12.1, BQ.1.1, XBB.1.5, XBB.1.16). These variants contain other changes probably due to antibody-driven selection pressure and have become more resistant to neutralizing antibodies elicited by COVID-19 vaccines [15,16]. In 2022, BA.4 and BA.5 emerged and rapidly replaced BA.2, carrying several different mutations such as F486V and L452R [17]. More recently, the subvariant BQ.1 and BQ.1.1, a descendant of BA.5, harbors five convergent substitutions (R346T, K444T, L452R, N460K, and F486V) and has been classified as variants under monitoring by the World Health Organization [18]. The XBB.1.5 and XBB.1.16 subvariants, descendants of BA.2, carried new mutations (V445P, T478K or T478R, F486P, and F490S) and gradually dominated the worldwide SARS-CoV-2 infection in early 2023, with rapid spreading and yet milder symptoms [5,6,19]. Neutralization escape by the Omicron variant has also been observed following vaccinations, regardless of the vaccine type and platform [20]. However, booster doses, especially using mRNA vaccines, may enhance neutralization capacity against Omicron [20–23]. Recovered patients also exhibit reduced neutralizing titers against multiple Omicron subvariants, with BA.4, BA.5 and BQ.1.1 detected globally [18,24].

In this study, we analyzed anti-RBD B cell-derived antibody response of naïve persons after primary series and booster dose of COVID-19 vaccine as well as recovered COVID-19 patients, and found that vaccination with the ancestral spike elicited a subset of broadly neutralizing antibodies against Omicron subvariants after repeated exposures. Distinct genetic and structural features are involved in the development of this antibody response. The structural basis of immune evasion by later Omicron strains was studied among the broad neutralizing VH3-53-family antibodies. Structure-guided mutagenesis that modifies the CDR loops was also shown to be a useful tool to subvert the impact of certain antigenic mutations, highlighting the potential for a strategy to delineate the SARS-CoV-2 antibody response and facilitate further therapeutic and vaccine development.

## Results

### Anti-RBD B cell response and derived antibody repertoires

Twelve adult donors after the primary series of COVID-19 vaccine (two doses), three adult donors after booster dose of COVID-19 vaccine (three doses) and six COVID-19 patients (unvaccinated) were enrolled in the study (**S1 Table**). Peripheral B cells were stained with labeled RBD proteins and analyzed. Each individual developed a distinct anti-RBD antibody-secreting B cell response to vaccination or natural infection and the frequencies were 0.17 ± 0.05, 0.30 ± 0.10 and 0.06 ± 0.02% of circulating B cells among primary series vaccinated, boosted and infected donors, respectively (ANOVA, P = 0.0021) (**S1 Fig**).

**Fig 1:**
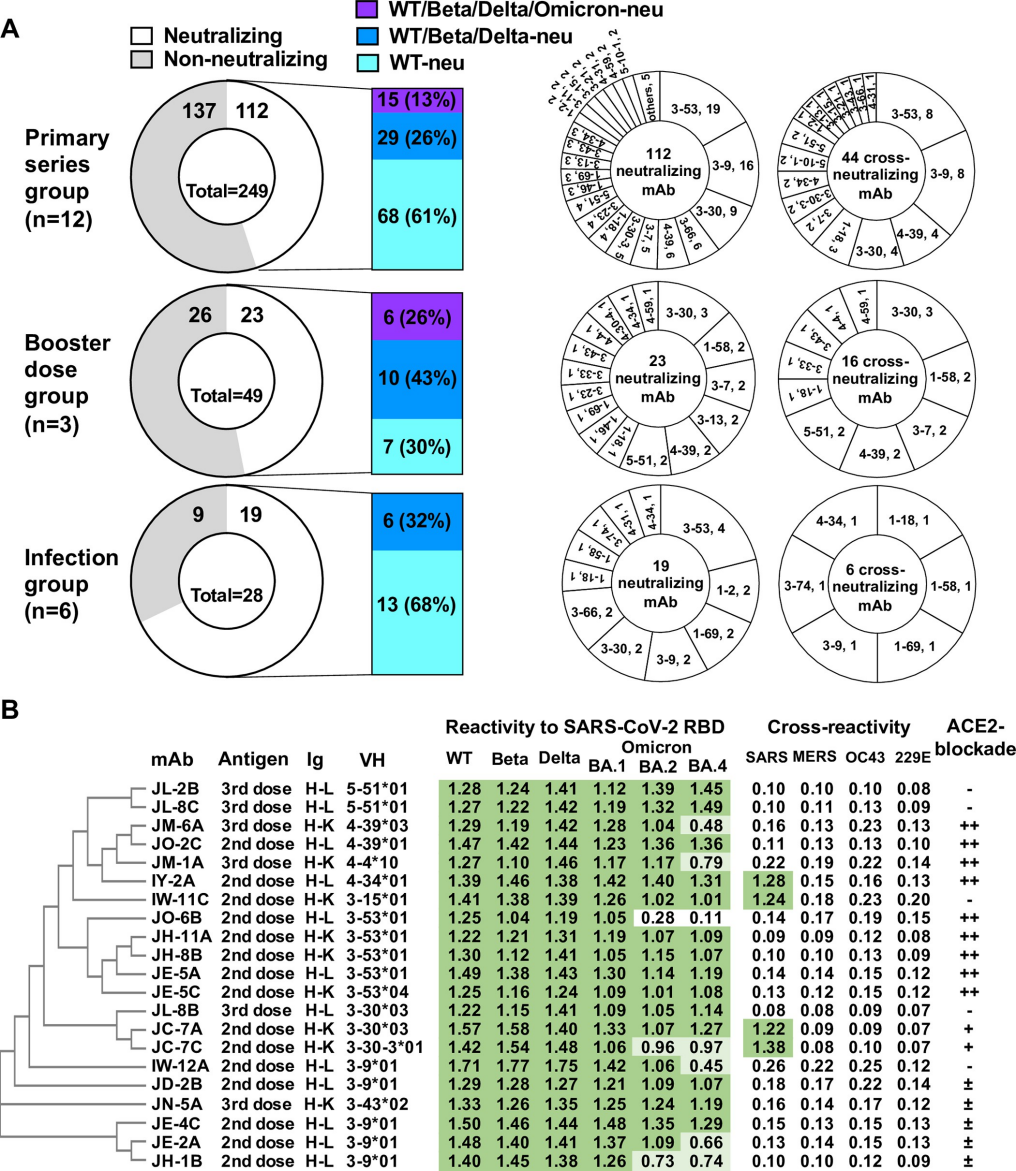
Anti-RBD B cell-derived antibody clones from vaccinated and infected donors. **(A)** Functional and genetic characterizations of anti-RBD antibody clones from 12 donors after receiving primary series of COVID-19 vaccine, 3 donors after booster dose of COVID-19 vaccine and 6 donors after natural infection of SARS-CoV-2. **(B)** Functional and antigenic breadth of broadly neutralizing anti-RBD antibody clones. The binding activities of antibody (2 μg/ml) with SARS-CoV-2 RBDs and other coronaviruses were measured in the ELISA. The ACE2-blocking activity of antibody was measured and compared with ACE2-Fc. The mixture of biotinylated RBD with antibodies (10 μg/ml) was incubated with MDCK-SIAT1 cells stably expressing human ACE2. WT, wild-type SARS-CoV-2 (Wuhan-Hu-1); Beta, Beta variant of SARS-CoV-2; Delta, Delta variant of SARS-CoV-2; Omicron, Omicron variant of SARS-CoV-2; SARS1, SARS-CoV; MERS, Middle East respiratory syndrome–related coronavirus; OC43, human coronavirus OC43; 229E, human coronavirus 229E; Neu, neutralizing; mAb, monoclonal antibody; Ig, immunoglobulin heavy chain-light chain; K, kappa chain; L, lambda chain; VH, heavy chain variable region gene usage. -, no competition; ±, 10–49% competition; +, 50–74% competition; ++, >75% competition.

Single positive cells were sorted and subjected to RT-PCR to generate the monoclonal antibody (mAb) repertoire. A total of 249, 49 and 28 anti-RBD IgG mAbs were produced from primary series vaccinated, boosted and infected donors, respectively (**Fig 1A**). We screened these antibodies for neutralization against pseudotyped viruses of wild type (Wuhan-Hu-1, WT), Beta, Delta and Omicron BA.1. There were 112 (45%), 23 (47%) and 19 (68%) of anti-RBD antibodies from primary series vaccinated, boosted donors and patients, respectively, which neutralized the WT virus (Chi-square, P = 0.0711) (**Fig 1A and S2 Table**). A larger portion of the WT-neutralizing antibodies from boosted donors cross-neutralized Beta and Delta viruses (70%), compared to those in primary series vaccinated donors (39%) and in patients (32%) (Chi-square, P = 0.0160). Nevertheless, the majority of the above cross-neutralizing antibodies failed to neutralize Omicron BA.1 virus (**Fig 1A and S2 Table**). Those WT-neutralizing antibodies that failed to react against Beta, Delta or Omicron BA.1 exhibited reduced or lost binding activities with corresponding RBDs in the ELISA (**S2 Table**).

Among primary series vaccinated donors, two received both doses of adenovirus-vectored vaccine (ChAdOx1) [25], two received heterologous adenovirus-vectored (ChAdOx1) and subunit vaccines (MVC-COV1901) [26], and eight received both doses of mRNA vaccine (mRNA-1273) [27] (**S1 Table**). In the latter two subgroups, higher rates of WT-neutralizing (25% for homogenous adenovirus-vectored subgroup, 36% for heterologous subgroup, 47% for mRNA subgroup, Chi-square, P = 0.3040) and cross-neutralizing (13% for homogenous adenovirus-vectored subgroup, 28% for heterologous subgroup, 17% for mRNA subgroup, Chi-square, P = 0.3447) antibodies were found in the anti-RBD B cell repertoire, but the difference did not reach statistical significance (**S2 Table and S2 Fig**).

The expansion of the anti-RBD B cell response to vaccination or infection involved diverse VH-gene usages with preferred families in WT-neutralizing and cross-neutralizing antibodies (**S3 Fig**). VH3-53, 3–9, 3–30 and 4–39 were frequently used in neutralizing and cross-neutralizing antibodies from primary series vaccinated donors (**Fig 1A)**. Neutralizing antibodies elicited after the booster dose were encoded with a broad range of VH genes, such as VH3-30, 1–58, 3–7, 4–39 and 5–51, accounting for 48% and 69% of WT- and cross-neutralizing antibodies (**Fig 1A and S2, S3 and S4 Tables**). In addition, unique paired VH/VL gene clonal sets were identified in the WT- and cross-neutralizing antibodies (**S3 Fig**). Neutralizing antibodies in the largest set (n = 11) are encoded by VH3-53 and Vκ3–20, and Vκ1–33 or Vκ1–9 gene usage was also observed in the light chain. This distinct set was mainly observed in neutralizing antibodies from primary series vaccinated donors, which also represented the most dominant group of anti-RBD antibodies [28–31]. By contrast, VH4-39/Vκ1-NL1 and VH5-51/Vλ1–44 antibodies represented two major sets among WT- and cross-neutralizing antibodies from boosted donors.

The nucleotide mutations in V genes of neutralizing antibody heavy and light chains from primary series vaccinated donors were 5.9 ± 0.4 and 2.2 ± 0.2, respectively, which were higher than those in antibodies cloned from SARS-CoV-2-infected individuals (VH 1.9 ± 0.7 and VL 1.0 ± 0.3), and lower than antibodies derived from boosted donors with mRNA COVID-19 vaccine (VH 13.3 ± 1.1 and VL 6.5 ± 0.6; ANOVA, P < 0.0001 for both VH and VL comparisons) (**S4A Fig and S3 Table and S1 Data**). Similar differences in the amino acid substitutions of neutralizing antibody heavy and light chains were also found among primary series vaccinated, boosted and infected donors (**S4B Fig, S1 Data**). These findings suggested that RBD-activated B-cell clones could expand and undergo immunoglobulin gene hypermutation in the germinal center response following antigen re-exposure. Nevertheless, heavy chain CDR3 length was indistinguishable among neutralizing antibodies from different groups of donors (ANOVA, P = 0.9742) (**S3 Table**)

A subset of cross-neutralizing antibodies (15 from primary series vaccinated donors, 6 from boosted donors) broadly neutralized Omicron BA.1 variant, but no cross-reactive antibodies were found in recovered patients (**Fig 1A and 1B)**. Nine of these broadly neutralizing antibodies strongly competed with ACE2 receptor for binding to the RBD, and VH3-53 and VH4-39 genes dominated these ACE2-blocking antibodies (**Fig 1B**). Further tests revealed that seven BA.1-neutralizing antibodies exhibited reduced binding activities with Omicron BA.2 and BA.4 RBDs. The majority of these broadly neutralizing antibodies (17 of 21, 81%) did not cross-react with other coronaviruses in the ELISA (**Fig 1B**).

### Broadly neutralizing anti-RBD antibodies

To understand the epitope region recognized by representative broadly neutralizing antibodies isolated from COVID-19-vaccinated donors, we performed a cross-competition assay with a panel of well-characterized antibodies. By adding a biotinylated version of representative mAb together with excess of each of the other mAbs individually into ELISA plates pre-coated with SARS-CoV-2 RBD, we detected cross-competition of mAbs. As previously described, human mAbs FI-3A (Class 1) [10,32], C121 (Class 2) [10,33], FD-11A (Class 3) [10,32] and EY-6A (Class 4) [10,34,35] that were derived from COVID-19 convalescent patients were included as controls and their structural footprints on the RBD had been characterized [10,32–35]. Also included was the protease domain (residues 18–615) of ACE2 linked to the Fc region of human IgG1 (ACE2-Fc dimer) [32–35].

**Fig 2:**
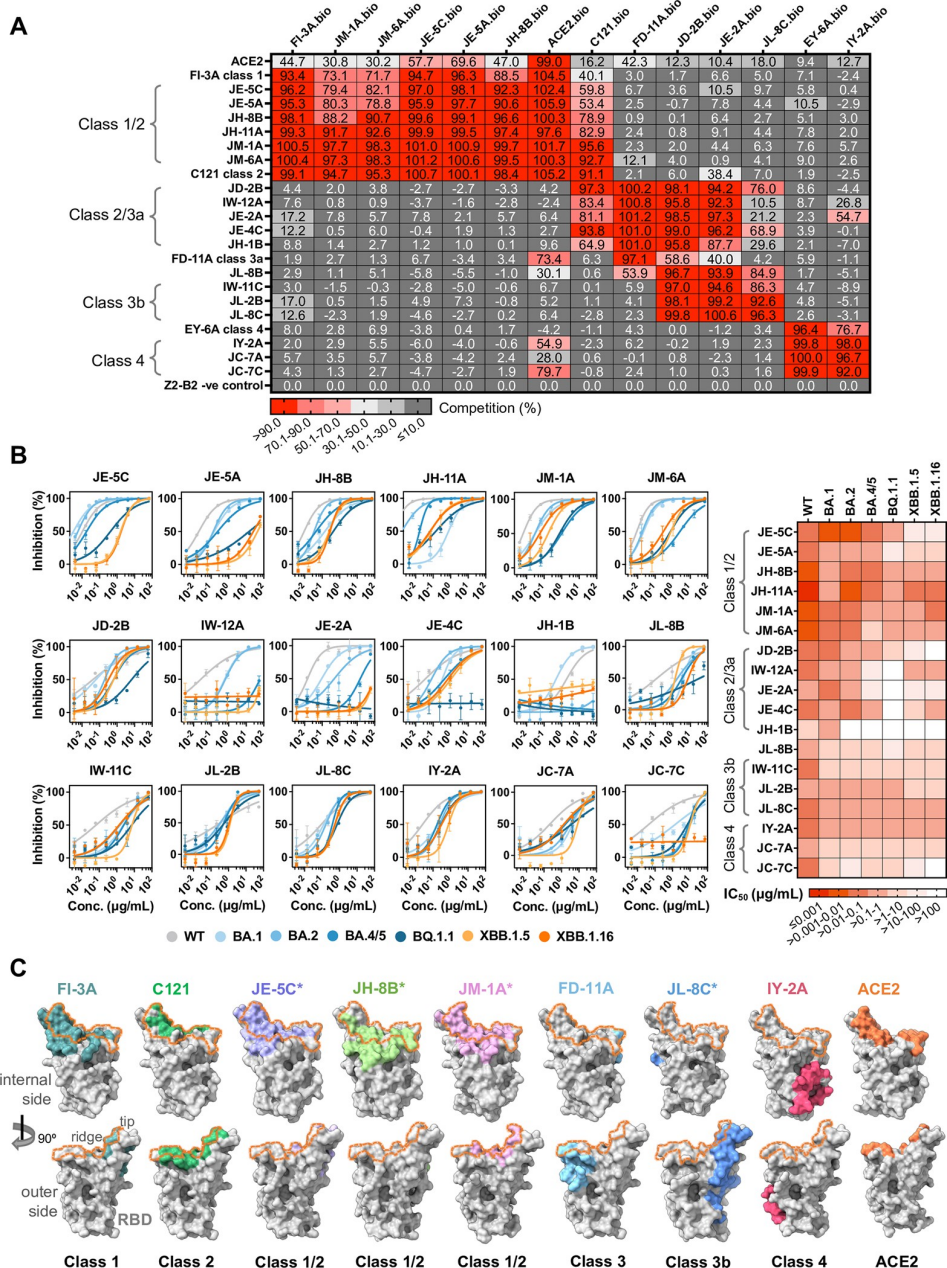
Characterization of broadly neutralizing anti-RBD antibody clones. **(A)** Epitope mapping of anti-RBD antibody clones based on the cross-competition assay. FI-3A (Class 1) [10,32], C121 (Class 2) [10,33], FD-11A (Class 3) [10,32], EY-6A (Class 4) [10,34,35] and IY-2A (Class 4) [35] were included as controls. **(B)** Neutralization activity of antibodies with wild type (WT, Wuhan-Hu-1), Omicron BA.1, BA.2, BA.4/5, BQ.1.1, XBB.1.5, XBB.1.16 variants, shown as percentage of inhibition in curves colored in grey (WT), blue gradients (from BA.1 to BQ.1.1), light orange (XBB.1.5) and dark orange (XBB.1.16). Data are mean ± standard deviation from technical duplicates, and curves are fit by nonlinear regression for half-maximal inhibitory concentrations (IC_50_ values), as summarized in a heat map on the right or in S5 Table. **(C)** Footprints of representative anti-RBD antibodies from each cluster are revealed by crystal or cryo-EM structures, with RBD shown as grey surface in both back (upper row) and front (lower row) views, and each footprint colored accordingly, overlapped with footprint of ACE2 (dark orange line). Footprints of previously reported antibodies from Class 1 (FI-3A, PDB 7PQY) [32], Class 2 (C121, PDB 7K8X) [33], Class 3 (FD-11A, PDB 7PQZ) [32] and Class 4 (IY-2A, PDB 7YCN) [35], as well as that of ACE2 (orange, PDB 6M0J) [14], were shown for comparison. The internal and outer sides of RBD, the RBD tip and ridge are labeled. Footprints refined by relative surface accessibility change threshold of 4%. Asterisks highlight structures newly reported in this study.

We identified three major clusters of competing mAbs that share binding properties with the four well defined classes identified early in the pandemic [10–12] (**Fig 2A**). The clusters defined relatively broad epitopic regions, as the footprints of two mAbs within one cluster might overlap with each other, but both might overlap with the footprint of a third antibody.

The Class 1/2 cluster mAbs, JE-5C, JE-5A, JH-8B, JH-11A, JM-1A and JM-6A, competed with both FI-3A (Class 1) [10,32] and C121 (Class 2) [10,33], although asymmetric competition could occur. For example, JM-1A and JM-6A blocked binding of C121 (Class 2) as well as FI-3A (Class 1). Class 1/2 cluster mAbs strongly competed with ACE2 for binding to RBD in the assay, as FI-3A and C121 did.

In our previous collection we defined antibodies (represented by FD-11A) that competed with S309, a typical Class 3 antibody [10,32]^.^ These Class 3 antibodies were distinct from Class 2, and they did not compete with Class 2 antibodies in either direction (represented by FD-11A and C121, **Fig 2A**). A single antibody JL-8B behaved as a typical Class 3 antibody. By contrast, in the present set we observed a distinct pattern represented by five antibodies (JD-2B, IW-12A, JE-2A, JE-4C, and JH-1B) that competed with both C121 (Class 2) and FD-11A (Class 3) for binding to RBD that we refer to as Class 2/3a.

A second cluster was defined by three antibodies (IW-11C, JL-2B and JL-8C) that failed to compete with classically defined Class 1 (FI-3A), Class 2 (C121), Class 3 (FD-11A) or Class 4 (EY-6A), but did compete with a subgroup of the defined Class 2/3a mAbs. We refer to these as Class 3b. The majority of Class 2/3a and 3b cluster mAbs did not compete for ACE2 binding to RBD.

Finally we observed a set of three Class 4 antibodies (IY-2A, JC-7A and JC-7C) that competed with the structurally defined mAb EY-6A [34], and blocked ACE2 binding to varying extents (**Fig 2A**). IY-2A represents a Class 4 antibody with a unique mode of binding and has been characterized [35].

These broadly neutralizing antibodies were further tested for neutralization in the pseudovirus assay against the later emerged SARS-CoV-2 Omicron variants [17–19]. The antibodies showed varied levels of neutralizing potency against Omicron variants BA.4/5, with three Class 1/2 mAbs, JE-5C, JH-11A, and JH-8B, being most potent with half-maximal inhibitory concentrations (IC_50_ values) less than 0.1 μg/ml. They also retained neutralization against Omicron BQ.1.1, with IC_50_ values of 0.43, 0.14 and 0.43 μg/ml, respectively. But JH-11A was the only one with high potency extended against XBB.1.5 and XBB.1.16 (IC_50_ less than 0.1 μg/ml), while JH-8B had slightly reduced (IC_50_ around 0.2 μg/ml) and JE-5C had much reduced activities (IC_50_ over 1 μg/ml) (**Fig 2B and S1 Data**). One exception was Class 1/2 mAb JE-5A which exhibited a dramatic reduction in neutralization after BQ.1.1 (IC_50_ over 10 μg/ml).

The Class 2/3 mAbs, including JD-2B, JE-4C, JL-2B, and JL-8C, neutralized BA.4/5 with IC_50_ values of less than 1.0 μg/ml and retained such IC_50_ values against XBB.1.5 and XBB.1.16 (**Fig 2B and S1 Data**). Class 2/3a mAbs IW-12A and JE-2A neutralized BA.1 and BA.2 but had greatly reduced or lost activities against BA.4/5 and BQ.1.1 viruses. Another class 2/3 mAb JH-1B failed to neutralize all the subvariants after BA.2.

In contrast, the Class 4 mAb IY-2A [35] neutralized all the strains, whereas other Class 4 mAbs JC-7A and JC-7C fared worse (all IC_50_ values above 1.0 μg/ml), probably because their footprints were affected by hotspot mutations in the later Omicron subvariants [35].

Footprints of the above-mentioned mAbs were determined by crystal and cryo-EM (electron microscopy) structures, with representatives for each class except the Class 2/3a set (**Fig 2C**). JE-5C, JH-8B and JM-1A belong to Class 1/2 and their footprints locate around the ridge of RBD, heavily overlapped with that of ACE2. Both JE-5C and JH-8B carry the characteristic VH3-53 gene cluster and mainly recognize the internal side of the RBD ridge, with the internal side of the RBD defined as the buried surface when RBD is in the all-down conformation [7]. JE-5C revealed a highly similar footprint with the classical Class 1 antibody, and JH-8B revealed a slightly shifted one with extension on the internal side but shrinkage on the outer side (**Fig 2C**). This area is featured by epitopic regions of 415–421, 453–458, 486–489, and 501–505 (**S5 Fig**), which was suggested to avoid most of the hotspot mutations from variants of concern [15,30,36]. On the other hand, JM-1A is encoded with a less frequently used gene, VH4-4, and extends its footprint from the internal side over the ridge to the outer side of RBD (**Fig 2C and S3 Table**).

This outer side can be recognized by Class 3 antibodies, such as the previously reported C110 [10], S309 [10] and FD-11A [10,32], etc. Unlike these typical Class 3 antibodies, one of our Class 3b antibodies, JL-8C, recognizes a distinct epitope below the tip of RBD opposite to the epitope targeted by FD-11A [32] or S309 [10], with the footprint covering regions of 394–396, 464–472, and 481–484, which partially overlaps with the ACE2 binding site and is similar to the epitope of S2H97 [37] (**Fig 2C**). The footprints of other Class 3b antibodies in this study (IW-11C and JL-2B) are yet to be resolved.

The Class 4 antibodies are interacting with the farthest region away from the footprint of ACE2 and may partially compete with receptor binding by steric occlusion [38]. We have described a set of overlapping Class 4 antibodies [35] and include the footprint of IY-2A, the most broadly reactive, for comparison.

The binding and functional patterns of the Class 2/3a and Class 3b antibodies suggested that with repeated antigen exposures the antibody repertoire had broadened but their footprints may partially overlap the previously defined sets of neutralizing antibodies [10,32,33] (**Fig 2A–2C**).

### Detailed structural analysis of the VH3-53 antibodies

**Fig 3:**
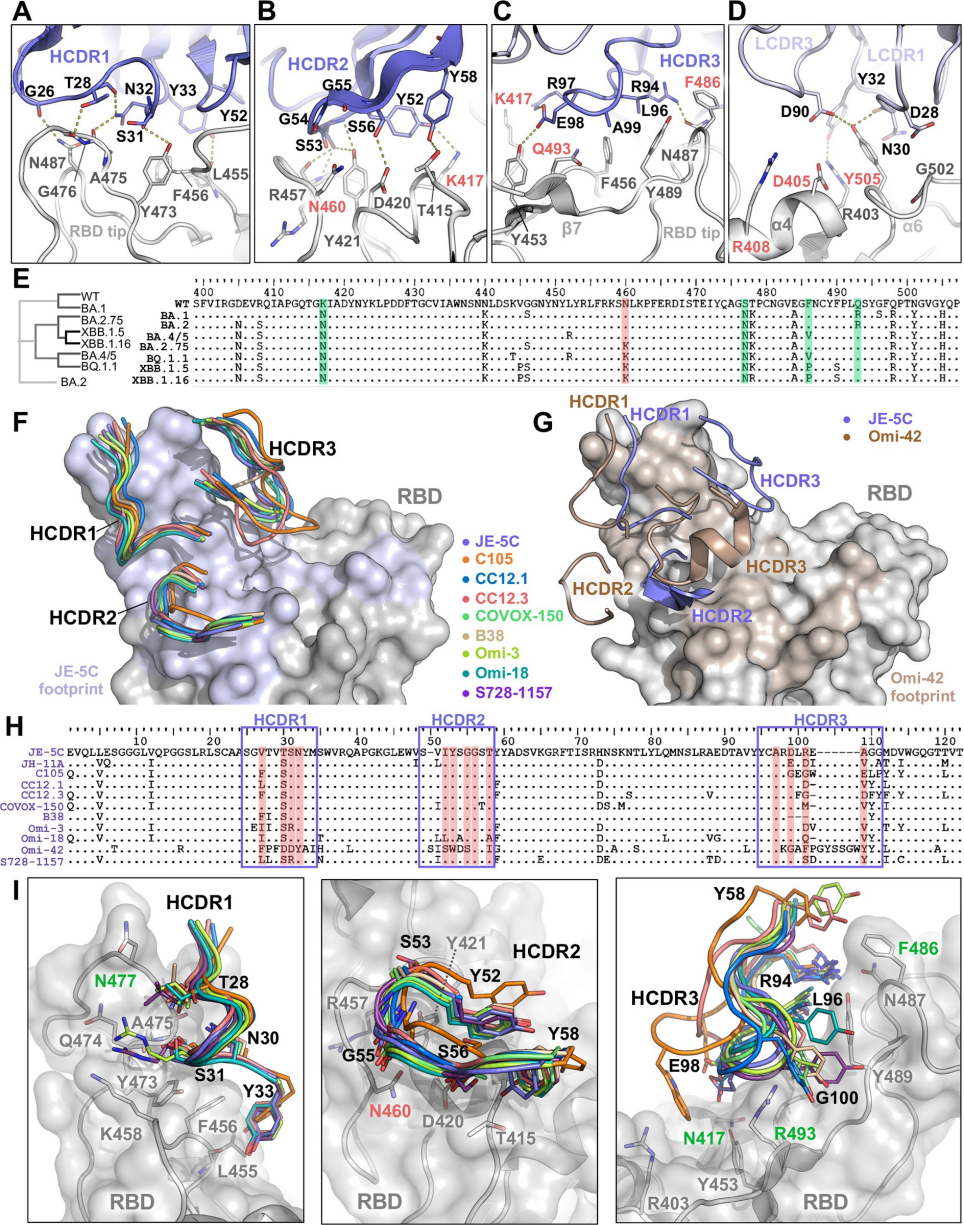
Detailed JE-5C/RBD interface and the structural similarity of VH3-53 family. **(A-D)** The detailed interface between RBD and each CDR loop of JE-5C as labeled. RBD: grey; heavy chain: dark purple; light chain: light purple. Key variable hotspot residues in the interface are labeled red. **(E)** The phylogenic tree and the sequence alignment of RBD (partial, 399–517) of WT and Omicron subvariants, highlighting K417N, S477K, F486V/P, Q493R in green and N460K in red. **(F)** The superimposition of HCDRs of eight mAbs [10,12,29,40–42] in the VH3-53 gene family, with CDR loops shown as ribbons, colored accordingly. RBD shown as grey surface and the footprint of JE-5C overlaid in light purple. **(G)** The superimposition of HCDRs of JE-5C and Omi-42 [29], with JE-5C in purple and Omi-42 in brown, and the footprint of Omi-42 overlaid in light brown. **(H)** Sequence alignment of VH domain of above antibodies in (F-G) and JH-11A, with HCDRs framed in purple boxes and key interacting residues highlighted in red. **(I)** Structural details of RBD recognition by VH3-53 antibodies [10,12,29,40–42], with residues that show consistency among different antibodies (black label) and their interacting residues on RBD (grey label) drawn as sticks. Key variable hotspot residues highlighted in green and red labels according to (E). PDB codes for involving antibody structures are 6XCM (C105), 8CWV (CC12.1), 6XC4 (CC12.3), 7ZF8 (COVOX-150), 7BZ5 (B38), 7ZF3 (Omi-3), 7ZFB (Omi-18), 8D0Z (S728-1157).

Crystal structure of the JE-5C-bound RBD complex revealed details of the interface between this VH3-53 antibody and RBD (**Fig 3A–3D**). The interaction is majorly contributed by its VH domain, of which the short HCDR1 loop (residue 26–33, Kabat numbering) clings along the RBD tip region, making hydrogen bonds with the highly conserved G476 and N487 (**Fig 3A**). An aromatic residue at the C-terminus of HCDR1, Y33, connects to the 453–456 loop by interacting with the carbonyl oxygen of L455 and packing with F456, which further extends in both sides to involve Y473 of RBD and Y52 on HCDR2 of JE-5C, forming a four-ring hydrophobic core (**Fig 3A**). An even more extensive interaction network is employed by HCDR2, involving almost all its residues (Y52, S53, G54, G55 and S56) to make hydrogen bonds with the side chains of T415, K417, D420 and Y421, as well as the backbone of R457 (**Fig 3B**). Such a binding mode pulls the entire VH domain close to the 457–460 and 415–421 loops of RBD, and the corresponding residues are mostly conserved in earlier lineages except for K417 which has been mutated to N since the emergence of Beta variant (**Fig 3E**). As for the Omicron strains, more mutations were identified within this footprint, such as N460K (BA.2.75, BQ.1, XBB.1.5 and XBB.1.16), L455F (XBB.1.5 and HK.3), L455S (JN.1), and F456L (EG.1, XBB.1.5.70, and HK.3) [39]. Particularly, the N460K mutation would place the Cε atom of K in an unfavorably close distance with both Oγ of S56 and carbonyl oxygen of G55 (**Fig 3B**). To avoid this, the whole HCDR2 loop may possibly be lifted, thus compromising other beneficial contacts and resulting in the reduced neutralization potency (> 20 times lower in IC_50_ from BA.5 to BQ.1.1) (**Fig 2B**). The further activity reduction of ~4 times against XBB.1.5 or XBB.1.16 could not be structurally explained but may be an indirect result of new mutations V445P and F490S.

The HCDR3 of JE-5C recognizes mainly the RBD ridge: R94 interacts with N487 in cooperation with G26 from HCDR1; the hydrophobic L96 and A99 are in ~3.5-5Å distance from the highly conserved Y489; R97 is close to K417 but may prefer to N417 in the later variants; and E98 makes a hydrogen bond with Y453 (**Fig 3C**). And the LCDRs contribute even less, contacting only R403 and Y505 (**Fig 3D**). Hotspot mutations such as D405N, R408S, F486V/P, Q493R, and Y505H do not appear to affect the binding of JE-5C (**Fig 3C–3D**). Although N30 and Y32 on LCDR1 interact with Y505 on α6 helix of RBD, the mutation Y505H may not disrupt the interaction but even facilitate a potential electrostatic bridge with D28 on LCDR1 (**Fig 3D**).

Superimposition of nine structures of VH3-53 antibody-RBD complexes including JE-5C reveals that all the HCDR loops adopt consistent binding modes (**Fig 3F**). These antibodies include C105 [10], CC12.1 [40], CC12.3 [40], COVOX-150 [12], B38 [41], Omi-3 [29], Omi-18 [29] and S728-1157 [42], but a more recent one, Omi-42 [29], approaches RBD with a shifted orientation (**Fig 3G**). This shift may be attributed to the long helical HCDR3 of Omi-42 which binds to the region that is otherwise recognized by HCDR2 for the rest of VH3-53 antibodies (**Fig 3F**), while the HCDR2 of Omi-42 is pushed outwards without significant contacts (**Fig 3G**). Sequence alignment reveals a high similarity among these VH3-53 antibodies especially in their HCDR2 loops, except for Omi-42 (**Fig 3H**). The consensus motif (Y_52_SGGSTY/F_58_) of HCDR2 was structurally well-defined [40,43], featured by two perpendicularly packed aromatic residues on each end of the hairpin-like loop, embracing five small-size hydrophilic residues (S/T/G) in the middle (**Fig 3B and 3I**). This motif may maintain its extensive interaction with RBD until N460 is mutated to K in the later strains (**Fig 2B**), while Omi-42 may not be sensitive to the N460K substitution (**Fig 3G and 3I**). Other key consistent residues include Y33 on HCDR1 that is responsible for the four-ring hydrophobic core (**Fig 3A and 3I**), and R/K94 on HCDR3 that is in contact with the highly conserved N487 (**Fig 3C and 3I**).

The structure-function correlation of VH3-53 antibodies is not limited to the VH domain, and the contribution of its light chain cannot be ignored. For example, the two antibodies identified in this study, JE-5C (VH3-53/Vκ1–33) and JH-8B (VH3-53/Vκ3–20) share 88% sequence identity in the VH but only 33% in the VL. JH-8B has a high potency against WT strain (IC_50_ 9 ng/ml) but a marked lower potency against BA.1 (IC_50_ 142 ng/ml) than JE-5C, and the activity was regained against BA.2 and later subvariants (**Fig 2B and S5 Table**). The difference between BA.1 and later subvariants lies in D405 and R408, as the D405N and R408S mutations may potentially alter hydrogen bonding profiles with nearby residues on LCDR3 (**S6A and S6B Fig**). Another interesting observation is the charge distribution of the paratope surface of these VH3-53 antibodies (**S7 Fig**). While only JE-5C and Omi-18 are rich in negative charges on the LCDRs, all the other antibodies exhibit a pattern that noncharged residues dominate, yet with a small negatively charged patch near the center, either contributed by HCDR3 or LCDR1 (**S7 Fig**). Whether this observation could inspire the structure-based antibody improvement awaits further investigation.

### Structure-based mutagenesis in improving antibody neutralization breadth

The detailed structural analysis as mentioned above provided a platform for designing structure-based mutagenesis to improve binding affinity and broaden cross-neutralization spectrum. For VH3-53 antibodies, mutating key HCDR2 residues was tried to bypass the impact of N460K mutation in BQ.1 and later subvariants (**Fig 3I**). However, none of the attempts (Y52E, Y52Q, S56G, or S56D) were successful, probably because this HCDR2 loop is so tightly engaged with RBD that any substitution tends to compromise its original binding strength (**S8 Fig**). This in turn highlights the significance of the conserved Y_52_SGGSTY/F_58_ motif of HCDR2 present in VH3-53 antibodies identified by different groups across the world [10,12,29,40–42]. Unless the entire binding mode is shifted as in the case of Omi-42 [29] (**Fig 3G**), a solution could hardly be found to overcome the impact of N460K mutation.

**Fig 4:**
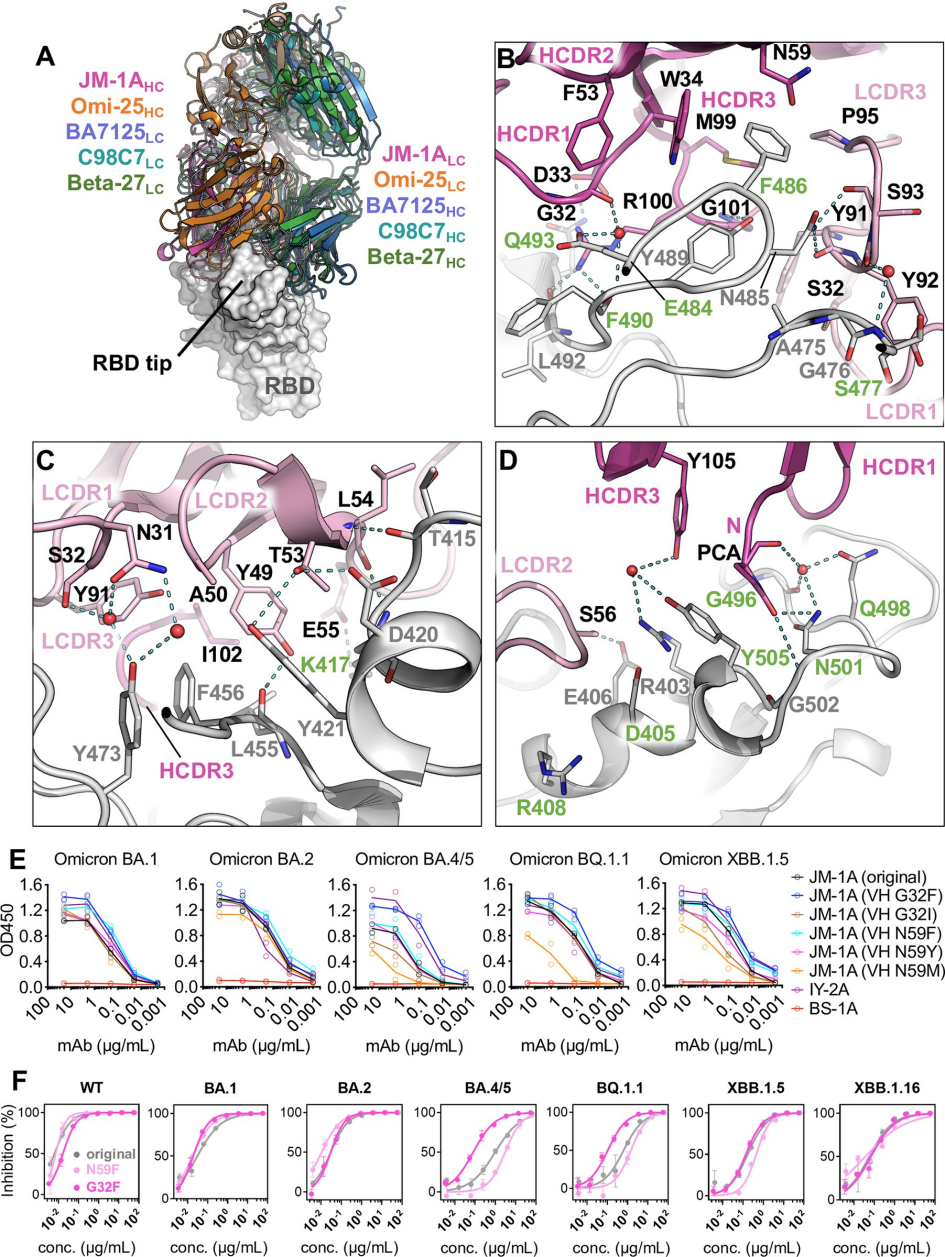
Detailed JM-1A/RBD interface and the functional improvement by mutagenesis. **(A)** Overall structure of JM-1A-Fab/RBD superimposed with antibodies adopting similar binding modes [17,29,44,45]. RBD is shown as grey surface, Fab in ribbons colored accordingly, and heavy and light chains are labeled. PDB codes for involving structures are 7ZFD (Omi-25) [29], 7XDA (BA7125) [44], 7SWO (C98C7) [45], and 8BH5 (Beta-27) [17] **(B-D)** The detailed interface between RBD and each CDR loop of JM-1A as labeled. RBD: grey; heavy chain: dark pink; light chain: light pink. Key variable hotspot residues in the interface are labeled green. **(E)** Binding activities of original and mutated JM-1A antibodies (G32F, G32I, N59F, N59Y or N59M of VH) with RBD in the ELISA, with each protein colored accordingly. Anti-RBD antibody IY-2A [35] and anti-influenza H3 antibody BS-1A were included as controls. (**F**) Neutralization against pseudoviruses of WT and Omicron subvariants by the original JM-1A (grey) and mutants (G32F, dark pink; N59F, light pink), shown as percentage of inhibition in curves. Data are mean ± standard deviation from technical duplicates, and curves are fit by nonlinear regression for half-maximal inhibitory concentrations (IC_50_ values), as summarized in S5 Table.

A more convincing case was found in JM-1A, another antibody identified in this study, with its RBD-complexed structure solved at 1.9 Å (**Fig 4**). As a Class 1/2 antibody, JM-1A shares a somewhat similar binding mode with several other antibodies which recognize both the inner and outer sides of the RBD ridge [17,29,44,45] (**Figs 2C and 4A**). But none of these antibodies could be well superimposed, and some have heavy and light chains swapped in position (**Fig 4A**). Compared to the most similar one Omi-25 [29] (**S9A Fig**), JM-1A has a longer HCDR1 and approaches RBD even further by tilting down its LCDR2 for more contacts and bringing the N-terminus of VH to the interface (**S9B and S9C Fig**). The extensive JM-1A/RBD interface involves the epitopic loops 403–408, 415–421 (α4 helix), 455–456, 473–477, 484–490, and 501–505 (α6 helix) that are majorly recognized by HCDR1, HCDR3, and LCDR2 (**Figs 4B–4D and S5**). Q1 at the VH N-terminus (cyclised as a pyroglutamate, PCA) stacks with the aromatic side chain of Y505 and forms hydrogen bond with N501 which is further connected with G496 and Q498 coordinated by a water molecule (**Fig 4D**). Moreover, the uncommonly employed LCDR2 is engaged with the 415–421 helix and 455–456 loop of RBD (**Fig 4C and 4D**). Mutations of Q493R, Q498R and N501Y in BA.1 variant may explain some of its decreased neutralization potency (**Fig 4B and 4D**), but its significant drop in the activity against BA.4/5 and BQ.1.1 is probably due to the only residue difference in that area, F486V, which may affect the surrounding interactions, including W34 (HCDR1), N59 (HCDR2), M99 (HCDR3), and P95 (LCDR3) (**Fig 4B**). A similar hydrophobic cluster was seen in other antibodies [17,29,44,45], although the local interaction is distinct in each structure (**S9D–S9G Fig**).

Substituting the only hydrophilic residue (N59 on HCDR2) to a hydrophobic one appeared to be a logical design for binding improvement in this F486-involved hydrophobic core (**S10A–S10C Fig**). However, neither N59M nor N59Y mutant enhanced its binding to RBD of Omicron variants, while the N59F mutant had slightly better affinities with BA.1 and BA.2 RBD (**Fig 4E**). The neutralization data also showed a marginal increase in the potency of N59F mutant against BA.1 (IC_50_: original, 30 ng/ml, N59F, 18 ng/ml) and BA.2 (IC_50_: original, 30 ng/ml, N59F, 11 ng/ml), but not the other subvariants (**Fig 4F and S5 Table and S1 Data**). Although this N59F mutant could stabilize interaction with F486 (WT and BA.1/2) or V486 (BA.4/5) or P486 (XBB1.5/1.16) of RBD, it may lose the original water-mediated hydrogen-bond network that shields the hydrophobic core (**S10A Fig**).

Another key residue in the paratope of JM-1A is G32 on HCDR1, and the G32F substitution was found to increase RBD binding in all the tested subvariants (**Fig 4E**). In the WT RBD, E484 interacts with the backbone carbonyl group of G32 via a water molecule (**Figs 4B and S10D**), but when it is mutated to A484 in Omicron variants, there opens a gap in this interface **(S10E Fig)**. The G32F mutant is predicted to fill this gap and also extend the original four-ring aromatic stacking to involve two more rings, F32 (JM-1A) and F490 (RBD), each spaced at ~3-4Å (**S10D–S10F Fig**), which could not be achieved by the G32I mutant (**Fig 4E**). Although the neutralization potency of JM-1A-G32F was slightly decreased against WT (IC_50_: original, 6 ng/ml, G32F, 16 ng/ml) due to the potential clash between E484 (RBD) and F32 (JM-1A) (**Fig 4F and S5 Table**), it showed substantially improved activity against BA.4/5 strain (IC_50_: original, 982 ng/ml, G32F, 80 ng/ml) through bypassing the influential F486V mutation (**Figs 4E, 4F, and S10F)**. Yet the neutralization against BA.1/2 was neither much enhanced (IC_50_: original, both 30 ng/ml; G32F, 19 and 30 ng/ml for BA.1 and BA.2, respectively) nor for XBB.1.5/16 (IC_50_: original, 169 and 57 ng/ml; G32F, 136 and 69 ng/ml for XBB.1.5 and XBB.1.16, respectively) (**Fig 4F)**. This is possibly explained as the Q493R mutation in BA.1/2 RBD may introduce a repulsion with R100 of JM-1A (**S10E Fig)**, while the F490S mutation in XBB.1.5/16 may lose the hydrophobic contact with G32F (**S10G Fig)**, both compromising the structural benefit from G32F mutation. Taken together all the factors that may interplay, JM-1A-G32F is the strongest one among all the mAbs reported in this study with subnanomolar potencies against all the Omicron subvariants tested so far.

## Discussion

Broadly neutralizing B-cell-derived antibodies were produced from individuals who received primary series and booster dose of SARS-CoV-2 vaccine, although these antibodies accounted for a small fraction of their RBD-specific B cells (6% for primary series vaccinated donors, 12% for boosted donors). None of the neutralizing antibodies isolated from infected donors neutralize Omicron variants in the study. Several studies have also found that the Omicron variants efficiently escape from neutralizing antibodies generated through previous infections with wild-type virus [4,15]. Previous findings revealed that the mRNA vaccine elicited robust neutralizing antibodies against wild-type, Beta, and Delta after primary series vaccination [36,46,47]. The booster dose could contribute to an expansion and evolution of RBD-specific memory B cells. Expanded clones of cells include pre-existing RBD-binding B cells that are elicited after the second dose of primary series vaccination and the development of new B cells as well [47]. This B cell response is accompanied with improved potency and breadth of neutralizing antibodies against the Omicron variant than antibodies detected after completion of the primary series [47,48]. Our results also showed that although the anti-RBD neutralizing antibody response is highly polyclonal and derives from a broad spectrum of different VH gene segments, clonal expansion and convergent antibody responses have been repeatedly observed in different individuals upon the primary series or booster vaccination (**Figs 1B and S3)**.

In the study, a significantly higher somatic mutation rate occurred in the anti-RBD neutralizing antibodies originated from boosted donors, followed by antibodies from primary series vaccinated donors and then by antibodies from patients. In SARS-CoV-2 infection, evidence showed that the neutralizing antibodies primarily originate from naïve B cells rather than pre-existing memory B cells [49]. The affinity of antibodies can be enhanced through somatic hypermutation and clonal evolution in the germinal center response to primary series or booster vaccination [47,48].

Clinical trials have demonstrated that COVID-19 vaccines based on the ancestral Wuhan-Hu-1 virus, particularly mRNA and subunit vaccines, are highly effective in combating laboratory-confirmed symptomatic infection and reducing the risk of severe illness [27,50,51]. While the efficacy of these vaccines may wane over time due to a decaying immune response, breakthrough infections can also occur due to the circulation of new variants, particularly in the setting of emergence of the Omicron variant and its subvariants [52]. However, when compared to unvaccinated populations, the COVID-19 vaccine still provides considerable protection against the risk of hospitalization and severe illness [53–57]. These results suggest that vaccinated individuals have more intensive humoral responses compared to those individuals who remained unvaccinated, which might be linked to partial protection against newly emerged Omicron and its subvariants. Recent studies also showed that cross-reactive anti-spike T cell response may develop upon primary series or booster vaccination, which could play a role in limiting the severity of COVID-19 [58,59]. These mechanisms are not mutually exclusive and may act together.

The subtle molecular interplay between neutralizing antibodies and mutated antigens impacts vaccine effectiveness. Structures of Fab/RBD complexes were determined in this study on representative mAbs, revealing how their neutralization potency could be much affected by a single residue substitution, by indirect allosteric effects, or by potential competition with ACE2. Such cases were described and discussed along with the structural data of VH3-53 antibody JE-5C and the engineered antibody JM-1A-G32F. The evolution of Omicron subvariants showed an overall increasing viral fitness, with two hotspot mutations of RBD highlighted in our structural data, N460K that contributes to fusogenicity and F486V that decreases ACE2 binding affinity but increases immune evasion [31,60,61]. The currently circulating XBB.1.5/16 has been shown to have a significantly higher resistance to humoral immunity than BA.5, BA.2.75 and BQ.1-like subvariants [5], while a reversed phenomenon was seen in some mAbs (JM-1A, JM-6A, IW-12A and JE-4C) in that they exhibited enhanced activities against XBB.1.5/1.16 compared to BA.4/5 or BQ.1.1 (**Fig 2B**). This may be attributed to differential competition between ACE2 and antibodies at the local interface where newly emerging mutations such as F486P and F490S enhance the binding of these antibodies, but not others like JE-5C or JD-2B.

There are some limitations in the study. Firstly, only anti-RBD antibody responses were analyzed. Protective antibodies that develop upon vaccination and infection and target other regions of SARS-CoV-2 spike protein were not determined. Previous studies have revealed the generation of cross-neutralizing antibodies that recognize the N-terminal domain and the S2 subunit of spike, although they were less potent against virus than anti-RBD antibodies and may lose activities with emerging variants [11,62]. Secondly, our study focuses on the characterization of the anti-RBD antibody-secreting B cell repertoire, which could not fully represent the scale of virus-specific B cell response. Next-generation sequencing approaches have been used to study B cell receptor heavy chain repertoires [63], which would enable characterization of the complete antigen-specific B cell landscape, at the single-cell level, after infection or vaccination of SARS-CoV-2. Thirdly, although the structural interface between mAb and RBD was analyzed in detail with sequence alignments and mutagenic studies in support, a full understanding of the molecular events that are taking place still requires further functional evidence. Finally, the classification of broadly neutralizing anti-RBD antibodies here was based mainly on competitive binding, and functional and structural information in comparison with previously defined classes of neutralizing antibodies [10,32–35]. Other efforts to more intensively classify neutralizing anti-RBD antibodies have also been reported [64,65].

In conclusion, we delineated the breadth and function of anti-RBD antibody responses between different groups of vaccinated and SARS-CoV-2-infected individuals. Representative structures were determined and provided part of the perspective on the mode of binding of these new antibodies, which may hopefully equip us with useful insights in preparation for future pandemics.

## Methods

### Ethics statement

The study protocol and informed consent were approved by the ethics committee at the National Taiwan University Hospital and the Taoyuan General Hospital, Ministry of Health and Welfare. Written informed consent was received from each participant prior to inclusion in the study. The study and all associated methods were carried out in accordance with the approved protocol, the Declaration of Helsinki and Good Clinical Practice guidelines.

COVID-19 patients who were diagnosed by positive SARS-CoV-2 rRT-PCR of nasopharyngeal swab samples were enrolled. Healthy adult donors who received COVID-19 vaccines were enrolled. Blood samples were collected from each donor.

### Isolation of monoclonal antibodies

Peripheral blood mononuclear cells were separated using Ficoll lymphocyte separation medium and were stained with fluorescent-labelled antibodies to cell surface markers with or without biotinylated beta RBD. Peripheral B cells were gated and isolated in chamber as single cells and single cells were used to produce human IgG mAbs as previously described [32,35]. Briefly, the variable region genes from each single cell were amplified in a reverse transcriptase polymerase chain reaction (QIAGEN, Germany) using a cocktail of sense primers specific for the leader region and antisense primers to the Cγ constant region for heavy chain and Cκ and Cλ for light chain. The RT-PCR products were amplified in separate polymerase chain reactions for the individual heavy and light chain gene families using nested primers to incorporate restriction sites at the ends of the variable gene. These variable genes were then cloned into expression vectors for the heavy and light chains. Plasmids were transfected into the HEK293T cell line for expression of recombinant full-length human IgG MAbs in serum-free transfection medium. A selected panel of MAbs were further expanded and purified.

To determine the individual gene segments employed by VDJ and VJ rearrangements and the number of nucleotide mutations and amino acid replacements, the variable domain sequences were aligned with germline gene segments using the international ImMunoGeneTics (IMGT) alignment tool (http://www.imgt.org/IMGT_vquest/input).

### ELISA

ELISA plates (Corning, USA) were coated with SARS-CoV-2 or other betacoronavirus RBD proteins (Sino Biological, China) at 4°C overnight. Plates were washed with phosphate-buffered saline containing 0.05% Tween-20 and blocked with 3% bovine serum albumin at room temperature for 1 hour on a shaker. Serial dilutions of mAb-containing cell culture supernatant or purified mAb were added and plates were incubated at 37°C for 1 hour. Plates were washed and incubated with horseradish peroxidase-conjugated rabbit anti-human IgG secondary antibody (Rockland Immunochemicals, USA). Plates were washed and developed with TMB substrate reagent (BD Biosciences, USA). Reactions were stopped with 0.5M hydrochloric acid and absorbances was measured at 450nm on a microplate reader. Non-transfected cell culture supernatant, anti-influenza H3 human IgG mAb BS-1A (in house) and convalescent serum were used as controls for each experiment. Reaction yielding an absorbance value above three times the mean absorbance of the negative control BS-1A (anti-influenza H3 human mAb) were considered positive.

### Neutralization assay

HEK293T cells stably expressing human ACE2 were seeded in a 96-well plate and incubated overnight. Monoclonal antibody of 5 μg per ml was prepared and mixed with pre-titrated pseudotyped lentiviruses expressing the wild-type Wuhan-Hu-1, Beta variant, Delta variant or Omicron variant spike proteins at 37°C for 1 h. They were then inoculated to the pre-seeded cells at 37°C, and incubated for further 16 h. The culture medium was then replaced with fresh Dulbecco’s Modified Eagle Medium supplemented with 1% fetal bovine serum and 100 U/mL Penicillin/Streptomycin. After a further incubation for 48 h, luciferase activity was measured using the Bright-Glo Luciferase Assay System (Promega, United States). Virus and cell controls were included for each assay. The inhibitory activity for each antibody was determined according to the relative light unit value as follows: [(relative light unit Virus control—relative light unit Cell control)—(relative light unit Antibody control—relative light unit Cell control)] / (relative light unit Virus control—relative light unit Cell control) x 100%. Neutralization is positive if at least 50% inhibition of infection is recorded.

### ACE2-inhibition assay

A flow cytometry-based assay was used to analyze the RBD-ACE2 blocking activity of the antibody samples [34,35]. Serial dilutions of antibody in PBS were mixed with biotinylated RBD at room temperature. The mixture was then incubated with human ACE2-expressing MDCK-SIAT1 cells at 4°C for 30 min. After washing, the ExtrAvidin-R-phycoerythrin protein was incubated with the cells at 4°C for 30 min. After washing, the cells were analyzed using a BD FACSCanto II flow cytometer. At least 5,000 events of RBD-bound (phycoerythrin-positive) cells were acquired for the analysis. The PBS-biotinylated RBD mixture was used to obtain maximum signal and PBS only was used to determine background. The serum with original concentration that failed to inhibit ACE2-RBD interaction was scored as negative. The ACE2-blocking titer was expressed as the reciprocal of the antibody dilution giving 50% inhibition of signal compared to maximum signal.

### Mammalian cell culture and protein expression

SARS-CoV-2 (Delta or BA.1) Spike protein sequence (14–1206, S2P) was cloned into pTT vector for expression in HEK293 EBNA (ATCC CRL-10852) suspension cells by transient transfection using FectoPRO (Polyplus) followed by 32°C incubation for 6 days. Culture supernatants were harvested and clarified by centrifugation at 6,500 g for 20 min, followed by Ni-NTA affinity (GE Healthcare) purification and Superose 6 Increase 10/300 GL (GE Healthcare) gel filtration in a buffer containing 20 mM Tris/HCl, pH 7.5, 150 mM NaCl. Antibodies were expressed in ExpiCHO cells according to manufacturers’ protocols and purified with protein A resin (Cytiva). Fab fragment was generated by papain cleavage at 37°C for 24 h, followed by protein-A-based affinity purification to remove the Fc fragment and the uncleaved IgG. The RBD domain (333–530) of SARS-CoV-2 WT Spike was constructed and cloned into pTT vector for expression in HEK293 EBNA suspension cells by transient transfection using polyethylenimine (PEI) followed by 37°C incubation for 4 days. Culture supernatants were harvested and clarified by centrifugation at 6,500 g for 20 min, followed by Ni-NTA affinity beads (GE Healthcare) and Superdex 200 Increase 10/300 GL (GE Healthcare) gel filtration.

### Cryo-EM sample preparation, data collection, processing, and model building

The ratio of Spike and Fab for complex formation as well as the incubation time was evaluated by negative stain transmission electron microscopy. Freshly purified Spike (diluted to 1 mg/ml) was mixed with Fab (2 mg/ml) at 3:1 (v/v) ratio in 20mM Tris pH 7.5, 150mM NaCl for 5 min at room temperature before being applied (3 μl) to a glow discharged Quantifoil R1.2/1.3 Cu 300 holey carbon grid mounted in a Mark IV Vitrobot (Thermo Fisher Scientific) at 4°C with 100% humidity. Grids were blotted at force 0 for 3 seconds. Data were collected on a Titan Krios G3 at 300 kV (Thermo Fisher Scientific), equipped with a Gatan K3 detector and the Gif Quantum energy filter with 20 eV slit width. Movies were acquired in EPU (Thermo Fisher Scientific, v2.10) at two exposures per hole. Total electron dose was 38 e^-^ /Å^2^ collected over 2.5 s and fractionated into 40 frames. The corresponding pixel size was 0.83 Å in the defocus range as described (**S6 Table**). Data were processed with C3 symmetry in CryoSPARC (JL-8C), or C1 symmetry in CryoSPARC (for JM-1A) or relion 3 (for JE-5C or JH-8B) with the resolution of the final map determined by gold-standard Fourier shell correlation (FSC) cutoff at 0.143 (**S11 Fig**).

### Crystallization, data collection, structure determination and analysis

Purified RBD and Fab were mixed at 1:1.15 molar ratio for 30 min incubation on ice before further purification by Superdex 200 SEC (10/300 GL, GE healthcare) in 20 mM Tris/HCl, pH 8.0, 150 mM NaCl. The complex peak was verified by 14% SDS-PAGE, pooled together and concentrated to higher than 12 mg/ml for hanging-drop vapor diffusion crystallization at 20°C. For JE-5C, crystals appeared in the condition containing 0.2 M magnesium sulfate heptahydrate, 20% (w/v) polyethylene glycol 3350. For JM-1A, crystals appeared in the condition containing 1M lithium chloride, 0.1M citrate pH 4.0 and 20% (w/v) polyethylene glycol 6,000. Crystals were harvested with diffraction datasets collected at National Synchrotron Radiation Research Center (NSRRC) TLS BL15A1 and TPS 05A beamlines. Data were processed in iMosflm. Molecular replacement was performed by Phenix Phaser-MR using structures of RBD and Fab as separate searching ensembles. The solved structure was further refined in phenix.refine with manual adjustments done in WinCoot (**S7 Table**). Structure figures were prepared with UCSF-ChimeraX and PyMOL. Footprints on RBD were determined by protein interface calculation (https://sppider.cchmc.org/) with relative surface accessibility change/loss for at least 4% upon complex formation.

## Supporting information

S1 Data Supporting information Data set file. (XLSX)

S1 Table Demographic data and sampling information of adult donors with COVID-19 vaccine and acute SARS-CoV-2 infection. (DOCX)

S2 Table Functional breadth and specificity of neutralizing anti-SARS-CoV-2 RBD monoclonal antibody from vaccinated and infected donors. (DOCX)

S3 Table Neutralizing anti-SARS-CoV-2 RBD monoclonal antibody heavy and light chain variable domain gene usage. (DOCX)

S4 Table Non-neutralizing anti-SARS-CoV-2 RBD monoclonal antibody heavy and light chain variable domain gene usage. (DOCX)

S5 Table IC_50_ values of neutralization data for Figs 2B and 4F. (DOCX)

S6 Table Cryo-EM data processing and structural refinement. (DOCX)

S7 Table X-ray diffraction data processing and structural refinement. (DOCX)

S1 Fig SARS-CoV-2 anti-RBD B cell frequencies after primary series and booster dose of COVID-19 vaccine and after infection. **(A)** Gating strategy for plasmablasts and IgM^neg^RBD^pos^ plasmablasts. **(B)** Frequencies of plasmablasts and IgM^neg^RBD^pos^ plasmablasts among donors after primary series (n = 10) and booster (n = 3) dose of COVID-19 vaccine and after infection (n = 6). B cell frequencies of two donors (donors 57, 59) after primary series of COVID-19 vaccine were unavailable because their flow cytometry files were misidentified. Each symbol represents a single donor and red line represents the mean and standard error of the mean. Kruskal-Wallis test with post-hoc Dunn’s multiple comparison was applied to analyze the difference in groups. The P value of less than 0.05 was considered significant.
(TIF)

S2 Fig Anti-RBD B-cell derived antibody clones from each donor in the study. Donors after receiving COVID-19 vaccine primary series and booster dose and COVID-19 patients were enrolled. mRNA, mRNA-1273 vaccine; adenovirus vector, ChAdOx1 vaccine; protein subunit, MVC-COV1901 vaccine. The ChAdOx1 vaccine is an adenoviral vector COVID-19 vaccine that encodes a wild-type spike including the transmembrane domain. The MVC-COV1901 is a protein subunit COVID-19 vaccine based on the stable prefusion spike adjuvanted with CpG1018 and aluminum hydroxide. The mRNA-1273 vaccine is a mRNA-based COVID-19 vaccine that encodes the prefusion stabilized full-length spike.
(TIF)

S3 Fig **Clonal sets of (A) wild type-neutralizing and (B) cross-neutralizing anti-RBD antibodies.** The clonal sets included multiple anti-RBD B cells with the same heavy and light chain variable region genes. WT, wild-type SARS-CoV-2 (Wuhan-1); Beta, Beta variant of SARS-CoV-2; Delta, Delta variant of SARS-CoV-2; VH, heavy chain variable region; VK, kappa chain variable region; V**λ**, lambda chain variable region.
(TIF)

S4 Fig **Analysis of variable region and (A) nucleotide and (B) amino acid mutations of anti-RBD antibodies.** Each symbol represents an antibody and red line represents the mean and standard error of the mean. Statistical significance among subgroups was analyzed by two-way ANOVA and Tukey’s post hoc test. The results of post hoc comparisons between subgroups were displayed in the graph. *, P < 0.05; **, P < 0.01; ***, P < 0.001; ****, P < 0.0001; ns, not significant. VH, heavy chain variable region; VL, light chain variable region; mAb, monoclonal antibody; neut, neutralizing.
(TIF)

S5 Fig Sequence alignment of RBD and epitope mapping of JE-5C and JM-1A. Sequences of RBD (partial, 333–512) of WT and Omicron subvariants are aligned and shaded for identical residues. Key variable residues of RBD are labeled and highlighted in white. RBD epitope residues for JE-5C (purple) and JM-1A (pink) are marked with triangles below the sequence, with dark colors for heavy chain and light colors light chain. Although the antibody-interacting residues overlap with the variable residues of RBD, most of the mutations do not affect their binding affinity or neutralization breadth.
(PNG)

S6 Fig Electron density map of JH-8B/RBD structure and its LCDR interactions. **(A)** Cryo-EM volume shown in mesh of the Spike/JH-8B structure at resolution 4.5Å in the interface between RBD (light grey) and JH-8B (green). (**B)** LCDR of JH-8B (green) is in vicinity of RBD (light grey), with potential interacting residues highlighted as sticks and labeled accordingly. HC, heavy chain; LC, light chain; LCDR, light chain complementarity-determining region.
(TIF)

S7 Fig Electrostatic coloring of VH3-53 antibody structures in the paratope region. Top views of the 11 VH3-53-family antibody structures are shown with red color indicating negatively charged residues and blue positively charged. HC, heavy chain; LC, light chain. PDB code 6XCM for C105, 8CWV for CC12.1, 6XC4 for CC12.3, 7ZF8 for COVOX-150, 7ZB5 for B38, 7ZF3 for Omi-3, 7ZFB for Omi-18, 8D0Z for S728-1157, and 7ZR7 for Omi42.
(PNG)

S8 Fig Binding activity of original and mutated JE-5C antibodies with RBD in ELISA. Original JE-5C antibody and site-directed mutants (Y52E, Y52Q, S56G, S56D, Y52E+S56G, Y52Q+S56G of heavy chain variable region) were tested for binding to RBD of various Omicron subvariants. Anti-RBD antibody IY-2A and anti-influenza H3 antibody BS-1A were included as controls. mAb, monoclonal antibody; VH, heavy chain variable region.
(TIF)

S9 Fig Local differences in the binding mode between JM-1A and similar antibodies. **(A-C)** JM-1A/RBD structure was superimposed with Omi-25/RBD structure with RBD shown as grey surface and Fabs in ribbon (JM-1A, pink; Omi-25, orange). CDRs and N-terminus are labeled. (**D-G**) Different local interfaces around the hotspot residue F486 of RBD in structures of BA7125 (**D**, blue), Omi-25 (**E,** orange), Beta-27 (**F**, green) and C98C7 (**G**, cyan).
(TIF)

S10 Fig JM-1A mutations may introduce possible changes in the local interface with RBD. (**A-C**) JM-1A/RBD complex structure is shown as ribbons with key residues drawn as sticks and water molecules in red spheres. Mutated site (N59F, red) and RBD hotspot residue (F486V, green) are structurally modelled. CDRs, key residues and virus strains are labeled. (**D-G**) JM-1A/RBD complex structure is shown in the same way, with mutated site (G32F, red) and RBD hotspot residues (green) structurally modelled. Virus strains are labeled at the right bottom corner of each panel. A light-yellow shade highlights the multiple-ring aromatic stacking in (**D-F**). Hydrogen bonds are drawn as blue dashed lines.
(TIF)

S11 Fig Data collection, processing, and refinement of cryo-EM structures. Raw micrographs, representative 2D classes, 3D classes, FSC curves and the fitted final map are shown for JE-5C, JH-8B, JL-8C, and JM-1A with particle number/percentage specified for each step. Cryo-EM volumes are colored white for Spike, purple for JE-5C, green for JH-8B, and blue for JL-8C, with darker colors indicating heavy chain and lighter colors light chain. All the visible RBDs in these structures adopt the up conformation.
(TIF)
