# Supplementary material for: The presence of broadly neutralizing anti-SARS-CoV-2 RBD antibodies elicited by primary series and booster dose of COVID-19 vaccine
Source: PLoS Pathog. 2024 Jun 10;20(6):e1012246. doi: 10.1371/journal.ppat.1012246 (PMC11192315; doi:10.1371/journal.ppat.1012246)
Supplement: S1 Table — (DOCX) [file ppat.1012246.s002.docx]

| **S1 Table. Demographic data and sampling information of adult donors with COVID-19 vaccine and acute SARS-CoV-2 infection.** | | | | | | |
| --- | --- | --- | --- | --- | --- | --- |
| **Post primary series of COVID-19 vaccines^#^** | | | | | | |
| **Donor** | **Gender** | **Age (yrs)** | **Dose 1** | **Dose 2** | **Sampling date** |  |
| V48 | F | 28 | mRNA-1273 | mRNA-1273 | D7 after 2nd dose |  |
| V54 | M | 40 | mRNA-1273 | mRNA-1273 | D7 after 2nd dose |  |
| V55 | M | 44 | mRNA-1273 | mRNA-1273 | D8 after 2nd dose |  |
| V59 | F | 29 | mRNA-1273 | mRNA-1273 | D7 after 2nd dose |  |
| V74 | F | 37 | mRNA-1273 | mRNA-1273 | D8 after 2nd dose |  |
| V75 | F | 36 | mRNA-1273 | mRNA-1273 | D7 after 2nd dose |  |
| V76 | F | 37 | mRNA-1273 | mRNA-1273 | D8 after 2nd dose |  |
| V84 | F | 26 | mRNA-1273 | mRNA-1273 | D7 after 2nd dose |  |
| V57 | F | 42 | ChAdOx1 | ChAdOx1 | D7 after 2nd dose |  |
| V59 | M | 40 | ChAdOx1 | ChAdOx1 | D7 after 2nd dose |  |
| V60 | F | 38 | ChAdOx1 | MVC-COV1901 | D7 after 2nd dose |  |
| V74 | F | 40 | ChAdOx1 | MVC-COV1901 | D7 after 2nd dose |  |
|  |  |  |  |  |  |  |
| **Post booster dose of COVID-19 vaccines** | | | | | | |
| **Donor** | **Gender** | **Age (yrs)** | **Dose 1** | **Dose 2** | **Booster dose** | **Sampling date** |
| V48 | F | 28 | mRNA-1273 | mRNA-1273 | mRNA-1273 | D7 after booster dose |
| V107 | F | 27 | mRNA-1273 | mRNA-1273 | mRNA-1273 | D7 after booster dose |
| V108 | F | 41 | mRNA-1273 | mRNA-1273 | mRNA-1273 | D7 after booster dose |
|  |  |  |  |  |  |  |
| **Infection** | | | | | | |
| **Donor** | **Gender** | **Age (yrs)** | **Lab-confirmed infection** | **Clinical presentation** | **Sampling date** |  |
| P1 | F | 55 | Wuhan-Hu-1 lineage | Pneumonia | D14, D22 after onset |  |
| P2 | M | 43 | Wuhan-Hu-1 lineage | Pneumonia | D14, D22 after onset |  |
| P3 | F | 42 | Wuhan-Hu-1 lineage | Febrile URI | D33 after onset |  |
| P4 | M | 32 | Wuhan-Hu-1 lineage | Febrile URI | D27 after onset |  |
| P5 | M | 42 | Wuhan-Hu-1 lineage | Febrile URI | D22 after onset |  |
| P6 | M | 58 | Wuhan-Hu-1 lineage | Febrile URI | D26 after onset |  |
|  |  |  |  |  |  |  |

^#^ The ChAdOx1 vaccine is an adenoviral vector COVID-19 vaccine that encodes a wild-type spike including the transmembrane domain. The MVC-COV1901 is a protein subunit COVID-19 vaccine based on the stable prefusion spike adjuvanted with CpG1018 and aluminum hydroxide. The mRNA-1273 vaccine is a lipid nanoparticle–encapsulated mRNA-based COVID-19 vaccine that encodes the prefusion stabilized full-length spike.
Abbreviations: yrs, years; F, female; M, male; URI, upper respiratory tract infection; D, day.
