# Supplementary material for: The presence of broadly neutralizing anti-SARS-CoV-2 RBD antibodies elicited by primary series and booster dose of COVID-19 vaccine
Source: PLoS Pathog. 2024 Jun 10;20(6):e1012246. doi: 10.1371/journal.ppat.1012246 (PMC11192315; doi:10.1371/journal.ppat.1012246)
Supplement: S2 Table — (DOCX) [file ppat.1012246.s003.docx]

**S2 Table. Functional breadth and specificity of neutralizing anti-SARS-CoV-2 RBD monoclonal antibody from vaccinated and infected donors.**

| **mAb** | **Neutralization against SARS-CoV-2^#^** | | | |  | **Binding reactivity to SARS-CoV-2 RBD^#^** | | | |  | **Cross-reactivity to other coronaviruses^#^** | | | |
| --- | --- | --- | --- | --- | --- | --- | --- | --- | --- | --- | --- | --- | --- | --- |
|  | **WT** | **Beta** | **Delta** | **Omicron BA.1** |  | **WT** | **Beta** | **Delta** | **Omicron BA.1** |  | **SARS1** | **MERS** | **OC43** | **229E** |
| **Post primary series of COVID-19 vaccines** | | | | | | | | | | | | | | |
| ***mRNA + mRNA vaccine*** | | | | | | | | | | | | | | |
| **Donor V48** | | | | | | | | | | | | | | |
| IY-7A | Neut | Non-neut | Neut | Non-neut |  | ++ | ++ | ++ | ++ |  | ++ | - | - | - |
| IY-7B | Neut | Non-neut | Neut | Non-neut |  | ++ | + | ++ | - |  | - | - | - | - |
| IY-11B | Neut | Neut | Neut | Non-neut |  | ++ | ++ | ++ | ++ |  | ++ | - | - | - |
| IY-1C | Neut | Non-neut | Non-neut | Non-neut |  | ++ | ++ | - | ++ |  | - | - | - | - |
| IY-6C | Neut | Non-neut | Neut | Non-neut |  | ++ | ++ | ++ | ++ |  | ++ | - | - | - |
| IY-8C | Neut | Neut | Neut | Non-neut |  | ++ | ++ | ++ | - |  | ++ | - | - | - |
| IY-2A | Neut | Neut | Neut | Neut |  | ++ | ++ | ++ | ++ |  | ++ | - | - | - |
| IY-8A | Neut | Neut | Neut | Non-neut |  | ++ | ++ | ++ | - |  | - | - | - | - |
| IY-9C | Neut | Non-neut | Neut | Non-neut |  | ++ | ++ | ++ | - |  | ++ | - | - | - |
| IZ-5A | Neut | Non-neut | Non-neut | Non-neut |  | ++ | ++ | ++ | ++ |  | - | - | - | - |
| IZ-11A | Neut | Non-neut | Non-neut | Non-neut |  | ++ | ++ | + | ++ |  | ++ | - | - | - |
| IZ-1B | Neut | Non-neut | Non-neut | Non-neut |  | ++ | ++ | + | - |  | - | - | - | - |
| IZ-7B | Neut | Non-neut | Non-neut | Non-neut |  | ++ | ++ | ++ | - |  | - | - | - | - |
| IZ-2C | Neut | Neut | Neut | Non-neut |  | ++ | ++ | ++ | - |  | - | - | - | - |
| **Donor V54** | | | | | | | | | | | | | | |
| IT-7A | Neut | Neut | Neut | Non-neut |  | ++ | ++ | ++ | ± |  | - | - | - | - |
| IT-11A | Neut | Non-neut | Neut | Non-neut |  | ++ | + | ++ | - |  | - | - | - | - |
| IT-3C | Neut | Non-neut | Neut | Non-neut |  | ++ | - | ++ | - |  | - | - | - | - |
| IT-4B | Neut | Non-neut | Neut | Non-neut |  | ++ | ++ | ++ | + |  | - | - | - | - |
| IT-8B | Neut | Neut | Non-neut | Non-neut |  | ++ | ++ | ++ | ++ |  | - | - | - | - |
| IT-7C | Neut | Neut | Neut | Non-neut |  | ++ | ++ | ++ | + |  | - | - | - | - |
| IT-9C | Neut | Neut | Non-neut | Non-neut |  | ++ | ++ | + | + |  | - | - | - | - |
| JH-3A | Neut | Neut | Neut | Non-neut |  | ++ | ++ | ++ | + |  | - | - | - | - |
| JH-9A | Neut | Neut | Neut | Non-neut |  | ++ | ++ | ++ | - |  | ± | - | - | - |
| JH-10A | Neut | Non-neut | Non-neut | Non-neut |  | ++ | ++ | ++ | ++ |  | - | - | - | - |
| JH-1B | Neut | Neut | Neut | Neut |  | ++ | ++ | ++ | ++ |  | - | - | - | - |
| JH-11B | Neut | Neut | Neut | Non-neut |  | ++ | ++ | ++ | - |  | - | - | - | - |
| JH-2C | Neut | Neut | Neut | Non-neut |  | ++ | ++ | ++ | + |  | - | - | - | - |
| JH-6C | Neut | Non-neut | Non-neut | Non-neut |  | + | + | + | - |  | - | - | - | - |
| JH-10C | Neut | Neut | Non-neut | Non-neut |  | ++ | ++ | ± | + |  | - | - | - | - |
| JH-11D | Neut | Neut | Non-neut | Non-neut |  | ++ | ++ | + | ± |  | - | - | - | - |
| JH-7A | Neut | Neut | Non-neut | Non-neut |  | ++ | ++ | ++ | + |  | - | - | - | - |
| JH-11A | Neut | Neut | Neut | Neut |  | ++ | ++ | ++ | ++ |  | - | - | - | - |
| JH-8B | Neut | Neut | Neut | Neut |  | ++ | ++ | ++ | ++ |  | - | - | - | - |
| JH-10B | Neut | Neut | Non-neut | Non-neut |  | ++ | ++ | ++ | + |  | - | - | - | - |
| JH-3C | Neut | Neut | Non-neut | Non-neut |  | ++ | ++ | + | + |  | - | - | - | - |
| JH-9C | Neut | Non-neut | Non-neut | Non-neut |  | ++ | ++ | ++ | + |  | - | - | - | - |
| **Donor V55** | | | | | | | | | | | | | | |
| IV-10A | Neut | Non-neut | Neut | Non-neut |  | ++ | + | ++ | - |  | - | - | - | - |
| IV-8B | Neut | Non-neut | Neut | Non-neut |  | ++ | + | ++ | ± |  | - | - | - | - |
| IV-11B | Neut | Non-neut | Neut | Non-neut |  | ++ | - | ++ | - |  | - | - | - | - |
| IV-2C | Neut | Non-neut | Neut | Non-neut |  | ++ | - | ++ | - |  | - | - | - | - |
| IV-11C | Neut | Non-neut | Neut | Non-neut |  | ++ | + | ++ | - |  | - | - | - | - |
| IV-6D | Neut | Non-neut | Neut | Non-neut |  | ++ | + | ++ | + |  | ++ | - | - | - |
| IV-7D | Neut | Non-neut | Neut | Non-neut |  | ++ | - | ++ | - |  | - | - | - | - |
| IV-5E | Neut | Non-neut | Neut | Non-neut |  | ++ | + | ++ | - |  | - | - | - | - |
| IV-8E | Neut | Non-neut | Neut | Non-neut |  | ++ | + | ++ | - |  | - | - | - | - |
| IV-12A | Neut | Non-neut | Non-neut | Non-neut |  | ++ | + | + | - |  | ++ | - | - | - |
| IV-3B | Neut | Non-neut | Non-neut | Non-neut |  | ++ | + | ++ | ± |  | - | - | - | - |
| IV-4B | Neut | Neut | Neut | Non-neut |  | ++ | ++ | ++ | - |  | + | - | - | - |
| IV-1C | Neut | Non-neut | Neut | Non-neut |  | ++ | + | ++ | - |  | ± | - | - | - |
| IV-10C | Neut | Neut | Neut | Non-neut |  | ++ | ++ | ++ | - |  | - | - | - | - |
| IV-6E | Neut | Neut | Non-neut | Non-neut |  | ++ | ++ | + | ++ |  | - | - | - | - |
| IS-9A | Neut | Neut | Neut | Non-neut |  | ++ | ++ | ++ | - |  | ++ | - | - | - |
| IS-2B | Neut | Non-neut | Neut | Non-neut |  | ++ | - | ++ | - |  | - | - | - | - |
| IS-11B | Neut | Neut | Neut | Non-neut |  | ++ | ++ | ++ | - |  | ++ | - | - | - |
| **Donor V59** | | | | | | | | | | | | | | |
| IW-2A | Neut | Neut | Neut | Non-neut |  | ++ | ++ | ++ | - |  | - | - | - | - |
| IW-4C | Neut | Non-neut | Neut | Non-neut |  | ++ | + | ++ | - |  | - | - | - | - |
| IW-11C | Neut | Neut | Neut | Neut |  | ++ | ++ | ++ | ++ |  | ++ | - | - | - |
| IW-1A | Neut | Non-neut | Non-neut | Non-neut |  | ++ | + | - | - |  | - | - | - | - |
| IW-7A | Neut | Neut | Neut | Non-neut |  | ++ | ++ | ++ | - |  | - | - | - | - |
| IW-12A | Neut | Neut | Neut | Neut |  | ++ | ++ | ++ | ++ |  | - | - | - | - |
| IW-1C | Neut | Neut | Non-neut | Non-neut |  | ++ | ++ | + | + |  | - | - | - | - |
| **Donor V74** | | | | | | | | | | | | | | |
| IX-7A | Neut | Neut | Neut | Non-neut |  | ++ | ++ | ++ | + |  | - | - | - | - |
| IX-11A | Neut | Neut | Neut | Non-neut |  | ++ | ++ | ++ | - |  | - | - | - | - |
| IX-3B | Neut | Neut | Neut | Non-neut |  | ++ | ++ | ++ | + |  | ++ | - | - | - |
| IX-5B | Neut | Non-neut | Neut | Non-neut |  | ++ | + | ++ | + |  | - | - | - | - |
| IX-12B | Neut | Non-neut | Non-neut | Non-neut |  | ++ | + | ± | - |  | - | - | - | - |
| IX-4B | Neut | Neut | Neut | Non-neut |  | ++ | ++ | ++ | - |  | - | - | - | - |
| **Donor V75** | | | | | | | | | | | | | | |
| JE-3B | Neut | Non-neut | Neut | Non-neut |  | ++ | ++ | ++ | ± |  | - | - | - | - |
| JE-5C | Neut | Neut | Neut | Neut |  | ++ | ++ | ++ | ++ |  | - | - | - | - |
| JE-2A | Neut | Neut | Neut | Neut |  | ++ | ++ | ++ | ++ |  | - | - | - | - |
| JE-5A | Neut | Neut | Neut | Neut |  | ++ | ++ | ++ | ++ |  | - | - | - | - |
| JE-6A | Neut | Neut | Non-neut | Non-neut |  | ++ | ++ | - | ++ |  | - | - | - | - |
| JE-2B | Neut | Non-neut | Neut | Non-neut |  | ++ | + | ++ | + |  | - | - | - | - |
| JE-6B | Neut | Non-neut | Neut | Non-neut |  | ++ | ++ | ++ | + |  | + | - | - | - |
| JE-1C | Neut | Neut | Non-neut | Non-neut |  | ++ | ++ | ++ | - |  | ++ | - | - | - |
| JE-4C | Neut | Neut | Neut | Neut |  | ++ | ++ | ++ | ++ |  | - | - | - | - |
| **Donor V76** | | | | | | | | | | | | | | |
| JF-11A | Neut | Neut | Neut | Non-neut |  | ++ | ++ | + | + |  | - | - | - | - |
| JF-3B | Neut | Non-neut | Neut | Non-neut |  | ++ | + | ++ | - |  | - | - | - | - |
| JF-9B | Neut | Non-neut | Neut | Non-neut |  | ++ | ± | ++ | - |  | - | - | - | - |
| JF-10B | Neut | Non-neut | Non-neut | Non-neut |  | ++ | ++ | ++ | ++ |  | ++ | - | - | - |
| JF-1C | Neut | Non-neut | Neut | Non-neut |  | ++ | - | ++ | - |  | - | - | - | - |
| JF-6C | Neut | Neut | Neut | Non-neut |  | ++ | ++ | ++ | + |  | ++ | - | - | - |
| JF-10C | Neut | Non-neut | Neut | Non-neut |  | ++ | + | ++ | - |  | - | - | - | - |
| JF-6D | Neut | Non-neut | Neut | Non-neut |  | ++ | - | ++ | - |  | - | - | - | - |
| JF-11D | Neut | Non-neut | Neut | Non-neut |  | ++ | - | ++ | - |  | - | - | - | - |
| JF-1E | Neut | Non-neut | Non-neut | Non-neut |  | ++ | ++ | ++ | ++ |  | - | - | - | - |
| JF-12B | Neut | Neut | Neut | Non-neut |  | ++ | ++ | ++ | - |  | - | - | - | - |
| JF-4C | Neut | Neut | Neut | Non-neut |  | ++ | ++ | ++ | + |  | ++ | - | - | - |
| **Donor V84** | | | | | | | | | | | | | | |
| JG-1A | Neut | Non-neut | Non-neut | Non-neut |  | ++ | ++ | + | + |  | - | - | - | - |
| JG-10A | Neut | Non-neut | Non-neut | Non-neut |  | ++ | + | ++ | ++ |  | ++ | - | - | - |
| JG-11B | Neut | Neut | Neut | Non-neut |  | ++ | ++ | ++ | - |  | - | - | - | - |
| JG-8C | Neut | Neut | Neut | Non-neut |  | ++ | ++ | ++ | - |  | - | - | - | - |
| JG-10C | Neut | Non-neut | Neut | Non-neut |  | ++ | + | ++ | - |  | - | - | - | - |
| JG-11C | Neut | Non-neut | Neut | Non-neut |  | ++ | - | ++ | - |  | - | - | - | - |
| JG-12C | Neut | Non-neut | Non-neut | Non-neut |  | ++ | ++ | + | ± |  | - | - | - | - |
| JG-2D | Neut | Non-neut | Non-neut | Non-neut |  | ++ | ++ | + | + |  | - | - | - | - |
| JG-4D | Neut | Non-neut | Non-neut | Non-neut |  | ++ | ++ | ++ | ++ |  | - | - | - | - |
| JG-6D | Neut | Neut | Non-neut | Non-neut |  | ++ | ++ | + | + |  | ++ | - | - | - |
| JG-7D | Neut | Neut | Non-neut | Non-neut |  | ++ | ++ | + | - |  | ++ | - | - | - |
| JG-11D | Neut | Neut | Non-neut | Non-neut |  | ++ | ++ | + | + |  | ++ | - | - | - |
| JG-5E | Neut | Non-neut | Neut | Non-neut |  | ++ | - | ++ | - |  | - | - | - | - |
| ***Adenovirus vector+ Adenovirus vector vaccine*** | | | | | | | | | | | | | | |
| **Donor 57** | | | | | | | | | | | | | | |
| JD-2B | Neut | Neut | Neut | Neut |  | ++ | ++ | ++ | ++ |  | - | - | - | - |
| **Donor 59** | | | | | | | | | | | | | | |
| JD-11B | Neut | Non-neut | Non-neut | Non-neut |  | ++ | - | + | - |  | - | - | - | - |
| ***Adenovirus vector + protein subunit vaccine*** | | | | | | | | | | | | | | |
| **Donor 60** | | | | | | | | | | | | | | |
| JC-7A | Neut | Neut | Neut | Neut |  | ++ | ++ | ++ | ++ |  | ++ | - | - | - |
| JC-10A | Neut | Neut | Neut | Non-neut |  | ++ | ++ | ++ | - |  | - | - | - | - |
| JC-7C | Neut | Neut | Neut | Neut |  | ++ | ++ | ++ | ++ |  | ++ | - | - | - |
| JC-6D | Neut | Non-neut | Neut | Non-neut |  | ++ | - | ++ | - |  | + | - | - | - |
| JC-9C | Neut | Neut | Neut | Non-neut |  | ++ | ++ | ++ | - |  | - | - | - | - |
| **Donor 74** | | | | | | | | | | | | | | |
| JO-8B | Neut | Neut | Neut | Non-neut |  | ++ | ++ | ++ | - |  | ++ | - | - | - |
| JO-12B | Neut | Non-neut | Neut | Non-neut |  | ++ | ++ | ++ | ++ |  | - | - | - | - |
| JO-6B | Neut | Neut | Neut | Neut |  | ++ | ++ | ++ | + |  | - | - | - | - |
| JO-2C | Neut | Neut | Neut | Neut |  | ++ | ++ | ++ | ++ |  | - | - | - | - |
| **Post booster dose of COVID-19 vaccines** | | | | | | | | | | | | | | |
| ***mRNA + mRNA + mRNA vaccine*** | | | | | | | | | | | | | | |
| **Donor V48** | | | | | | | | | | | | | | |
| JN-5A | Neut | Neut | Neut | Neut |  | ++ | ++ | ++ | ++ |  | - | - | - | - |
| JN-5B | Neut | Non-neut | Non-neut | Non-neut |  | ++ | + | + | + |  | - | - | - | - |
| JN-6B | Neut | Neut | Neut | Non-neut |  | ++ | ++ | ++ | - |  | - | - | - | - |
| **Donor V107** | | | | | | | | | | | | | | |
| JM-1A | Neut | Neut | Neut | Neut |  | ++ | ++ | ++ | ++ |  | - | - | - | - |
| JM-6A | Neut | Neut | Neut | Neut |  | ++ | ++ | ++ | ++ |  | - | - | - | - |
| JM-7A | Neut | Non-neut | Non-neut | Non-neut |  | ++ | + | + | + |  | - | - | - | - |
| JM-9A | Neut | Neut | Neut | Non-neut |  | ++ | ++ | + | - |  | - | - | - | - |
| JM-3B | Neut | Non-neut | Non-neut | Non-neut |  | ++ | ± | ± | ± |  | - | - | - | - |
| JM-7B | Neut | Neut | Neut | Non-neut |  | ++ | ++ | ++ | ± |  | ++ | - | - | - |
| JM-10B | Neut | Neut | Neut | Non-neut |  | ++ | ++ | ++ | ± |  | - | - | - | - |
| JM-2C | Neut | Neut | Neut | Non-neut |  | ++ | ++ | ++ | ± |  | - | - | - | - |
| JM-9B | Neut | Neut | Neut | Non-neut |  | ++ | ++ | ++ | ± |  | ++ | - | - | - |
| JM-7D | Neut | Neut | Neut | Non-neut |  | ++ | ++ | ++ | ± |  | - | - | - | - |
| **Donor V108** | | | | | | | | | | | | | | |
| JL-5A | Neut | Neut | Neut | Non-neut |  | ++ | ++ | ++ | - |  | - | - | - | - |
| JL-6A | Neut | Neut | Neut | Non-neut |  | ++ | ++ | ++ | ± |  | - | - | - | - |
| JL-9A | Neut | Neut | Neut | Non-neut |  | ++ | ++ | ++ | - |  | - | - | - | - |
| JL-12B | Neut | Non-neut | Neut | Non-neut |  | ++ | + | ++ | + |  | - | - | - | - |
| JL-11C | Neut | Non-neut | Non-neut | Non-neut |  | ++ | ± | - | - |  | - | - | - | - |
| JL-1D | Neut | Non-neut | Non-neut | Non-neut |  | ++ | + | + | + |  | - | - | - | - |
| JL-2B | Neut | Neut | Neut | Neut |  | ++ | ++ | ++ | ++ |  | - | - | - | - |
| JL-5B | Neut | Non-neut | Non-neut | Non-neut |  | ++ | + | + | + |  | - | - | - | - |
| JL-8B | Neut | Neut | Neut | Neut |  | ++ | ++ | ++ | ++ |  | - | - | - | - |
| JL-8C | Neut | Neut | Neut | Neut |  | ++ | ++ | ++ | ++ |  | - | - | - | - |
| **Infection** | | | | | | | | | | | | | | |
| **Donor P1** | | | | | | | | | | | | | | |
| FP-5B | Neut | Non-neut | Neut | Non-neut |  | ++ | - | ++ | - |  | - | - | - | - |
| FP-8A | Neut | Non-neut | Non-neut | Non-neut |  | ++ | - | + | - |  | - | - | - | - |
| FP-9B | Neut | Neut | Neut | Non-neut |  | ++ | ++ | ++ | ± |  | - | - | - | - |
| FP-4C | Neut | Non-neut | Neut | Non-neut |  | ++ | - | ++ | - |  | - | - | - | - |
| FP-11C | Neut | Non-neut | Neut | Non-neut |  | ++ | - | ++ | - |  | - | - | - | - |
| FY-11C | Neut | Non-neut | Non-neut | Non-neut |  | ++ | - | + | - |  | - | - | - | - |
| **Donor P2** | | | | | | | | | | | | | | |
| GI-10C | Neut | Non-neut | Neut | Non-neut |  | ++ | - | ++ | - |  | - | - | - | - |
| GM-2E | Neut | Non-neut | Neut | Non-neut |  | ++ | - | ++ | - |  | - | - | - | - |
| GP-11D | Neut | Non-neut | Non-neut | Non-neut |  | ++ | - | + | - |  | - | - | - | - |
| GZ-2D | Neut | Neut | Neut | Non-neut |  | ++ | ++ | ++ | ± |  | + | - | - | - |
| GZ-12A | Neut | Neut | Neut | Non-neut |  | ++ | + | + | ± |  | - | - | - | - |
| **Donor P3** | | | | | | | | | | | | | | |
| GQ-5B | Neut | Non-neut | Neut | Non-neut |  | ++ | + | ++ | + |  | ++ | - | - | - |
| GQ-11C | Neut | Neut | Neut | Non-neut |  | ++ | ++ | ++ | + |  | - | - | - | - |
| HC-7C | Neut | Neut | Neut | Non-neut |  | ++ | + | + | - |  | - | - | - | - |
| HC-5D | Neut | Non-neut | Neut | Non-neut |  | ++ | - | ++ | - |  | - | - | - | - |
| **Donor P4** | | | | | | | | | | | | | | |
| GR-8E | Neut | Non-neut | Non-neut | Non-neut |  | ++ | - | + | - |  | - | - | - | - |
| GX-2A | Neut | Non-neut | Non-neut | Non-neut |  | ++ | ± | + | - |  | - | - | - | - |
| **Donor P5** | | | | | | | | | | | | | | |
| GW-5D | Neut | Non-neut | Neut | Non-neut |  | ++ | - | ++ | - |  | - | - | - | - |
| **Donor P6** | | | | | | | | | | | | | | |
| HV-12E | Neut | Neut | Neut | Non-neut |  | ++ | ++ | ++ | + |  | - | - | - | - |
|  |  |  |  |  |  |  |  |  |  |  |  |  |  |  |

**^#^** The neutralizing activity of antibody (5 μg/ml) was examined in the pseudovirus-based assay using pseudotyped lentiviruses expressing the wild-type Wuhan-Hu-1, Beta variant, Delta variant or Omicron variant spike proteins. The binding activities of antibody (2 μg/ml) with SARS-CoV-2 RBDs and other coronaviruses were measured in the ELISA. The OD450 value above three times the mean absorbance of the negative control BS-1A (anti-influenza H3 human mAb) were considered positive. -, negative; ±, 0 ∼ 0.49; +, 0.50 ∼ 0.99, ++, ≥1.00.
Abbreviations: mAb, monoclonal antibody; WT, wild-type SARS-CoV-2 (Wuhan-Hu-1); Beta, Beta variant of SARS-CoV-2; Delta, Delta variant of SARS-CoV-2; Omicron BA.1, Omicron BA.1 variant of SARS-CoV-2; SARS1, SARS-CoV; MERS, Middle East respiratory syndrome–related coronavirus; OC43, human coronavirus OC43; 229E, human coronavirus 229E; Neut, neutralizing; Non-neut, non-neutralizing.
