# Supplementary material for: The presence of broadly neutralizing anti-SARS-CoV-2 RBD antibodies elicited by primary series and booster dose of COVID-19 vaccine
Source: PLoS Pathog. 2024 Jun 10;20(6):e1012246. doi: 10.1371/journal.ppat.1012246 (PMC11192315; doi:10.1371/journal.ppat.1012246)
Supplement: S3 Table — (DOCX) [file ppat.1012246.s004.docx]

**S3 Table. Neutralizing anti-SARS-CoV-2 RBD monoclonal antibody heavy and light chain variable domain gene usage.**

| **mAb** | **H-L** | **Vh** | **Jh** | **Dh** | **rf** | **V_h_ junction sequence** | **nt Mut** | **aa Sub** | **Vl** | **Jl** | **V_l_ Junction Sequence** | **nt Mut** | **aa Sub** |
| --- | --- | --- | --- | --- | --- | --- | --- | --- | --- | --- | --- | --- | --- |
| **Post primary series of COVID-19 vaccines** | | | | | | | | | | | | | |
| **Donor V48** | | | | | | | | | | | | | |
| IY-7A | H-κ | 3-66*02 F | 6*02 F | 1-26*01 F | 2 | CARDLYYYGMDVW | 13 | 7 | 1-39 or 1D-39*01 F | 1*01 or 4*01 F | CQQSYSTPPRTF | 4 | 2 |
| IY-7B | H-κ | 3-66*02 F | 6*02 F | 1-26*01 F | 2 | CARDLYYYGMDVW | 12 | 6 | 1-9*01 F | 2*01 F | CQQINSYSYTF | 1 | 0 |
| IY-11B | H-κ | 3-30*04 or 3-30-3*03 F | 4*02 F | 3-22*01 F | 2 | CARDRDDTSGYYFDYW | 9 | 5 | 1-NL1*01 F | 3*01 F | CQQYYSNPEVTF | 1 | 1 |
| IY-1C | H-κ | 1-18*01 F | 4*02 F | 3-10*01 F | 1 | CARDLGWFGELSGPSFDYW | 3 | 2 | 3-11*01 F | 4*01 F | CHQRSNPLTF | 0 | 0 |
| IY-6C | H-κ | 4-31*11 F | 4*02 F | 3-16*02 F | 1 | CAKGGGILWWFDYW | 4 | 2 | 1-33 or 1D-33*01 F | 3*01 F | CQQYDNLPPMVTF | 3 | 1 |
| IY-8C | H-κ | 3-53*04 F | 3*02 F | 2-21*01 F | 3 | CARVVSDAFDIW | 2 | 1 | 1-NL1*01 F | 2*01 F | CQQSYSIPLYTF | 5 | 4 |
| IY-2A | H-𝝺 | 4-34*01 F | 3*01 F | 3-3*02 F | 3 | CARGLGIFGVVTLSDVW | 5 | 3 | 6-57*02 F | 3*02 F | CQSYDSGIWVF | 2 | 1 |
| IY-8A | H-𝝺 | 4-39*01 F | 4*02 F | 3-10*01 F | 1 | CAKHQWLREDLDYW | 31 | 21 | 6-57*02 F | 3*02 F | CQSYDSTILWVF | 0 | 0 |
| IY-9C | H-𝝺 | 3-30*04 or 3-30-3*03 F | 4*02 F | 2-8*01 F | 2 | CARSPIGYCANGVCYPTDW | 2 | 0 | 6-57*02 F | 3*02 F | CQSYDSNIYWVF | 0 | 0 |
| IZ-5A | H-κ | 3-21*01 F | 6*02 F | 1-7*01 F | 2 | CARDGWGFRQLELRVGYYYGMDVW | 13 | 9 | 3-20*01 F | 2*01 F | CQHYASSSYTF | 5 | 3 |
| IZ-11A | H-κ | 3-43*02 F | 4*02 F | 6-6*01 F | 1 | CAKDVPTLVSSSSFPDHW | 6 | 6 | 1-39 or 1D-39*01 F | 4*01 F | CQQSYTTPLTF | 1 | 1 |
| IZ-1B | H-κ | 4-61*02 F | 3*02 F | 3-3*01 F | 3 | CARVTIFGVAGDAFDIW | 3 | 2 | 1-39 or 1D-39*01 F | 1*01 F | CQQSYSSWTF | 1 | 1 |
| IZ-7B | H-κ | 5-51*01 F | 5*02 F | 4-23*01 ORF | 3 | CARLVTPGGRGPLYNWFDPW | 2 | 2 | 3-15*01 F | 2*01 F | CQQYNNWYTF | 1 | 1 |
| IZ-2C | H-κ | 4-34*03 F | 3*02 F | 2-2*01 F | 3 | CARWAPPAIHAFDIW | 3 | 2 | 4-1*01 F | 5*01 F | CQQYYSSPITF | 3 | 3 |
| **Donor V54** | | | | | | | | | | | | | |
| IT-7A | H-κ | 3-53*01 F | 3*02 F | 1-26*01 F | 1 | CARELDIVGATDIW | 5 | 3 | 3-20*01 F | 3*01 F | CQQYGVSPPEFTF | 0 | 0 |
| IT-11A | H-κ | 3-7*01 F | 4*02 F | 3-10*01 F | 1 | CARDLGLLWFGELFGYW | 1 | 1 | 1-NL1*01 F | 4*01 F | CQQYYATPRTF | 6 | 4 |
| IT-3C | H-κ | 3-53*01 F | 6*02 F | No results | N | CARESYGMDVW | 6 | 5 | 3-20*01 F | 2*01 F | CQQYGSLYTF | 5 | 4 |
| IT-4B | H-𝝺 | 3-13*01 F | 6*02 F | 3-10*01 F | 2 | CAREVPYGSGSFNGMDVW | 1 | 1 | 3-9*01 F | 2*01 F or 3*01 F | CQVWDTSLKVVF | 5 | 4 |
| IT-8B | H-𝝺 | 3-9*01 F | 3*02 F | 3-10*01 F | 2 | CAKDYGSGNNPDAFDMW | 7 | 5 | 1-47*01 F | 3*02 F | CAAWDDSLKVF | 0 | 0 |
| IT-7C | H-𝝺 | 3-53*01 F | 3*02 F | 3-10*01 F | 3 | CARGPYSVAHDAFDIW | 4 | 3 | 1-51*01 F | 3*02 F | CGTWDSSLSAWVF | 1 | 0 |
| IT-9C | H-𝝺 | 3-9*01 F | 6*02 F | 3-10*01 F | 2 | CAKDHLTYYYGSGFPPARGDFYGMDVW | 3 | 3 | 1-51*01 F | 3*02 F | CGAWDSSLSAGVF | 0 | 0 |
| JH-3A | H-𝝺 | 3-30*03 or 18 or 3-30-5*01 F | 4*02 F | 5-18*01 F | 3 | CAKGQYSYAYPVHYFDYW | 1 | 1 | 6-57*01 F | 2*01 F or 3*01 F | CQSYDSSNAVVF | 4 | 0 |
| JH-9A | H-𝝺 | 4-39*01 F | 4*02 F | 5-12*01 F | 2 | CARLGWLRGNFDYW | 5 | 5 | 6-57*01 F | 3*02 F | CQSYDSSNQVWVF | 4 | 4 |
| JH-10A | H-𝝺 | 5-51*01 F | 3*02 F | 3-22*01 F | 2 | CASSSYYYDTTSRSGAFDIW | 6 | 5 | 1-44*01 F | 3*02 F | CAAWDDSLNGWVF | 0 | 0 |
| JH-1B | H-𝝺 | 3-9*01 or 03 F | 6*02 F | 3-16*01 F | 3 | LYLQMNSLRAEDTALYYCAKDMRFGGTYTTEDYGMDVW | 1 | 1 | 3-21*03 F | 2*01 or 3*01 F | CQVWDSSSDRVVF | 1 | 0 |
| JH-11B | H-𝝺 | 1-18*01 F | 4*02 F | 3-10*01 F | 2 | CAREERGYYGSGSYYAFDYW | 3 | 2 | 6-57*04 F | 2*01 or 3*01 F | CQSYHGTSYVVF | 3 | 2 |
| JH-2C | H-𝝺 | 5-51*01 F | 5*02 F | 5-12*01 F | 3 | CVRLDSGYRNWFDPW | 5 | 2 | 1-44*01 F | 1*01 F | CAAWDDSLNGYVF | 4 | 3 |
| JH-6C | H-𝝺 | 3-9*01 F | 6*02 F | 3-10*01 F | 1 | CAKDIRRWFGEIGGMDVW | 9 | 5 | 3-21*03 F | 2*01 F or 3*01 F | CQVWDSRSDHVVF | 2 | 0 |
| JH-10C | H-𝝺 | 3-9*01 F | 6*02 F | 5-12*01 F | 3 | CAKFAGYSAYDFTSVVVAATYNYYGMDVW | 3 | 3 | 1-51*01 F | 3*02 F | CGTWESNLSAEGWVF | 1 | 1 |
| JH-11D | H-𝝺 | 3-9*01 F | 3*02 F | 3-10*01 F | 2 | CAKDHSRLDYYGSGSQGVAFDIW | 6 | 6 | 1-51*01 F | 3*02 F | CGTWDSSLSAGVF | 1 | 0 |
| JH-7A | H-κ | 3-30*03 or 18 or 3-30-5*01 F | 4*02 F | 5-24*01 ORF | 1 | CAKERGMTTTHFDYW | 6 | 5 | 1-NL1*01 F | 1*01 F | CQQYYGTPPWTF | 0 | 0 |
| JH-11A | H-κ | 3-53*01 F | 3*02 F | 1-26*01 F | 1 | CARELDIVGATDIW | 5 | 3 | 3-20*01 F | 3*01 F | CQQYGVSPPEFTF | 1 | 0 |
| JH-8B | H-κ | 3-53*01 F | 3*02 F | 1-26*01 F | 1 | CARELDIVGATDIW | 5 | 3 | 3-20*01 F | 3*01 F | CQQYGVSPPEFTF | 3 | 1 |
| JH-10B | H-κ | 4-59*01 F | 2*01 F | 2-2*01 F | 2 | CARVPPVGDCSSPSCQDWYFDLW | 5 | 4 | 1-5*03 F | 2*01 F or 2*02 F | CQQYNSYSTF | 0 | 0 |
| JH-3C | H-κ | 3-23 or 3-23D*01 F | 2*01 F | 2-21*02 F | 3 | CAKEATSVHWYFDLW | 8 | 6 | 1-39 or 1D-39*01 F | 5*01 F | CQQSYSTPPITF | 5 | 3 |
| JH-9C | H-κ | 3-48*02 F | 6*02 F | 3-16*01 F | 1 | CARVLGQLSYYYYGMDVW | 3 | 3 | 1-39 or 1D-39*01 F | 4*01 F | CQQSYSTPATF | 6 | 4 |
| **Donor V55** | | | | | | | | | | | | | |
| IV-10A | H-κ | 3-53*01 F | 4*02 F | 1-26*01 F | 1 | CARDLGPVGATDYW | 2 | 2 | 3-20*01 F | 1*01 F | CQQYDSSPGTF | 4 | 4 |
| IV-8B | H-κ | 3-53*01 F | 6*02 F | 3-9*01 F | 2 | CARWGRVSGYDILTGYYKGFRDYYGMDVW | 4 | 3 | 1-5*03 F | 2*01 F | CQQHNSYSYTF | 0 | 0 |
| IV-11B | H-κ | 3-53*01 F | 6*02 F | 3-9*01 F | 2 | CARDLDILGGMDVW | 2 | 2 | 1-39 or 1D-39*01 F | 4*01 F | CQQSYSTPNTF | 1 | 1 |
| IV-2C | H-κ | 1-46*01 or 03 F | 5*02 F | 2-2*01 F | 3 | CAGSIVDPYWFDPW | 3 | 3 | 1-12*01 or 02 or 1D-12*02 | 4*01 F | CQQANSFPLALTF | 0 | 0 |
| IV-11C | H-κ | 4-4*07 F | 4*02 F | 2-21*01 F | 3 | CARDLSRGGIVVVGDYW | 3 | 3 | 4-1*01 F | 5*01 F | CQQYYSTPITF | 0 | 0 |
| IV-6D | H-κ | 3-9*01 F | 4*02 F | 5-18*01 F | 1 | CAKGHTYTAMLRMAFDYW | 7 | 4 | 3-15*01 F | 5*01 F | CQQYNNWPPSITF | 0 | 0 |
| IV-7D | H-κ | 3-53*01 F | 4*02 F | 6-19*01 F | 2 | CARDLEEVGGFDCW | 2 | 1 | 3-20*01 F | 5*01 F | CQLYGSSPPITF | 2 | 2 |
| IV-5E | H-κ | 3-53*01 F | 6*02 F | 6-13*01 F | 2 | CARDLAAAGGMDVW | 4 | 2 | 1-9*01 F | 2*01 F | CQQLISYPYTF | 3 | 2 |
| IV-8E | H-κ | 3-53*01 F | 3*02 F | 3-10*01 F | 3 | CARLSSMVRGVDAFDIW | 2 | 2 | 4-1*01 F | 4*01 F | CQQYYSGLSLTF | 1 | 1 |
| IV-12A | H-𝝺 | 4-59*01 F | 4*02 F | 5-12*01 F | 3 | CAATLGVDYYCDYW | 7 | 6 | 2-14*01 or 03 F | 2*01 F | CSSYTSSSTLVVF | 2 | 1 |
| IV-3B | H-𝝺 | 1-2*02 F | 4*02 F | 1-26*01 F | 3 | CARDRFSDSGSPSFYFDSW | 3 | 3 | 2-14*03 F | 2*01 F | CSSYTSSSTLDVVF | 1 | 1 |
| IV-4B | H-𝝺 | 3-9*01 F | 4*02 F | 3-10*01 F | 1 | CAKGRLGELLFPVDYW | 5 | 4 | 1-44*01 F | 3*02 F | CAAWDDSLNGWVF | 2 | 1 |
| IV-1C | H-𝝺 | 3-9*01 F | 4*02 F | 3-10*01 F | 1 | CAKGRLGELLFPVDYW | 2 | 1 | 1-44*01 F | 3*02 F | CAAWDDSLNGWVF | 1 | 1 |
| IV-10C | H-𝝺 | 4-39*07 F | 4*02 F | 3-10*01 F | 1 | CATLLWLRGYFDYW | 4 | 4 | 6-57*01 F | 3*02 F | CQSYDSSNWVF | 0 | 0 |
| IV-6E | H-𝝺 | 1-69*10 F | 1*01 F | 3-22*01 F | 2 | CARVGYYDSSGYLAAEYFQHW | 2 | 2 | 1-40*01 F | 2*01 F | CQSYDSSLIGPVF | 5 | 4 |
| IS-9A | H-κ | 5-10-1*03 F | 4*02 F | 3-22*01 F | 2 | CARGRNYLDSRGRFDYW | 6 | 4 | 1-33 or 1D-33*01 F | 3*01 F | CQQYDSLGFTF | 1 | 1 |
| IS-2B | H-κ | 3-53*04 F | 4*02 F | 3-16*01 F | 3 | CARLAGGRKQYDYW | 5 | 4 | 1-33 or 1D-33*01 F | 5*01 F | CQQYDNLPITF | 1 | 1 |
| IS-11B | H-κ | 5-10-1*03 F | 4*02 F | 3-22*01 F | 2 | CARGRSYYDSRGRFDYW | 2 | 2 | 1-33 or 1D-33*01 F | 3*01 F | CQQYDSLGFTF | 2 | 2 |
| **Donor V59** | | | | | | | | | | | | | |
| IW-2A | H-κ | 3-30*03 or 18 or 3-30-5*01 F | 3*02 F | 6-19*01 F | 1 | CASNSYSSGWYPVEAFDIW | 2 | 2 | 1-16*02 F | 4*01 F | CQQYTTYPLTF | 5 | 3 |
| IW-4C | H-κ | 4-39*01 F | 4*02 F | 2-15*01 F | 3 | CARSLGAADYW | 7 | 6 | 1-NL1*01 F | 2*01 F or 3*01 F | CQQYYSTPPFTF | 4 | 4 |
| IW-11C | H-κ | 3-15*01 F | 4*02 F | 5-24*01 ORF | 3 | CTTDAGYTYSPDYW | 1 | 1 | 1-39 or 1D-39*01 F | 1*01 F | CQQSYSTTWTF | 4 | 3 |
| IW-1A | H-𝝺 | 4-34*01 F | 4*02 F | 6-13*01 F | 2 | CARGPGIETHGPARAGDYW | 0 | 0 | 7-43*01 F | 3*02 F | CLLYFGGAQPWVF | 4 | 3 |
| IW-7A | H-𝝺 | 3-21*01 F | 4*02 F | 2-2*01 F | 3 | CARHQSFLPMPDYW | 1 | 0 | 6-57*04 F | 3*02 F | CQSYDSSNQVF | 3 | 2 |
| IW-12A | H-𝝺 | 3-9*01 F | 4*02 F | 2-15*01 F | 1 | CAKDIQSTSLWWELLPNYFDYW | 7 | 5 | 2-14*01 or 03 F | 2*01 F or 3*01 F | CSSYTSSSTLVF | 5 | 5 |
| IW-1C | H-𝝺 | 3-9*01 F | 3*02 F | 3-22*01 F | 2 | CAKDIPDSDYYYDSTGYGGFDIW | 3 | 3 | 1-47*01 F | 3*02 F | CAAWDDSLSALVF | 1 | 1 |
| **Donor V74** | | | | | | | | | | | | | |
| IX-7A | H-κ | 3-30-3*01 F | 4*02 F | 3-10*02 F | 2 | CAREHFRAGDYW | 5 | 5 | 1-39 or 1D-39*01 F | 1*01 F | CQQSYSPLWTF | 5 | 3 |
| IX-11A | H-κ | 3-7*01 F | 6*03 F | 6-13*01 F | 3 | CARHGQQLVPYYYYFYYMDVW | 6 | 3 | 3-15*01 F | 4*01 F | CQQYINWPPVTF | 3 | 3 |
| IX-3B | H-κ | 3-13*01 F | 6*03 F | 6-13*01 F | 1 | CARGGSSWLYYYMDVW | 7 | 3 | 1-39 or 1D-39*01 F | 3*01 F | CQQTYSMPGFTF | 4 | 4 |
| IX-5B | H-κ | 3-66*02 F | 4*02 F | 5-12*01 F | 3 | CARGLYAYSGYDYNYW | 5 | 5 | 1-33 or 1D-33*01 F | 1*01 or 3*01 F | CQQYDNLPPAF | 1 | 1 |
| IX-12B | H-κ | 3-7*01 F | 6*03 F | 2-2*01 F | 2 | CARRGGYCTNTNCYSRYYYMDVW | 11 | 8 | 1-12*01 or 02 or 1D-12*02 F | 4*01 F | CQQANSFPLTF | 2 | 1 |
| IX-4B | H-𝝺 | 3-66*01 or 02 or 3-66*04 F | 6*03 F | 3-3*01 F | 2 | CARDTYFDSWSGYGDYYHYYMDVW | 6 | 4 | 1-40*01 F | 3*02 F | CQSYDSSLSGPNWVF | 2 | 2 |
| **Donor V75** | | | | | | | | | | | | | |
| JE-3B | H-κ | 1-69 or 1-69D*01 F | 4*02 F | 2-21*02 F | 2 | CARGRGGDEKVYFFDYW | 8 | 6 | 1-6*01 F | 1*01 F | CLQDYNYPWTF | 1 | 0 |
| JE-5C | H-κ | 3-53*04 F | 6*02 F | 3-16*01 F | 1 | CARDLREAGGMDVW | 5 | 2 | 1-33 or 1D-33*01 F | 1*01 F | CQQFDNLPWTF | 1 | 1 |
| JE-2A | H-𝝺 | 3-9*01 F | 6*02 F | 6-13*01 F | 1 | CAKDISRSRSWYEIYYYGMDVW | 9 | 5 | 3-21*03 F | 2*01 or 3*01 F | CQVWDSSSDHVVF | 1 | 1 |
| JE-5A | H-𝝺 | 3-53*01 F | 6*02 F | 3-10*01 F | 2 | CARDLMVRGGMDVW | 5 | 4 | 3-21*03 F | 2*01 or 3*01 F | CQVWDGSSDHVVF | 2 | 2 |
| JE-6A | H-𝝺 | 3-30-3*01 F | 3*02 F | 3-10*01 F | 2 | CARGDYYGSGSYYNPLPAFDIW | 12 | 8 | 1-44*01 F | 3*02 F | CATWDDSLNGWVF | 4 | 3 |
| JE-2B | H-𝝺 | 3-15*01 F | 4*02 F | 3-22*01 F | 2 | CTTEFSISGYHYDWPYFFDYW | 8 | 7 | 2-14*03 F | 1*01 F | CSSYRSSSTPYVF | 2 | 0 |
| JE-6B | H-𝝺 | 4-39*01 F | 4*02 F | 6-13*01 F | 1 | CARHLGSSWYGGVDYW | 7 | 6 | 3-21*03 F | 2*01 or 3*01 F | CQVWDSSSDHVVF | 2 | 1 |
| JE-1C | H-𝝺 | 3-30*04 or 15 or 3-30-3*03 F | 5*02 F | 6-13*01 F | 2 | CARGRTSAPEHNWFDPW | 12 | 8 | 3-21*03 F | 1*01 F | CQVWDSSGDHYVF | 5 | 3 |
| JE-4C | H-𝝺 | 3-9*01 F | 6*02 F | 5-12*01 F | 3 | CAKFGFKSVFDYRKADYFYGMDVW | 4 | 4 | 3-21*03 F | 1*01 F | CQVWDSSSEHYVF | 1 | 1 |
| **Donor V76** | | | | | | | | | | | | | |
| JF-11A | H-κ | 1-2*02 F | 4*02 F | 3-10*01 F | 2 | CARDDLYRGSGSYSWPFYFDYW | 4 | 4 | 1-33 or 1D-33*01 F | 3*01 F | CQQFDNLPITF | 2 | 1 |
| JF-3B | H-κ | 3-30-3*01 F | 3*01 F | 2-15*01 F | 3 | CARAEDVVVVAGGMNVW | 7 | 5 | 3-20*01 F | 4*01 F | CQQYGSSPLTF | 0 | 0 |
| JF-9B | H-κ | 1-46*01 or 03 F | 4*02 F | 5-24*01 ORF | 1 | CTRDLIKLGEMATITTDDFW | 8 | 7 | 1-5*03 F | 1*01 F | CQQYDSYWTF | 1 | 0 |
| JF-10B | H-κ | 3-30*09 or 3-30-3*01 F | 6*02 F | 3-10*01 F | 3 | CARSLKVRGIIATYYYYGMDVW | 6 | 4 | 1-33 or 1D-33*01 F | 2*01 F | CQQYDNLPYTF | 0 | 0 |
| JF-1C | H-κ | 3-53*01 F | 6*02 F | No results |  | CARDLDYYGMDVW | 6 | 6 | 1-9*01 F | 1*01 F | CQQLNSYPSGWTF | 1 | 1 |
| JF-6C | H-κ | 3-43*02 F | 4*02 F | 3-22*01 F | 3 | CAKDMGTTMIVVVFDYW | 6 | 6 | 1-39 or 1D-39*01 F | 4*01 F | CQQSYSTPPLTF | 0 | 0 |
| JF-10C | H-κ | 3-7*01 F | 5*02 F | 3-10*01 F | 1 | CARDLGILWFGELLFPSPGPR (TRP 118 not identified) | 2 | 1 | 1-NL1*01 F | 1*01 F | CQQYYSIPRTF | 2 | 2 |
| JF-6D | H-κ | 1-46*01 or 03 F | 4*02 F | 5-24*01 ORF | 1 | CARDLIKLGEMATIKTDDYW | 6 | 5 | 1-5*03 F | 1*01 F | CHQYDSYWTF | 2 | 2 |
| JF-11D | H-κ | 3-53*04 F | 6*02 F | 3-9*01 F | 3 | CARDLVVSGLDVW | 4 | 3 | 1-9*01 F | 2*01 F | CQQLNSDSYTF | 0 | 0 |
| JF-1E | H-κ | 3-33*01 or 06 F | 6*02 F | 2-15*01 F | 3 | CVGDWEIVVAGMDVW | 7 | 5 | 2-30*02 F | 2*01 or 02 F | CMQGTHWPPTF | 0 | 0 |
| JF-12B | H-𝝺 | 1-18*01 F | 5*02 F | 2-8*01 F | 3 | CARRQIASWFDPW | 3 | 3 | 6-57*02 F | 2*01 or 3*01 or 02 F | CQSYDGINQEVF | 0 | 0 |
| JF-4C | H-𝝺 | 3-7*01 F | 4*02 F | 3-10*01 F | 1 | CATNTEWFGELSAYFDYW | 6 | 4 | 3-1*01 F | 2*01 or 3*01 F | CQAWDSSTYVVF | 1 | 1 |
| **Donor V84** | | | | | | | | | | | | | |
| JG-1A | H-κ | 3-73*01 F | 4*02 F | 4-17*01 F | 2 | CTRPLDYGESDFDYW | 1 | 1 | 1-6*01 F | 1*01 F | CLQDYNYPWTF | 6 | 4 |
| JG-10A | H-κ | 3-30*04 or 3-30-3*03 F | 2*01 F | 5-18*01 F | 1 | CAREGYADISMYFDLW | 7 | 4 | 1-39 or 1D-39*01 F | 5*01 F | CQQSYSTPQVTF | 1 | 1 |
| JG-11B | H-κ | 5-51*01 F | 3*02 F | 6-13*01 F | 2 | CARTLAAAGRGDSDSFDIW | 4 | 4 | 1-5*01 F | 1*01 F | CQQYDSFSSWTF | 1 | 1 |
| JG-8C | H-κ | 3-9*01 or 02 or 04 F | 4*02 F | 3-10*01 F | 3 | CAKDRGSLRGVIVNW | 17 | 12 | 1-5*01 F | 3*01 F | CQHYNAYSASTF | 6 | 5 |
| JG-10C | H-κ | 3-53*04 F | 6*02 F | 4-23*01 ORF | 2 | CARDLGPSGGMDVW | 7 | 4 | 3-15*01 F | 1*01 F | CQQYNDWPPGTF | 2 | 1 |
| JG-11C | H-κ | 3-66*02 F | 3*02 F | 3-10*01 F | 1 | CARDLEEAGAFDIW | 8 | 7 | 1-27*01 F | 4*01 F | CQKYNSAPLTF | 1 | 1 |
| JG-12C | H-κ | 3-11*01 F | 3*02 F | 5-18*01 F | 1 | CARELFPTMGNDAFDIW | 4 | 3 | 1-39 or 1D-39*01 F | 3*01 F | CQQSYSTPRTF | 1 | 1 |
| JG-2D | H-κ | 3-13*01 F | 3*02 F | 1-7*01 F | 1 | CARGYITATTWAFDIW | 2 | 2 | 1-39 or 1D-39*01 F | 4*01 F | CQQSYSNPPATF | 6 | 4 |
| JG-4D | H-κ | 3-23*04 F | 4*02 F | 2-2*01 F | 2 | CAKGPRSNSNYFDYG (TRP 118 not identified) | 25 | 17 | 1-5*01 F | 1*01 F | CQQYNSYSGTF | 3 | 3 |
| JG-6D | H-κ | 3-23*04 F | 4*02 F | 6-19*01 F | 1 | CAKGPRYSSDYFEYW | 7 | 6 | 1-5*01 F | 4*01 or 02 F | CQQYNSLSF | 0 | 0 |
| JG-7D | H-κ | 3-11*01 F | 3*02 F | 2-2*01 F | 3 | CARELFPTEGNDAFDIW | 3 | 1 | 1-39 or 1D-39*01 F | 1*01 F | CQQSYSTPRTF | 1 | 1 |
| JG-11D | H-κ | 3-23*04 F | 4*02 F | 2-21*01 F | 3 | CAKDVRGSVDYFDYW | 12 | 7 | 1-5*01 F | 4*01 F | CQQYNSYSLTF | 0 | 0 |
| JG-5E | H-κ | 3-66*02 F | 6*02 F | 4-11*01 ORF | 1 | CARDLQERGGMDVW | 5 | 3 | 1-33 or 1D-33*01 F | 2*01 F | CQQYGNLPYTF | 3 | 2 |
| **Donor 57** | | | | | | | | | | | | | |
| JD-2B | H-𝝺 | 3-9*01 F | 3*01 or 02 F | 3-3*01 F | 2 | CAKLAGPSYYDFLRSGGDAFDLW | 6 | 4 | 1-44*01 F | 1*01 F | CAVWDDSLYVF | 0 | 0 |
| **Donor 59** | | | | | | | | | | | | | |
| JD-11B | H-κ | 1-69*09 or 17 F | 4*02 F | 3-22*01 F | 2 | CARTGYYYDSSGYYSDYW | 9 | 7 | 3-20*01 F | 1*01 F | CHQYGSSPRTF | 4 | 3 |
| **Donor 60** | | | | | | | | | | | | | |
| JC-7A | H-κ | 3-30*03 or 18 or 3-30-5*01 F | 4*02 F | 6-13*01 F | 1 | CAKDFGVSNFYDVFDYW | 14 | 10 | 1-39*01 F | 1*01 F | CQQSYSTPPWTF | 3 | 3 |
| JC-10A | H-κ | 4-31*03 F | 6*02 F | 5-18*01 F | 2 | CARDRGIQLWLGGSPGGMDVW | 15 | 10 | 3-20*01 F | 2*01 F | CQQYGSSPYTF | 2 | 0 |
| JC-7C | H-κ | 3-30-3*01 F | 4*02 F | 2-21*01 F | 3 | CARDSEQIVVVMDYW | 10 | 7 | 1-39 or 1D-39*01 F | 4*01 F | CQQSYETPPLTF | 8 | 6 |
| JC-6D | H-κ | 3-30-3*01 F | 4*02 F | 3-10*01 F | 3 | CARDGSLFGLVRGIMAPSFDYW | 8 | 5 | 1-33 or 1D-33*01 F | 4*01 F | CQQYDNLPPTF | 9 | 8 |
| JC-9C | H-𝝺 | 1-18*01 F | 4*02 F | 3-10*01 F | 2 | CASGLGTPYYW | 10 | 6 | 6-57*02 F | 3*02 F | CQSYDTMSWVF | 1 | 1 |
| **Donor 74** | | | | | | | | | | | | | |
| JO-8B | H-κ | 3-9*01 F | 4*02 F | 2-15*01 F | 3 | CAKDKNVVLVAAPEFDYW | 5 | 3 | 1-9*01 F | 4*01 F | CQQFKSF | 1 | 1 |
| JO-12B | H-κ | 3-43*01 F | 4*02 F | 3-10*01 F | 1 | CAGGKFGELFPPDYW | 2 | 2 | 1-39 or 1D-39*01 F | 1*01 F | CQQSYSNMWTF | 4 | 2 |
| JO-6B | H-𝝺 | 3-53*01 F | 4*02 F | 4-17*01 F | 2 | CARVLPFGDYVDYW | 8 | 6 | 1-40*01 F | 1*01 F | CQSYDSSLSGFYVF | 4 | 3 |
| JO-2C | H-𝝺 | 4-39*01 F | 3*02 F | 3-22*01 F | 2 | CARFDWYYYDSSGQRRDAFDIW | 5 | 5 | 1-51*01 F | 2*01 or 3*01 F | CGTWDSSLSAYVVF | 4 | 4 |
| **Post booster dose of COVID-19 vaccines** | | | | | | | | | | | | | |
| **Donor V48** | | | | | | | | | | | | | |
| JN-5A | H-κ | 3-43*02 F | 6*02 F | 3-16*01 F | 3 | CAKDGSFVGFDGMDVW | 7 | 6 | 3-20*01 F | 1*01 F | CQQYGSSPLTF | 9 | 4 |
| JN-5B | H-κ | 3-13*01 F | 6*03 F | 1-26*01 F | 1 | CVRGVGATTHYYYYYMDVW | 9 | 5 | 1-39 or 1D-39*01 F | 1*01 F | CQQSYATPPWTF | 7 | 6 |
| JN-6B | H-𝝺 | 3-7*01 F | 4*02 F | 3-9*01 F | 2 | CARVTLIWTGADYW | 11 | 6 | 6-57*02 F | 3*02 F | CQTYDSSNWVF | 2 | 2 |
| **Donor V107** | | | | | | | | | | | | | |
| JM-1A | H-κ | 4-4*10 F | 4*02 F | 3-16*01 F | 1 | CARMRGIFDYW | 17 | 13 | 1-NL1*01 F | 4*01 F | CQHYYSTPFF | 5 | 4 |
| JM-6A | H-κ | 4-39*03 F | 4*02 F | 6-6*01 F | 2 | CARSLGIIDYW | 21 | 13 | 1-NL1*01 F | 5*01 F | CQQYFGSPPITF | 4 | 3 |
| JM-7A | H-κ | 3-13*01 F | 6*03 F | 6-19* 01 F | 1 | CARVIHSSGYYRASYYYYMDLW | 11 | 9 | 1-39 or 1D-39*01 F | 5*01 F | CQQSYSNPSSTF | 9 | 7 |
| JM-9A | H-κ | 1-58*01 F | 3*02 F | 2-15*01 F | 2 | CAAPGCGTICPDGFDIW | 7 | 4 | 3-20*01 F | 1*01 F | CQQYGSSPWTF | 2 | 2 |
| JM-3B | H-κ | 4-34*01 F | 6*02 F | 3-3*01 F | 2 | CARDSPHLGNYDFWSGRTRDYDYGMDVW | 15 | 11 | 1-39 or 1D-39*01 F | 1*01 F | CQQSYSTPRTF | 6 | 3 |
| JM-7B | H-κ | 3-30*04 or 3-30-3*03 F | 1*01 F | 6-13*01 F | 3 | CARESGQQQLVEDFQYW | 11 | 7 | 1-39 or 1D-39*01 F | 2*01 F | CQQSYSTPPYTF | 4 | 1 |
| JM-10B | H-κ | 3-30*04 or 3-30-3*03 F | 4*02 F | 4-23*01 ORF | 1 | CARERLRGGDYW | 12 | 7 | 1-39 or 1D-39*01 F | 1*01 F | CQQSFSPLWTF | 5 | 4 |
| JM-2C | H-κ | 1-18*01 F | 6*03 F | 1-14*01 ORF | 3 | CATDKGIMDVW | 20 | 13 | 1-NL1*01 F | 1*01 F | CQQYYSPPPRTF | 5 | 4 |
| JM-9B | H-𝝺 | 3-7*01 F | 3*02 F | 6-13*01 F | 2 | CATLAAAGQNVYPFRPNDGFDIW | 11 | 5 | 3-25*03 F | 3*02 F | CQSGDSSHWVF | 11 | 6 |
| JM-7D | H-𝝺 | 4-59*01 F | 4*02 F | 3-3*01 F | 3 | CARGGGAFGVVTNFDFW | 15 | 7 | 6-57*04 F | 1*01 F | CQSYDGSHYVF | 3 | 2 |
| **Donor V108** | | | | | | | | | | | | | |
| JL-5A | H-κ | 1-58*01 F | 3*02 F | 2-2*01 F | 2 | CAAPHCNRTTCSDGFDIW | 17 | 9 | 3-20*01 F | 1*01 F | CQQYGTSPWTF | 7 | 4 |
| JL-6A | H-κ | 4-39*01 F | 4*02 F | 4-17*01 F | 2 | CARAYGFFDYW | 21 | 17 | 1-NL1*01 F | 4*01 F | CQQYYSAPPLTF | 8 | 7 |
| JL-9A | H-κ | 3-33*01 or 06 F | 3*02 F | 2-15*01 F | 2 | CARDCSGGGCNGDIFDIW | 11 | 5 | 3-11*01 F | 3*01 F | CRLRSNWPIRWVTF | 8 | 4 |
| JL-12B | H-κ | 4-30-4*01 F | 4*02 F | 3-22*01 F | 2 | CARGVAEYSFDRSGYHFDYW | 14 | 9 | 1-33 or 1D-33*01 F | 3*01 F | CQQSDNFPRF | 10 | 4 |
| JL-11C | H-κ | 1-69*06 F | 6*02 F | 5-12*01 F | 1 | CARDFRDIVATIPYHNYFYGMGVW | 8 | 7 | 1-33 or 1D-33*01 F | 3*01 F | CQQYDNLPLTF | 4 | 4 |
| JL-1D | H-κ | 3-23*04 F | 4*02 F | 3-9*01 F | 2 | CAKASRSNTDYFDFW | 22 | 13 | 1-5*01 F | 1*01 F | CQQYVTYLGTF | 4 | 3 |
| JL-2B | H-𝝺 | 5-51*01 F | 6*02 F | 5-18*01 F | 3 | CARHENLYFGMDVW | 3 | 3 | 1-44*01 F | 3*02 F | CAAWDDSLNGWVF | 10 | 8 |
| JL-5B | H-𝝺 | 1-46*01 or 02 or 03 F | 1*01 F | 2-2*01 F | 2 | CARDSGYCTSSRCYPAEYFQYW | 8 | 5 | 3-21*03 F | 3*02 F | CQVWDSTSDHPIFVF | 4 | 2 |
| JL-8B | H-𝝺 | 3-30*03 or 18 or 19 or 3-30-5*01 or 3-33*05 F | 4*02 F | 3-3*01 F | 2 | CAKDPNLELIWSGYFDYW | 16 | 10 | 3-21*03 F | 3*02 F | CQVWDSSSDHFWVF | 9 | 5 |
| JL-8C | H-𝝺 | 5-51*01 F | 6*02 F | 3-9*01 F | 2 | CARRTDQYGSHGMDVW | 18 | 10 | 1-44*01 F | 2*01 or 3*01 F | CAAWDDSLNGVLF | 13 | 8 |
| **Infection** | | | | | | | | | | | | | |
| **Donor P1** | | | | | | | | | | | | | |
| FP-5B | H-𝝺 | 1-2*02 F | 4*02 F | 3-3*01 F | 2 | CAREPYYDILTGLINRYYFDYW | 0 | 0 | 2-14*01 F | 3*02 F | CSSYTSSSTRVF | 1 | 1 |
| FP-8A | H-κ | 4-31*03 F | 1*01 F | 2-21*02 F | 1 | CASTARPLTNAEYFQHW | 4 | 3 | 3-20*01 F | 1*01 F | CQQYRTF | 2 | 1 |
| FP-9B | H-κ | 1-18*04 F | 4*02 F | 2-2*01 F | 3 | CASSPLVVPAAMYDYW | 2 | 1 | 3D-20*01 F | 4*01 F | CQQYGSSPLTF | 0 | 0 |
| FP-4C | H-κ | 3-53*01 F | 1*01 F | 3-10*01 F | 3 | CARHITMVRGVIIYLKEW | 0 | 0 | 1-33*01 or 1D-33*01 F | 5*01 F | CQQCDNLPITF | 0 | 0 |
| FP-11C | H-κ | 3-30*04 or 3-30-3*03 F | 4*02 F | 3-10*01 F | 3 | CARDGVGTMVRGVMSPLFDYW | 3 | 3 | 1-33*01 or 1D-33*01 F | 2*01 F | CQQYDNLPYTF | 0 | 0 |
| FY-11C | H-κ | 3-53*01 F | 6*02 F | 2-21*02 F | 3 | CARDLVTYGMDVW | 0 | 0 | 1-9*01 F | 4*01 or 02 F | CQQLNSYPLF | 0 | 0 |
| **Donor P2** | | | | | | | | | | | | | |
| GI-10C | H-𝝺 | 3-53*01 F | 2*01 F | 1-1*01 F | 3 | CARDINWNDVNWYFDLW | 0 | 0 | 1-40*01 F | 3*02 F | CQSYDSSLEWRVF | 1 | 1 |
| GM-2E | H-κ | 3-66*02 F | 5*02 F | 1-14*01 F | 3 | CARDVRDHLW | 0 | 0 | 1-33*01 or 1D-33*01 F | 1*01 F | CQQYDNLPPTF | 2 | 1 |
| GP-11D | H-κ | 3-66*02 F | 4*02 F | 4-17*01 F | 3 | CARETTGFDYW | 2 | 2 | 1-9*01 F | 1*01 F | CQQLNSYPPVSTF | 0 | 0 |
| GZ-2D | H-κ | 4-34*01 F | 3*02 F | 3-22*01 F | 2 | CARGGWIYYDSLHNAFDIW | 0 | 0 | 4-1*01 F | 1*01 F | CQQYYSTPQTF | 0 | 0 |
| GZ-12A | H-𝝺 | 1-69*09 F | 1*01 F | 3-22*01 F | 2 | CARERGYYYDSSGSLPAEYFQHW | 0 | 0 | 1-40*01 F | 3*02 F | CQSYDSSLSGPVF | 0 | 0 |
| **Donor P3** | | | | | | | | | | | | | |
| GQ-5B | H-κ | 3-9*01 F | 4*02 F | 1-26*01 F | 3 | CAKEDLGGYYGIYFDYW | 13 | 8 | 1-39*01 F or 1D-39*01 F | 2*01 F | CQQSYSTPYTF | 6 | 3 |
| GQ-11C | H-κ | 3-9*01 F | 4*02 F | 6-6*01 F | 1 | CAKGLEYSSSSYFNYC (TRP 118 not identified) | 2 | 1 | 1-12*01 or 02 or 1D-12*02 F | 4*01 F | CQQTNSFPLTF | 2 | 2 |
| HC-7C | H-κ | 1-58*01 F | 3*02 F | 2-2*01 F | 2 | CAAPYCSSTSCFDAFDIW | 1 | 1 | 3-20*01 F | 1*01 F | CQQYGSSPWTF | 0 | 0 |
| HC-5D | H-κ | 3-53*01 F | 5*02 F | 3-10*02 F | 2 | CARDHYGRRGGGEW | 1 | 1 | 1-33*01 F or 1D-33*01 F | 5*01 F | CQQYDNLPPTF | 0 | 0 |
| **Donor P4** | | | | | | | | | | | | | |
| GR-8E | H-κ | 3-30*03 or 18 or 3-30-5*01 F | 3*02 F | 3-10*01 F | 1 | CAKLLWFGEPWGAFDIW | 3 | 3 | 3-15*01 F | 1*01 F | CQQYNNWPGTF | 0 | 0 |
| GX-2A | H-κ | 1-69*01 F or 1-69D*01 F | 5*02 F | 6-13*01 F | 2 | CAREQQIAAVADPGTWFDPW | 0 | 0 | 1-17*03 F | 2*01 F | CLQHNSYPYTF | 0 | 0 |
| **Donor P5** | | | | | | | | | | | | | |
| GW-5D | H-𝝺 | 1-2*02 F | 6*02 F | 3-10*01 F | 1 | CARDRLLWFGENYGMDVW | 5 | 4 | 2-14*01 F or 2-14*03 F | 2*01 F or 3*01 F | CSSYTSSSSVVF | 3 | 2 |
| **Donor P6** | | | | | | | | | | | | | |
| HV-12E | H-𝝺 | 3-74*01 F | 5*02 F | 2-15*01 F | 2 | CARDISLTGVDCSGGGCYSPHLNWFDPW | 2 | 2 | 3-21*04 F | 2*01 F or 3*01 F | CQVWDSSSDVVF | 2 | 1 |

Abbreviations: H, heavy; κ, kappa; 𝝺, lambda; Vh, variable gene segment of the heavy chain variable domain; Jh, joining gene segment of the heavy chain variable domain; Dh, diversity gene segment of the heavy chain variable domain; nt Mut, number of nucleotide mutations; aa Sub, number of amino acid substitutions; Vl, variable gene segment of the light chain variable domain; Jl, joining gene segment of the light chain variable domain; rf, reading frame.
