# Supplementary material for: The presence of broadly neutralizing anti-SARS-CoV-2 RBD antibodies elicited by primary series and booster dose of COVID-19 vaccine
Source: PLoS Pathog. 2024 Jun 10;20(6):e1012246. doi: 10.1371/journal.ppat.1012246 (PMC11192315; doi:10.1371/journal.ppat.1012246)
Supplement: S4 Table — (DOCX) [file ppat.1012246.s005.docx]

**S4 Table. Non-neutralizing anti-SARS-CoV-2 RBD monoclonal antibody heavy and light chain variable domain gene usage.**

| **mAb** | **H-L** | **Vh** | **Jh** | **Dh** | **rf** | **V_h_ junction sequence** | **nt Mut** | **aa Sub** | **Vl** | **Jl** | **V_l_ Junction Sequence** | **nt Mut** | **aa Sub** |
| --- | --- | --- | --- | --- | --- | --- | --- | --- | --- | --- | --- | --- | --- |
| **Post primary series of COVID-19 vaccines** | | | | | | | | | | | | | |
| **Donor V48** | | | | | | | | | | | | | |
| IY-12A | H-κ | 4-39*01 F | 4*02 F | 5-24*01 ORF | 1 | CARRGFMATFSAW | 5 | 3 | 3-20*01 F | 1*01 F | CQQYGTSPWTF | 8 | 6 |
| IY-8B | H-κ | 3-30*04 or 3-30-3*03 F | 4*02 F | 3-3*01 F | 2 | CARDSPVFSGGYGVLDYW | 5 | 3 | 3-15*01 F | 2*01 F | CQQYHKWPPMYTF | 2 | 1 |
| IY-10C | H-κ | 3-21*01 F | 4*02 F | 2-2*01 F | 3 | CTRDKVVPDAMTLDPFDYW | 2 | 2 | 1-5*04 F | 1*01 F | CQQYYTYWTF | 1 | 1 |
| IY-9A | H-𝝺 | 7-4-1*02 F | 6*03 F | 2-8*02 F | 2 | CARESGYYSGGIYYYYYMDVW | 3 | 2 | 2-14*01 F | 1*01 F | CSSYTSSSTPYVF | 2 | 1 |
| IZ-3A | H-κ | 3-9*01 F | 5*02 F | 3-9*01 F | 1 | CAKGGTRLRFLVGWFDPW | 2 | 1 | 3-15*01 F | 5*01 F | CQQYNNWPPITF | 0 | 0 |
| IZ-4A | H-κ | 4-34*01 F | 4*02 F | 2-2*01 F | 3 | CARDIVVVPAARTPYYFDYW | 2 | 1 | 3-11*01 F | 3*01 F | CQQRSNWPTF | 0 | 0 |
| IZ-7A | H-𝝺 | 4-31*03 F | 4*02 F | 2-2*01 F | 2 | CARVIRYCSTTSCYSIDFW | 2 | 1 | 2-8*01 F | 2*01 or 3*01 F | CSSYAGSLVLF | 1 | 1 |
| IZ-3B | H-𝝺 | 1-18*01 F | 6*03 F | 2-15*01 F | 2 | CARWRVDCSGGRCHIDYYMDVW | 2 | 1 | 3-21*02 F | 2*01 or 3*01 F | CQVWDSSSDHHVVF | 1 | 1 |
| IZ-11B | H-𝝺 | 4-61*02 F | 6*02 F | 6-13*01 F | 2 | CARGIAVGGTDDYYYYYGMDVW | 5 | 3 | 3-25*03 F | 3*02 F | CQSADSSGTYWVF | 5 | 2 |
| **Donor V54** | | | | | | | | | | | | | |
| IT-1A | H-κ | 3-49*05 F | 4*02 F | 3-10*01 F | 2 | CTRVRGLSHYGSGSYADFDYW | 4 | 1 | 1-39 or 1D-39*01 F | 1*01 F | CQQSYSSPPTL | 2 | 2 |
| IT-2A | H-κ | 1-69 or 1-69D*01 F | 6*02 F | 5-12*01 F | 3 | CARAPGSSGHDYRYYYTMDVW | 4 | 4 | 3-15*01 F | 1*01 F | CQQYNNWSSF | 5 | 5 |
| IT-10A | H-κ | 1-69 or 1-69D*01 F | 6*02 F | 5-24*01 ORF | 1 | CARDVEMATIYGMDVW | 3 | 1 | 1-39 or 1D-39*01 F | 5*01 F | CQQSYSTPQTF | 0 | 0 |
| IT-1B | H-κ | 4-34*01 F | 2*01 F | 6-13*01 F | 2 | CARGRIAAADHDWYFDLW | 4 | 4 | 3-20*01 F | 2*02 F | CQQYGNSPPGFTF | 4 | 4 |
| IT-9B | H-κ | 3-33*01 or 06 F | 4*02 F | 4-17*01 F | 3 | CARDEGTTVTYFDYW | 5 | 3 | 1-39 or 1D-39*01 F | 1D-39*01 F | CQQSYSTPPWTF | 4 | 1 |
| IT-11B | H-κ | 3-30-3*01 F | 5*02 F | 5-18*01 F | 1 | CARDWGTAMVTWFDPW | 3 | 2 | 1-39 or 1D-39*01 F | 1*01 F | CQQSYSTPPWTF | 0 | 0 |
| IT-6C | H-κ | 1-46*01 or 03 F | 3*02 F | 2-2*01 F | 3 | CARGAIIPAARDAFDIW | 6 | 5 | 1-5*03 F | 1*01 F | CQQYNSYWTF | 1 | 0 |
| IT-10C | H-κ | 1-46*01 or 03 F | 4*01 or 03 F | 3-22*01 F | 3 | CARDLNPIVVISYFDYW | 9 | 7 | 3-11*01 F | 5*01 F | CQHRRNWPPAITF | 1 | 1 |
| IT-12C | H-κ | 3-48*02 F | 6*02 F | 3-10*01 F | 1 | CARHLGELSYYYYGMDVW | 3 | 2 | 1-39 or 1D-39*01 F | 5*01 F | CQQSYSTPPTF | 2 | 1 |
| IT-3A | H-𝝺 | 3-9*01 F | 6*02 F | 3-16*01 F | 1 | CAKDYRIGDERLGSYGMDVW | 2 | 2 | 3-21*04 F | 1*01 F | CQVWDSSSDHYVF | 0 | 0 |
| IT-9A | H-𝝺 | 4-34*01 F | 4*02 F | 2-2*01 F | 3 | CARGATMPYDYW | 3 | 2 | 2-14*03 F | 1*01 F | CSSYTSSSTLYVF | 0 | 0 |
| IT-2B | H-𝝺 | 3-11*01 F | 4*02 F | 5-12*01 F | 2 | CARAQWLRGHFDYW | 4 | 3 | 6-57*01 F | 3*02 F | CQSYFGSNLWVF | 2 | 2 |
| IT-5B | H-𝝺 | 4-39*01 F | 5*02 F | 6-25*01 F | 2 | CARPHPIAAATTGWFDPW | 7 | 4 | 3-21*03 F | 1*01 F | CQVWDTSSNPPSYVF | 1 | 0 |
| IT-7B | H-𝝺 | 4-39*01 F | 4*02 F | 3-22*01 F | 2 | CASLEFYDTWYFDYW | 2 | 2 | 2-14*01 or 03 F | 2*01 or 3*01 F | CSSYTSSSTSVVF | 4 | 4 |
| IT-1C | H-𝝺 | 4-31*03 F | 6*02 F | 3-10*01 F | 2 | CARAPSFYGSGSYYKYYYGMDVW | 0 | 0 | 3-21*03 F | 2*01 or 3*01 F | CQLWDSSFDHVVF | 5 | 3 |
| IT-4C | H-𝝺 | 3-7*01 F | 3*02 F | 5-24*01 ORF | 2 | CAGLLWLQGAFDIW | 4 | 2 | 6-57*04 F | 3*02 F | CQSYDSTNWVF | 1 | 1 |
| JH-6A | H-𝝺 | 3-9*01 F | 3*02 F | 3-10*01 F | 2 | CAKDFGVGESYASGTWGFDIW | 5 | 4 | 3-21*03 F | 3*02 F | CQVWISSSDHWVF | 3 | 2 |
| JH-7C | H-𝝺 | 5-51*01 F | 6*02 F | 3-10*01 F | 2 | CARHSDYYGSGGMDVW | 3 | 3 | 1-44*01 F | 3*02 F | CAAWDDSLNGWVF | 2 | 2 |
| JH-8C | H-𝝺 | 4-31*03 F | 5*02 F | 1-26*01 F | 1 | CARDLGATAYNWFDPW | 0 | 0 | 1-51*01 F | 1*01 F | CGTWDGSLSPGLYVF | 4 | 3 |
| JH-4D | H-𝝺 | 5-51*01 F | 6*02 F | 3-16*01 F | 2 | CARPLGAIFSGMDVW | 0 | 0 | 1-44*01 F | 1*01 F | CAAWDDSLNGYVF | 4 | 4 |
| JH-9B | H-κ | 1-46*01 or 03 F | 3*02 F | 2-2*01 F | 3 | CARGGIAPSQDAFDIW | 8 | 6 | 1-5*03 F | 1*01 F | CQHYKSYSTF | 0 | 0 |
| JH-4C | H-κ | 1-46*01 or 03 F | 4*02 F | 3-3*01 F | 3 | CARGTIPPHQGPFDFW | 4 | 4 | 1-5*03 F | 1*01 F | CQHYNGYSLWTF | 2 | 2 |
| **Donor V55** | | | | | | | | | | | | | |
| IV-11A | H-κ | 3-64D*06 F | 4*02 F | 5-18*01 F | 1 | CVKDVDTAMVTVFDYW | 3 | 3 | 1-39 or 1D-39*01 F | 2*01 F | CQQSYSTPPTF | 1 | 0 |
| IV-12B | H-κ | 3-33*01 or 06 F | 3*02 F | 2-2*01 F | 3 | CVKDLSIVSATIHAFDI* (TRP 118 not identified) | 1 | 1 | 1-8*01 F | 2*01 F | CQQYYTYPQNTF | 4 | 3 |
| IV-5C | H-κ | 3-13*01 F | 2*01 F | 5-18*01 F | 3 | CARGGWGYSYGSWYFDLW | 0 | 0 | 1-39 or 1D-39*01 F | 5*01 F | CQQSYSNPSITF | 1 | 1 |
| IV-7C | H-κ | 5-10-1*03 F | 4*02 F | 3-22*01 F | 2 | CARGRSYYDSRGRFDYW | 3 | 2 | 1-33 or 1D-33*01 F | 3*01 F | CQQYDSLGFTF | 2 | 2 |
| IV-1D | H-κ | 3-64D*06 F | 3*02 F | 1-26*01 F | 3 | CVKDFGSNFWDAFDIW | 5 | 5 | 1-39 or 1D-39*01 F | 2*02 F | CQQSYSTPRTF | 0 | 0 |
| IV-1E | H-κ | 3-30*04 or 3-30-3*03 F | 3*02 F | 6-25*01 F | 2 | CARDFIAAATGGIAFDIW | 1 | 1 | 1-39 or 1D-39*01 F | 4*01 F | CQQSYSTLALTF | 0 | 0 |
| IV-2E | H-κ | 3-30*04 or 3-30-3*03 F | 4*02 F | 6-13*01 F | 3 | CARDTEQQLVPQFDYW | 1 | 1 | 1-39 or 1D-39*01 F | 4*01 F | CQQSYSTLALTF | 1 | 1 |
| IV-4E | H-κ | 3-15*01 F | 1*01 F | 3-22*01 F | 2 | CTTDRLTIDSSGYYYRHW | 0 | 0 | 4-1*01 F | 1*01 F | CQQYYTAPRTF | 1 | 1 |
| IV-3A | H-𝝺 | 4-39*07 F | 3*02 F | 3-9*01 F | 2 | CARERVTDILTGYTYAPGAFDIW | 5 | 5 | 3-1*01 F | 2*01 F | CQAWDSSTVVF | 1 | 0 |
| IV-6A | H-𝝺 | 3-9*01 F | 3*02 F | 1-26*01 F | 2 | CAKDMLGEWELGNAFDIW | 0 | 0 | 1-51*01 F | 2*01 F | CGTWDSSLSA | 1 | 1 |
| IV-10B | H-𝝺 | 4-34*01 F | 4*02 F | 5-24*01 ORF | 1 | CAREEMATVFDYW | 0 | 0 | 2-11*01 F | 2*01 F | CCSYAGGYTYVVF | 1 | 1 |
| IV-8D | H-𝝺 | 3-23 or 3-23D*01 F | 4*02 F | 1-20*01 F | 3 | CAKDGAYNWNDVFDYW | 6 | 3 | 2-14*03 F | 2*01 F | CSSYASHLVF | 0 | 0 |
| IV-10E | H-𝝺 | 4-59*01 F | 4*02 F | 5-12*01 F | 3 | CAATLGVDYYCDYW | 6 | 4 | 2-14*01 or 03 F | 2*01 or 3*01 F | CSSYTSSSTLVVF | 2 | 2 |
| IS-3A | H-κ | 3-33*01 or 06 F | 4*02 F | 2-15*01 F | 2 | CAKQLGAYCSGGSCYAGFDYW | 5 | 4 | 1-33 or 1D-33*01 F | 3*01 F | CQQYDNLPLAF | 1 | 1 |
| IS-6A | H-κ | 3-30*04 or 3-30-3*03 F | 4*02 F | 3-22*01 F | 3 | CARDRDMIVVVIDYW | 5 | 5 | 1-39 or 1D-39*01 F | 5*01 F | CQQSYSTPPITF | 1 | 0 |
| IS-11A | H-κ | 3-64D*06 F | 4*02 F | 5-18*01 F | 1 | CVKDTDTAMVTILEYW | 6 | 5 | 1-39 or 1D-39*01 F | 4*01 F | CQQSYSTPLTF | 0 | 0 |
| IS-5B | H-κ | 3-64D*06 F | 3*02 F | 1-26*01 F | 3 | CVKDFGSNFWDAFDIW | 5 | 5 | 1-39 or 1D-39*01 F | 2*02 F | CQQSYSTPRTF | 1 | 0 |
| **Donor V59** | | | | | | | | | | | | | |
| IW-3A | H-κ | 4-39*02 F | 1*01 F | 6-13*01 F | 2 | CASLGGSLGDRIAAAGTVHYW | 4 | 4 | 1-33 or 1D-33*01 F | 4*01 F | CQQFDNLPLTF | 1 | 1 |
| IW-6A | H-κ | 1-18*01 F | 6*02 F | 3-10*03 F | 2 | CAREGSYSGYYYGMDVW | 3 | 1 | 3-11*01 F | 2*01 F | CQQRINWPPYAF | 1 | 1 |
| IW-8A | H-κ | 3-30*03 or 18 or 3-30-5*01 F | 4*02 F | 2-2*01 F | 2 | CAKKGGPYCSGTSCYAGIIDYW | 5 | 5 | 1-33 or 1D-33*01 F | 3*01 F | CQQYDNLPFTF | 1 | 1 |
| IW-11A | H-κ | 3-13*01 F | 5*02 F | 3-10*01 F | 2 | CVRGLDFYGSGNFYFYNWFDPW | 4 | 2 | 1-39 or 1D-39*01 F | 2*01 F | CQQSYITTHYTF | 2 | 1 |
| IW-3B | H-κ | 3-13*01 F | 6*03 F | 3-16*02 F | 1 | CARSESSSGLSYYYYYYMDVW | 6 | 4 | 1-39 or 1D-39*01 F | 2*01 F | CQQSYSTTEYTF | 5 | 1 |
| IW-8B | H-κ | 4-61*02 F | 4*02 F | 3-22*01 F | 2 | CARGIIYYFGNSDCW | 36 | 25 | 1-39 or 1D-39*01 F | 2*01 F | CQQSYSTTYTF | 2 | 2 |
| IW-11B | H-κ | 3-13*01 F | 6*03 F | 6-13*01 F | 1 | CARADSSWNGPHYYYYYMDVW | 3 | 2 | 3-15*01 F | 4*01 F | CQQYHNWPPLTF | 3 | 2 |
| IW-2C | H-κ | 3-13*01 F | 2*01 F | 4-17*01 F | 2 | CASGLGDYLWYFDLW | 2 | 1 | 1-39 or 1D-39*01 F | 4*01 F | CQESYGIPPVTF | 3 | 0 |
| IW-5B | H-𝝺 | 4-31*03 F | 6*03 F | 3-16*02 F | 1 | CASSFLQDYYMDVW | 8 | 2 | 2-14*03 F | 1*01 F | CSSYTTNTPSVF | 4 | 4 |
| **Donor V74** | | | | | | | | | | | | | |
| IX-1A | H-κ | 3-43*02 F | 4*02 F | 3-3*01 F | 3 | CAKSHTIFGVVSPGYW | 2 | 0 | 2D-29*02 F | 1*01 or 3*01 F | CMQSIQLRGTF | 2 | 2 |
| IX-5A | H-κ | 1-69*09 F | 6*03 F | 5-12*01 F | 1 | CARGGPLSDIVATIDYYYMDVW | 2 | 1 | 3-15*01 F | 1*01 F | CHQYNNWPSWTF | 3 | 0 |
| IX-8A | H-κ | 4-4*02 F | 4*02 F | 6-25*01 F | 3 | CARRPLFDYW | 0 | 0 | 1-NL1*01 F | 2*01 F | CHQYYSIPTF | 3 | 3 |
| IX-2B | H-κ | 3-13*01 F | 6*03 F | 1-26*01 F | 1 | CARGTTTYYYYYYMDVW | 4 | 4 | 1-39 or 1D-39*01 F | 2*01 F | CQQSYSLPGYTF | 4 | 3 |
| IX-7C | H-κ | 1-46*01 or 03 F | 4*02 F | 2-2*01 F | 3 | CARDLGLLPAAAAFDYW | 17 | 13 | 1-5*04 F | 1*01 F | CQQYNSYSRTF | 1 | 0 |
| IX-9C | H-κ | 3-13*01 F | 6*03 F | 5-18*01 F | 2 | CARGIQLWFKGHYYYYMDVW | 5 | 4 | 1-39 or 1D-39*01 F | 2*01 F | CQQSYSSPMYTF | 2 | 1 |
| IX-9A | H-𝝺 | 1-46*01 or 03 F | 3*02 F | 2-2*01 F | 2 | CARDRGYCSSTSCYPHDAFDIW | 5 | 3 | 3-21*02 F | 1*01 F | CQVWDSSSDPYVF | 0 | 0 |
| IX-12A | H-𝝺 | 3-7*03 F | 4*02 F | 2-15*01 F | 2 | CARDEGPEYCSGGRCYSEYIDYW | 4 | 1 | 3-25*03 F | 2*01 or 3*01 F | CQSGDSSGLVF | 8 | 5 |
| IX-1B | H-𝝺 | 3-9*01 F | 6*03 F | 3-3*01 F | 2 | CAKLESVGYYDFWSGRGDYMDVW | 7 | 5 | 1-40*01 or 02 F | 2*01 or 3*01 F | CQSYDSSLSGVVF | 3 | 3 |
| IX-8B | H-𝝺 | 3-9*01 F | 3*02 F | 6-13*01 F | 1 | CAKLTSSSWYGAFDIW | 1 | 1 | 3-21*02 F | 2*01 or 3*01 F | CQVWDSSTDPPF | 6 | 3 |
| IX-1C | H-𝝺 | 3-9*01 F | 3*02 F | 3-3*01 F | 3 | CAKDGTTFRVAMDDSYAFDIW | 4 | 3 | 2-14*01 or 03 F | 2*01 or 3*01 F | CSSYTRSSTVVF | 6 | 4 |
| IX-6C | H-𝝺 | 1-18*01 F | 4*02 F | 1-26*01 F | 3 | CARVPYSGSSFLYYFDYW | 0 | 0 | 3-21*02 F | 3*02 F | CQVWDSSSDHFWVF | 3 | 2 |
| IX-8C | H-𝝺 | 3-30*03 or 18 or 3-30-5*01 F | 4*02 F | 2-2*01 F | 2 | CAKDATYCSSTRCYPGDYFDYW | 2 | 1 | 3-21*04 F | 3*02 F | CQAWDSSGDHWVF | 2 | 2 |
| IX-10C | H-𝝺 | 3-9*01 F | 3*02 F | 3-22*01 F | 2 | CAKGYDSSGYHTLHDAFDIW | 11 | 9 | 3-21*02 F | 2*01 or 3*01 F | CQVWDSSSHVVF | 1 | 0 |
| IX-11C | H-𝝺 | 4-30-4*01 F | 3*02 F | 3-22*01 F | 2 | CARLPRGYYDSSGYYHHAFDIW | 2 | 1 | 2-23*01 or 03 F | 2*01 or 3*01 F | CCSYVGSSTFVVF | 0 | 0 |
| **Donor V75** | | | | | | | | | | | | | |
| JE-3A | H-κ | 3-9*01 F | 4*02 F | 3-10*01 F | 3 | CVKDIEKFGVRGVTGGFDYW | 6 | 6 | 4-1*01 F | 3*01 F | CQQYYSSPFTF | 8 | 7 |
| JE-11A | H-κ | 3-30*20 or 3-30-3*01 F | 4*02 F | 5-18*01 F | 1 | CARDVPVETAMVPVLGYW | 7 | 6 | 1-39 or 1D-39*01 F | 5*01 F | CQQSYSTPSITF | 1 | 0 |
| JE-8B | H-κ | 3-64D*06 F | 4*02 F | 4-17*01 F | 3 | CVTEKMTTVTTVFDYW | 5 | 4 | 1-39 or 1D-39*01 F | 1*01 F | CQQSYTTPTF | 1 | 1 |
| JE-12B | H-κ | 3-30*04 or 3-30-3*03 F | 4*02 F | 3-22*01 F | 3 | CARDQGVVTTYFDYW | 5 | 4 | 1-39 or 1D-39*01 F | 1*01 F | CQQSYNTPPWTF | 5 | 4 |
| JE-2C | H-κ | 1-46*01 or 03 F | 6*02 F | 3-10*02 F | 2 | CARDTYYIPASGAMDVW | 7 | 6 | 1-39 or 1D-39*01 F | 2*01 F | CQQSYSTPPMYTF | 3 | 3 |
| JE-6C | H-κ | 3-30*03 or 18 or 3-30-5*01 F | 4*02 F | 4-17*01 F | 2 | CAKGGMYGDHVRIDYW | 4 | 3 | 1-33 or 1D-33*01 F | 3*01 F | CQHYNNLPLTF | 4 | 3 |
| JE-12C | H-κ | 3-30*03 or 18 or 3-30-5*01 F | 4*02 F | 3-22*01 F | 2 | CAKPLQNGDRSALSLGYFDYW | 6 | 5 | 3-11*01 F | 2*01 or 03 F | CQQLSNWPYIF | 1 | 1 |
| JE-1A | H-𝝺 | 3-9*01 F | 6*02 F | 5-18*01 F |  | CAKDISVDTAMGGRGWDYGMDVW | 4 | 3 | 3-21*03 F | 2*01 or 3*01 F | CQVWDSSSDRRVF | 0 | 0 |
| JE-8A | H-𝝺 | 3-33*01 or 06 F | 4*02 F | 6-13*01 F | 1 | CAKDPSMYSSSWYIGVDYW | 8 | 5 | 6-57*01 F | 3*02 F | CQSYDSSNHWVF | 3 | 1 |
| JE-9A | H-𝝺 | 4-31*03 F | 3*02 F | 3-22*01 F | 2 | CARFTRSYYDSSGFYPHAFDIW | 2 | 2 | 2-23*01 or 03 F | 3*02 F | CCSYAGSSTTLF | 2 | 0 |
| JE-10A | H-𝝺 | 3-33*01 or 06 F | 6*02 F | 3-9*01 F | 2 | CARGGPRTYSDILTGHEDYYYYVMDVW | 7 | 5 | 2-14*03 F | 3*02 F | CSSYTSSSTWVF | 0 | 0 |
| JE-12A | H-𝝺 | 3-30-3*01 F | 3*02 F | 3-10*01 F | 2 | CARGDYYGSGSYYNPLPVFDIW | 6 | 5 | 1-44*01 F | 3*02 F | CAAWDDSLNSWVF | 2 | 2 |
| JE-10C | H-𝝺 | 3-9*01 F | 4*02 F | 6-19*01 F | 1 | CAKDYGSGWYGVLDYW | 8 | 6 | 3-21*03 F | 1*01 F | CQVWDSSTDHLYVF | 4 | 2 |
| **Donor V76** | | | | | | | | | | | | | |
| JF-4A | H-κ | 3-30-3*01 F | 4*02 F | 5-18*01 F | 1 | CARDVPPMASMVPIFDYW | 9 | 5 | 1-39 or 1D-39*01 F | 3*01 F | CQQSYSTLGLTF | 1 | 0 |
| JF-12A | H-κ | 1-69 or 1-69D*01 F | 6*02 F | 5-12*01 F | 1 | CARDSVTIGPLKSYYYYGMDVW | 0 | 0 | 1-33 or 1D-33*01 F | 4*01 F | CQQYDNLPLTF | 0 | 0 |
| JF-4B | H-κ | 3-13*01 F | 2*01 F | 6-13*01 F | 3 | CARVGEQQLALGDWYFDLW | 4 | 4 | 1-39 or 1D-39*01 F | 5*01 F | CQQSYSMPPITF | 2 | 1 |
| JF-8B | H-κ | 3-13*01 F | 3*02 F | 3-22*01 F | 2 | CARAHYDSSGYYSAFDIW | 1 | 1 | 1-39 or 1D-39*01 F | 2*01 F | CQQSYSTLSYTF | 0 | 0 |
| JF-3C | H-κ | 3-9*01 F | 4*02 F | 6-19*01 F | 2 | CAKDLGDVIAVGGTISGVWGFDFW | 8 | 4 | 1-39 or 1D-39*01 F | 2*01 F | CQQSYSTPPYTF | 1 | 1 |
| JF-1A | H-𝝺 | 3-9*04 F | 4*02 F | 3-22*01 F | 2 | CAKEIAMRDYYDSSSNFDYW | 5 | 3 | 1-51*01 F | 2*01 or 3*01 F | CGTWDSSLSTVVF | 9 | 3 |
| JF-7A | H-𝝺 | 4-39*01 F | 4*02 F | 4-23*01 ORF | 3 | CARHTRVADFDYW | 10 | 5 | 6-57*02 F | 3*02 F | CQSYDSSNHWVF | 2 | 2 |
| JF-7C | H-𝝺 | 3-30-3*01 F | 6*02 F | 4-17*01 F | 2 | CARDTGLRGYGDNVHSPNWSGTYYYDYYGMDVW | 6 | 4 | 1-36*01 F | 3*02 F | CAAWDDSLNGWVF | 5 | 3 |
| JF-12C | H-𝝺 | 4-30-4*01 F | 3*02 F | 3-22*01 F | 2 | CARVPRSYYDSSGYYPYTFDIW | 0 | 0 | 2-23*02 F | 3*02 F | CCSYVGSSTWLF | 2 | 1 |
| JF-3D | H-𝝺 | 3-30-3*01 F | 4*02 F | 3-10*01 F | 2 | CARGDYYGSGRYYNPLPLFDYW | 8 | 6 | 1-44*01 F | 3*02 F | CAAWDDSLNGWVF | 4 | 2 |
| JF-4D | H-𝝺 | 3-48*02 F | 4*02 F | 3-10*01 F | 2 | CARDYNYYGSGSYDYW | 0 | 0 | 2-8*01 F | 3*02 F | CSSYAGRNNLVF | 2 | 1 |
| JF-7D | H-𝝺 | 4-34*01 F | 4*02 F | 6-19*01 F | 1 | CARGPSSGWYGRGFDYW | 2 | 1 | 1-47*01 F | 3*02 F | CAAWDDSLSVWVF | 9 | 4 |
| JF-3E | H-𝝺 | 1-18*01 F | 4*02 F | 2-15*01 F | 3 | CARRMMVTADFDYW | 10 | 8 | 6-57*02 F | 2*01 or 3*01 F | CQSYDSSNVVF | 1 | 1 |
| JF-5E | H-𝝺 | 3-66*01 or 04 F | 5*02 F | 5-12*01 F | 2 | CAPLEWLRGWFDPW | 2 | 1 | 6-57*02 F | 3*02 F | CQSYDSSNHWVF | 0 | 0 |
| **Donor 84** | | | | | | | | | | | | | |
| JG-8A | H-κ | 1-69 or 1-69D*01 F | 6*02 F | 5-12*01 F | 3 | CARDSAYGPHAGMDVW | 2 | 1 | 1-39 or 1D-39*01 F | 2*01 F | CQQSYSTPNTF | 2 | 0 |
| JG-11A | H-κ | 3-30*04 or 3-30-3*03 F | 4*02 F | 5-18*01 F | 3 | CAREPRAYSYGSSYLDYW | 5 | 5 | 1-5*01 F | 1*01 F | CQQYNSYSTF | 2 | 2 |
| JG-2B | H-κ | 3-13*01 F | 3*02 F | 1-7*01 F | 1 | CARGYITGITWAFDIW | 7 | 4 | 1-39 or 1D-39*01 F | 4*01 F | CQQSYSNPPATF | 6 | 6 |
| JG-5B | H-κ | 3-23*04 F | 4*02 F | 2-15*01 F | 2 | CAKDQRGGPGGSCYDYW | 10 | 7 | 1-5*01 F | 1*01 F | CQQYNTYSGTF | 4 | 3 |
| JG-7B | H-κ | 5-51*01 F | 4*02 F | 2-21*02 F | 2 | CARQFCGGDCPLDYW | 4 | 2 | 1-5*01 F | 1*01 F | CQQYNSYWTF | 0 | 0 |
| JG-9B | H-κ | 3-64D*09 F | 4*02 F | 1-20*01 F | 1 | CVKDLMTGTVDIPDYW | 6 | 5 | 1-39 or 1D-39*01 F | 1*01 F | CHQSYSTPWTF | 0 | 0 |
| JG-12B | H-κ | 3-64D*09 F | 4*02 F | 5-18*01 F | 1 | CVKDVDTAMVTIFDYW | 4 | 3 | 1-39 or 1D-39*01 F | 2*01 F | CQQSYTTPYTF | 1 | 1 |
| JG-1C | H-κ | 3-23*04 F | 4*02 F | 3-10*02 F | 1 | CVKDLYAGVGPQLGYFW | 4 | 4 | 1-5*01 F | 1*01 F | CQQYNSYSWTF | 0 | 0 |
| JG-2C | H-κ | 3-64D*09 F | 4*02 F | 1-26*01 F | 2 | CVKDSSLRWELLLDYW | 5 | 5 | 1-39 or 1D-39*01 F | 2*01 F | CQQSYTTPYTF | 0 | 0 |
| JG-6C | H-κ | 3-30*04 or 3-30-3*01 or 03 F | 4*02 F | 6-13*01 F | 1 | CARDSYSSSWPIFDYW | 8 | 6 | 1-39 or 1D-39*01 F | 4*01 F | CQQSYSTLALTF | 2 | 1 |
| JG-7C | H-κ | 3-13*04 F | 2*01 F | 6-19*01 F | 1 | CARGAASDWDWYFDLW | 1 | 1 | 1-39 or 1D-39*01 F | 4*01 F | CQQSYSNPRPTF | 0 | 0 |
| JG-10E | H-κ | 4-39*01 F | 4*02 F | 4-23*01 ORF | 2 | CARDYGGNSNYFGYW | 4 | 2 | 1-5*01 F | 4*01 F | CQQYNSYPLTF | 2 | 2 |
| JG-12E | H-κ | 3-13*04 F | 4*02 F | 3-9*01 F | 2 | CARVNYDILAGYYDYW | 5 | 2 | 1-39 or 1D-39*01 F | 1*01 F | CQQSYISPPWTF | 2 | 0 |
| JG-3B | H-𝝺 | 4-61*02 F | 4*02 F | 5-12*01 F | 3 | CASGYDFRYFDYW | 2 | 2 | 6-57*03 F | 2*01 or 3*01 F | CQSYHSSNVVF | 2 | 2 |
| JG-8B | H-𝝺 | 5-10-1*04 F | 4*02 F | 3-10*01 F | 2 | CARGGVHYGSGSYDDYW | 5 | 2 | 7-43*01 F | 1*01 F | CLLYYGGAQVF | 0 | 0 |
| JG-8E | H-𝝺 | 3-30*04 or 15 or 3-30-3*03 F | 4*02 F | 3-10*01 F | 2 | CARESGYGSGTYQASPFDSW | 5 | 5 | 3-21*04 F | 2*01 or 3*01 F | CQVWDIFVVF | 2 | 2 |
| **Donor 57** | | | | | | | | | | | | | |
| JD-4B | H-κ | 3-30*03 or 18 or 3-30-5*01 F | 4*02 F | 2-21*02 F | 2 | CAKRGGAYCGGDCYTSFFDYW | 2 | 2 | 1-33 or 1D-33*01 F | 4*01 F | CQQYDNLPLTF | 1 | 1 |
| **Donor 59** | | | | | | | | | | | | | |
| JD-2A | H-κ | 4-59*08 F | 5*02 F | 2-2*01 F | 3 | CASHLPGIPWGWFDPW | 11 | 9 | 1-39 or 1D-39*01 F | 2*01 F | CQQSYSTVYTF | 1 | 1 |
| JD-4A | H-κ | 3-33*01 or 06 or 08 F | 4*02 F | 2-15*01 F | 3 | CARDEEIVVADPDYW | 15 | 8 | 2-30*01 F | 1*01 F | CMQGTHWPWTF | 4 | 4 |
| JD-1A | H-𝝺 | 3-23 or 3-23D*01 F | 4*02 F | 6-6*01 F | 1 | CAKGTLLVDEYDSLDDGFDYW | 7 | 6 | 3-27*01 F | 3*02 F | CYSAADNDRVF | 2 | 1 |
| JD-2A | H-𝝺 | 4-59*08 F | 5*02 F | 2-2*01 F | 3 | CASHLPGIPWGWFDPW | 11 | 9 | 3-21*04 F | 2*01 or 3*01 F | CQVWDTGSDHVIF | 0 | 0 |
| JD-6A | H-𝝺 | 5-51*01 F | 5*01 F 5*02 F | 2-15*01 F | 2 | CVRHSGGYCSNGSCYDGPGDYW | 4 | 4 | 3-21*04 F | 2*01 or 3*01 F | CQVWDRSSDHLNVIF | 7 | 6 |
| **Donor 60** | | | | | | | | | | | | | |
| JC-1A | H-κ | 1-2*02 F | 4*02 F | 6-13*01 F | 1 | CARDRSSSWYEDFDYW | 6 | 5 | 1-39 or 1D-39*01 F | 1*01 F | CQQSYSTPPVTF | 5 | 3 |
| JC-3A | H-κ | 3-30*03 or 18 or 3-30-5*01 F | 4*02 F | 5-12*01 F | 1 | CAKDCPDTMASFPDYW | 10 | 9 | 4-1*01 F | 1*01 F | CQQYYGTPWTF | 5 | 5 |
| JC-4B | H-κ | 3-13*01 F | 4*02 F | 6-19*01 F | 1 | CARVSYSGWYMFFDHW | 5 | 3 | 1-39 or 1D-39*01 F | 2*01 F | CQQSYVTTMYTF | 3 | 2 |
| JC-1C | H-κ | 1-46*01 or 03 F | 4*02 F | 2-15*01 F | 2 | CARDWAFLPASGGSDYW | 13 | 6 | 1-39 or 1D-39*01 F | 1*01 F | CQESYSSHWTF | 2 | 2 |
| JC-8C | H-κ | 3-30*03 or 18 or 3-30-5*01 F | 4*02 F | 2-15*01 F | 2 | CAKLGGEYCGGGNCYSGYFDYW | 13 | 10 | 1-39 or 1D-39*01 F | 5*01 F | CQQYDNLPITF | 2 | 2 |
| JC-10C | H-κ | 3-30*03 or 18 or 3-30-5*01 F | 4*02 F | 3-22*01 F | 2 | CAKTQGEYYYDSSGHFLLFDYW | 5 | 3 | 1-39 or 1D-39*01 F | 5*01 F | CQHSFTF | 3 | 3 |
| JC-12C | H-κ | 3-30*03 or 18 or 3-30-5*01 F | 4*02 F | 2-15*01 F | 2 | CAKQAEPYCSGGSCYSSYFDYW | 9 | 5 | 1-5*03 F | 1*01 F | CQQYNSYSSF | 11 | 3 |
| **Donor 74** | | | | | | | | | | | | | |
| JO-2A | H-κ | 3-33*01 or 06 F | 4*02 F | 2-15*01 F | 2 | CAKALGPYCSGGSCYSAAFDYW | 1 | 0 | 3-20*01 F | 5*01 F | CQQYGSSPPITF | 1 | 1 |
| JO-5A | H-κ | 3-43D*03F | 6*02 F | 3-9*01 F | 2 | CAKDNRAHYDILTGYEGMDVW | 1 | 1 | 2-28 or 2D-28*01 F | 4*01 F | CMQGLQTPLTF | 2 | 2 |
| JO-9A | H-κ | 5-51*01 F | 6*02 F | 4-23*01 ORF | 2 | CARGGSSPPPHYYYYYGMDVW | 3 | 3 | 3-15*01 F | 1*01 F | CQQYNNWPWTF | 2 | 2 |
| JO-2B | H-κ | 3-13*01 F | 4*02 F | 5-18*02 ORF | 3 | CARGKDSGYHYYFDYW | 9 | 7 | 1-39 or 1D-39*01 F | 1*01 F | CQQSYSSPPWTF | 4 | 4 |
| JO-10B | H-κ | 1-69 or 1-69D*01 F | 4*02 F | 2-15*01 F | 2 | CARGGGGIYCSGGSCYRYYFDYW | 3 | 2 | 3-20*01 F | 1*01 F | CQQYGSPVTF | 1 | 1 |
| JO-3C | H-κ | 3-30*03 or 18 or 3-30-5*01 F | 4*02 F | 2-21*02 F | 2 | CAKKFGPYCGGDCYSYYFDYW | 4 | 3 | 4-1*01 F | 2*02 F | CQQYYTTPSSTF | 3 | 3 |
| JO-4C | H-κ | 3-9*01 F | 6*02 F | 5-12*01 F | 3 | CAKSMGYSDYDFGQQQLGGYYYYAVDVW | 2 | 2 | 1-33 or 1D-33*01 F | 1*01 or 2*02 F | CQQYDNLPRTF | 1 | 0 |
| JO-6C | H-κ | 3-13*01 F | 6*02 F | 3-10*01 F | 2 | CARALYGSGSFYKGGGYYYGMDVW | 9 | 6 | 3-15*01 F | 4*01 F | CQQYNNWPPLTF | 4 | 3 |
| JO-1C | H-𝝺 | 4-31*03 F | 4*02 F | 1-26*01 F | 1 | CARFIVGANTFSDYW | 10 | 6 | 3-21*04 F | 2*01 or 3*01 F | CQVWDSSSDHPVF | 1 | 1 |
| **Post booster dose of COVID-19 vaccines** | | | | | | | | | | | | | |
| **Donor V48** | | | | | | | | | | | | | |
| JN-7A | H-κ | 3-13*01 F | 6*03 F | 6-6*01 F | 3 | CARADGQPLRSYYMDVW | 6 | 5 | 1-39 or 1D-39*01 F | 1*01 F | CQESYSNPPWTF | 5 | 5 |
| JN-9A | H-κ | 4-4*02 F | 4*02 F | 6-19*01 F | 1 | CARSSGFFDYW | 12 | 8 | 1-NL1*01 F | 1*01 F | CQQYYSTRSF | 12 | 6 |
| JN-12A | H-κ | 1-46*01 or 02 or 03 F | 3*02 F | 2-2*01 F | 3 | CARGGIVPSASDAFDIW | 15 | 10 | 3-20*01 F | 4*01 F | CQQYDSSPFTF | 5 | 4 |
| JN-8B | H-κ | 1-46*01 or 03 F | 4*02 F | 6-13*01 F | 1 | CARAWSLIPSSSSPLEYW | 17 | 12 | 3-20*01 F | 4*01 F | CQQYDSSPFTF | 5 | 4 |
| JN-11B | H-κ | 1-46*01 or 02 or 03 F | 6*03 F | 3-10*01 F | 3 | CAREGTLIPSKYHYMDVW | 24 | 14 | 3-11*01 F | 5*01 F | CQQSSNWAITF | 6 | 5 |
| JN-5C | H-κ | 3-13*01 F | 2*01 F | 4-17*01 F | 2 | CARVDYDDNGLNWHLDLW | 12 | 10 | 1-39 or 1D-39*01 F | 2*01 F | CQQSYSNPLYTF | 13 | 8 |
| JN-7C | H-κ | 3-13*01 F | 3*02 F | 4-23*01 ORF | 2 | CARASFGGHRGLVSAFDIW | 17 | 9 | 1-39 or 1D-39*01 F | 1*01 F | CQQSYSIPPWTF | 8 | 7 |
| JN-10A | H-𝝺 | 4-39*01 F | 4*02 F | 5-18*01 F | 3 | CARLLRGYSYGYSFDYW | 2 | 2 | 2-23*01 or 03 F | 2*01 or 3*01 F | CFSYAGSSTLLF | 2 | 1 |
| JN-3C | H-𝝺 | 4-39*01 F | 4*02 F | 2-2*02 F | 2 | CARLWRYCGSTSCYNFDYW | 5 | 3 | 2-23*01 or 02 or 03 F | 3*02 F | CCSYAGSSTLLF | 5 | 4 |
| **Donor V107** | | | | | | | | | | | | | |
| JM-5A | H-κ | 3-13*01 F | 6*03 F | 1-26*01 F | 1 | CTRGSTTSYFYYMDVW | 12 | 9 | 1-39 or 1D-39*01 F | 3*01 F | CQQSYIMPPWTF | 12 | 4 |
| JM-4B | H-κ | 4-34*01 F | 3*02 F | 3-22*01 F | 2 | CATDISAPNDVFDIW | 7 | 6 | 3D-20*01 F | 4*01 or 02 F | CQQYGTSPLTF | 11 | 8 |
| JM-6B | H-κ | 4-39*07 F | 3*02 F | 6-19*01 F | 1 | CARGVGAYSSGWDGLEAFEIW | 11 | 3 | 3-20*01 F | 5*01 F | CQHYYSSPITF | 3 | 1 |
| JM-3C | H-κ | 3-13*01 F | 4*02 F | 6-13*01 F | 1 | CARRSNSRLHYYFDYW | 18 | 15 | 1-39 or 1D-39*01 F | 1*01 F | CQQSYSSPPWTF | 9 | 2 |
| JM-4C | H-κ | 1-60*01 or 1-69*18 or 1-69D*01 F | 6*02 F | 3-10*02 F | 1 | CARDQVLGPVYGMDVW | 17 | 10 | 1-39 or 1D-39*01 F | 4*01 F | CQQSYRVPPLTF | 6 | 3 |
| JM-8C | H-κ | 3-30*03 or 18 or 3-30-5*01 F | 5*02 F | 3-16*02 F | 2 | CARDFSGSYLVTWFDPW | 19 | 9 | 1-39 or 1D-39*01 F | 1*01 F | CQQSYNTPPWTF | 12 | 7 |
| JM-4D | H-κ | 3-30*03 or 18 F or 3-30-3*01 F | 4*02 F | 5-18*01 F | 3 | CAKGGYSYAYYYGGYLDYW | 13 | 8 | 1-33 or 1D-33*01 F | 1*01 F | CQHYDNLPPWTF | 9 | 6 |
| JM-5D | H-κ | 3-21*01 F | 4*02 F | 6-13*01 F | 2 | CARTDPRTRINAVGTVDYW | 4 | 3 | 1-NL1*01 F | 1*01 F | CQQYYSSPQTF | 6 | 3 |
| JM-10D | H-κ | 1-18*01 F | 4*02 F | 2-15*01 F | 2 | CARVGNGYCSGGSCYFFDYW | 12 | 7 | 3-15*01 F | 1*01 F | CQQYDNWPPTWTF | 7 | 5 |
| JM-12D | H-κ | 3-23 or 3-23D*01 F | 4*02 F | 2-2*01 F | 3 | CAKAPRVVQDYFDSW | 15 | 12 | 1D-13*01 F | 4*01 F | CQQFNNYLLTF | 6 | 3 |
| JM-11C | H-𝝺 | 4-39*01 F | 6*02 F | 5-18*01 F | 1 | CARVDTAIFYLGYAMDVW | 9 | 4 | 1-44*01 F | 3*02 F | CSAWDDSLNGPVF | 5 | 3 |
| **Donor V108** | | | | | | | | | | | | | |
| JL-4A | H-κ | 3-30*03 or 18 or 3-30-5*01 F | 4*02 F | 6-13*01 F | 2 | CAKTQGPYAAAGLYYFDYW | 14 | 9 | 1-33 or 1D-33*01 F | 4*01 F | CQQFDHLPLTF | 7 | 3 |
| JL-1C | H-κ | 4-39*01 F | 4*01 F | 5-18*01 F | 1 | CARQTIMVNFIDYW | 7 | 4 | 3-15*01 F | 4*01 F | CQQYNNWPPLTF | 1 | 1 |
| JL-8D | H-κ | 3-13*01 F | 2*01 F | 1-26*01 F | 3 | CARDSGSSTWYFDLW | 6 | 5 | 1-39 or 1D-39*01 F | 5*01 F | CQQSYTNPPITF | 6 | 6 |
| JL-1A | H-𝝺 | 1-18*01 F | 3*02 F | 6-13*01 F | 2 | CARVQGRIAAPGWEAFDIW | 4 | 2 | 3-21*03 F | 3*02 F | CQVWDSSSDLHWVF | 5 | 3 |
| JL-12A | H-𝝺 | 3-64*01 F | 1*01 F | 2-2*01 F | 2 | CAKGECSSTSCPSADYFQKW | 11 | 7 | 3-21*03 F | 1*01 F | CQVWDGTYDPPWVF | 5 | 2 |
| JL-7D | H-𝝺 | 4-39*01 F | 4*02 F | 5-12*01 F | 1 | CARLSIDATMHFDYW | 6 | 5 | 2-14*03 F | 2*01 or 3*01 F | CSSYTTSSTLVF | 6 | 5 |
| **Infection** | | | | | | | | | | | | | |
| **Donor P2** | | | | | | | | | | | | | |
| FS-8C | H-𝝺 | 4-59*01 F | 6*03 F | 3-9*01 F | 2 | CARYAGGNYDILTGYYTSNYYMDVW | 0 | 0 | 2-23*02 F | 3*02 F | CCSYAGSSTWVF | 0 | 0 |
| GM-11B | H-𝝺 | 4-39*01 F | 5*02 F | 3-10*01 F | 2 | CARRGNYYGSGSYYWGWFDPW | 0 | 0 | 1-44*01 F | 2*01 or 3*01 F | CAAWDDSLNGVVF | 0 | 0 |
| **Donor P3** | | | | | | | | | | | | | |
| FU-2A | H-𝝺 | 3-30-3*01 F | 5*02 F | - | - | CADMGSGTF (TRP 118 not identified) | 8 | 4 | 7-43*01 F | 3*02 F | CLLYYGGPWVF | 4 | 2 |
| GJ-11E | H-κ | 3-49*04 F | 4*02 F | 2-15*01 F | 2 | CTREGGSGLGFYYFDYW | 11 | 9 | 1-39*01 F or 1D-39*01 F | 1*01 F | CQQSYTTPGWTF | 3 | 1 |
| HC-5E-1 | H-κ | 3-53*01 F | 6*02 F | non | non | CARDLYYYGMDVW | 4 | 3 | 1-9*01 F | 2*01 F | CQQLNSYSYTF | 0 | 0 |
| HC-5E-2 | H-𝝺 | 3-53*01 F | 6*02 F | non | non | CARDLYYYGMDVW | 4 | 3 | 1-44*01 F | 3*02 F | CATWDNSLNNWVF | 13 | 9 |
| **Donor P4** | | | | | | | | | | | | | |
| GR-1C | H-𝝺 | 3-53*01 F | 6*02 F | 7-27*01 F | 2 | CARGELGYYYGMDVW | 0 | 0 | 1-51*01 F | 2*01 or 3*01 F | CGTWDSSLSAGVF | 1 | 0 |
| GR-4C | H-κ | 3-53*01 F | 4*02 F | 3-16*01 F | 3 | CARELRGYFDYW | 3 | 3 | 1-33*01 or 1D-33*01 F | 3*01 F | CQQYDNLPSFTF | 0 | 0 |
| GR-5C | H-κ | 1-69*18 F | 5*02 F | 3-22*01 F | 2 | CARVSGRNYYDSSGYSYNWFDPW | 0 | 0 | 1-39*01 F or 1D-39*01 F | 2*01 F | CQQSYSTVYTF | 1 | 1 |

Abbreviations: H, heavy; κ, kappa; 𝝺, lambda; Vh, variable gene segment of the heavy chain variable domain; Jh, joining gene segment of the heavy chain variable domain; Dh, diversity gene segment of the heavy chain variable domain; nt Mut, number of nucleotide mutations; aa Sub, number of amino acid substitutions; Vl, variable gene segment of the light chain variable domain; Jl, joining gene segment of the light chain variable domain; rf, reading frame.
