# Supplementary material for: The presence of broadly neutralizing anti-SARS-CoV-2 RBD antibodies elicited by primary series and booster dose of COVID-19 vaccine
Source: PLoS Pathog. 2024 Jun 10;20(6):e1012246. doi: 10.1371/journal.ppat.1012246 (PMC11192315; doi:10.1371/journal.ppat.1012246)
Supplement: S5 Table — (DOCX) [file ppat.1012246.s006.docx]

**S5 Table.** **IC_50_ values of neutralization data for Figs 2B and 4F.**

| IC_50_ (μg/mL) | WT | BA1 | BA2 | BA4/5 | BQ1 | XBB.1.5 | XBB.1.16 |
| --- | --- | --- | --- | --- | --- | --- | --- |
| JE-5C | 0.0106 | 0.0033 | 0.0059 | 0.0185 | 0.4263 | 1.6000 | 1.7290 |
| JE-5A | 0.0197 | 0.1474 | 0.1318 | 0.2312 | 14.5500 | 44.0100 | 25.2900 |
| JH-8B | 0.0094 | 0.1422 | 0.0348 | 0.0448 | 0.4325 | 0.2527 | 0.2785 |
| JH-11A | 0.0005 | 0.7557 | 0.0016 | 0.0168 | 0.1426 | 0.0725 | 0.0670 |
| JM-1A | 0.0064 | 0.0304 | 0.0302 | 0.9820 | 0.6383 | 0.1689 | 0.0565 |
| JM-6A | 0.0089 | 0.0219 | 0.0187 | 2.5720 | 0.7544 | 0.8003 | 0.2942 |
| JD-2B | 0.0418 | 0.0623 | 0.1960 | 0.6325 | 6.7460 | 0.6382 | 0.3220 |
| IW-12A | 0.0259 | 0.5911 | 0.6472 | 61.2900 | > 100 | 72.1500 | > 100 |
| JE-2A | 0.3170 | 0.0940 | 0.4924 | 14.9800 | > 100 | 90.1400 | 98.3700 |
| JE-4C | 0.0339 | 0.3332 | 0.0796 | 0.4290 | > 100 | 1.0160 | 0.6822 |
| JH-1B | 1.8780 | 0.4739 | > 100 | > 100 | > 100 | > 100 | > 100 |
| JL-8B | 0.1265 | 3.9510 | 1.8380 | 3.3400 | 34.7300 | 1.3600 | 7.4760 |
| IW-11C | 0.0661 | 2.4420 | 1.5310 | 2.5030 | 7.1670 | 7.4270 | 1.3630 |
| JL-2B | 0.3389 | 0.3651 | 0.3344 | 0.5419 | 0.5903 | 1.2560 | 1.1000 |
| JL-8C | 0.0904 | 0.1245 | 0.1640 | 0.3882 | 0.7248 | 0.5641 | 0.5361 |
| IY-2A | 0.0250 | 0.2094 | 0.2413 | 0.1698 | 0.2918 | 0.7682 | 0.3071 |
| JC-7A | 0.2245 | 4.4350 | 3.3280 | 1.7380 | 3.9700 | 8.4430 | 1.4330 |
| JC-7C | 0.0431 | 1.4110 | 10.3100 | 8.1170 | 7.0850 | 10.3200 | > 100 |
|  |  |  |  |  |  |  |  |
| JM-1A-G32F | 0.0167 | 0.0189 | 0.0299 | 0.0806 | 0.1049 | 0.1362 | 0.0686 |
| JM-1A-N59F | 0.0074 | 0.0180 | 0.0106 | 3.0400 | 1.4600 | 0.4468 | 0.0362 |
