# Supplementary material for: The presence of broadly neutralizing anti-SARS-CoV-2 RBD antibodies elicited by primary series and booster dose of COVID-19 vaccine
Source: PLoS Pathog. 2024 Jun 10;20(6):e1012246. doi: 10.1371/journal.ppat.1012246 (PMC11192315; doi:10.1371/journal.ppat.1012246)
Supplement: S6 Table — (DOCX) [file ppat.1012246.s007.docx]

**S6 Table. Cryo-EM data processing and structural refinement.**

| **Complex Structures**  **(with Spike)** | **JE-5C**  (Delta variant) | **JH-8B**  (BA.1 variant) | **JL-8C**  (Delta variant) | **JM-1A** (Delta variant) |
| --- | --- | --- | --- | --- |
| **Data collection and processing** |  |  |  |  |
| Magnification | 106,000 | 106,000 | 105,000 | 105,000 |
| Voltage (kV) | 300 | 300 | 300 | 300 |
| Electron exposure (e– /Å²) | 54.06 | 54.06 | 52.43 | 50.79 |
| Defocus range (μm) | -1.5 ~ -2.0 | -1.5 ~ -2.0 | -1.5; -1.75; -2 | -1.4 ~ -2.2 |
| Pixel size after binning (Å) | 0.83 | 0.83 | 0.83 | 0.83 |
| Symmetry imposed | C1 | C1 | C3 | C1 |
| Map resolution (Å) | 3.93 | 4.55 | 3.27 | 3.64 |
| FSC threshold | 0.143 | 0.143 | 0.143 | 0.143 |
|  |  |  |  |  |
| **Refinement** |  |  |  |  |
| Initial model used (PDB code) | 7W99 | 8DM9 | 8HHY | 7V7V |
| Model resolution (Å) | 3.9 | 4.5 | 3.4 | 3.1 |
| Map sharpening B factor (Å²) | -150 | -150 | -109.8 | -107.8 |
| Model composition |  |  |  |  |
| Non-hydrogen atoms | 27906 | 24950 | 27822 | 25492 |
| Protein residues | 3583 | 3158 | 3596 | 3270 |
| Water | 0 | 0 | 0 | 0 |
| Ligands | 0 | 17 (NAG)  1 (BMA) | 0 | 0 |
| R.m.s. deviations |  |  |  |  |
| Bond lengths (Å) | 0.004 | 0.004 | 0.006 | 0.015 |
| Bond angles (°) | 1.026 | 1.009 | 0.991 | 1.317 |
| Ramachandran plot |  |  |  |  |
| Favored (%) | 94.04 | 96.37 | 94.31 | 92.98 |
| Allowed (%) | 5.96 | 3.50 | 5.69 | 6.93 |
| Outliers (%) | 0.00 | 0.13 | 0.00 | 0.09 |
| Rotamer outliers (%) | 0.06 | 0.04 | 0.42 | 0.18 |
| MolProbity score | 2.12 | 2.00 | 1.96 | 2.16 |
| C-beta outliers (%) | 0.00 | 0.00 | 0.00 | 0.10 |
| Cis-proline/general | 1.2/0.0 | 0.6/0.0 | 0.0/0.0 | 0.6/0.0 |
