# Supplementary material for: The presence of broadly neutralizing anti-SARS-CoV-2 RBD antibodies elicited by primary series and booster dose of COVID-19 vaccine
Source: PLoS Pathog. 2024 Jun 10;20(6):e1012246. doi: 10.1371/journal.ppat.1012246 (PMC11192315; doi:10.1371/journal.ppat.1012246)
Supplement: S7 Table — (DOCX) [file ppat.1012246.s008.docx]

**S7 Table. X-ray diffraction data processing and structural refinement.**

| Data collection | RBD/JE-5C | RBD/JM-1A |
| --- | --- | --- |
| Wavelength | 1.00 | 1.00 |
| Resolution range | 38.16 – 3.45 (3.73 – 3.45) | 36.94 – 1.90 (1.93 – 1.90) |
| Space group | *I 1 2 1* | *C 1 2 1* |
| Unit cell (a, b, c, α, β, γ) | 109.16, 130.34, 131.775, 90 105.18, 90 | 148.359, 107.03, 87.3401, 90 100.75, 90 |
| Unique reflections | 21549 (4508) | 105363 (5145) |
| Multiplicity | 3.0 (2.9) | 4.8 (4.7) |
| Completeness (%) | 91.8 (93.2) | 100.0 (100.0) |
| Mean I/sigma(I) | 5.6 (2.3) | 5.0 (1.0) |
| R-merge | 0.157 (0.419) | 0.148 (1.312) |
| CC1/2 | 0.923 (0.701) | 0.992 (0.482) |
| Structural refinement |  |  |
| Resolution range | 36.17 – 3.45 (3.57 – 3.45) | 19.91 - 1.94 (2.01 - 1.94) |
| R-work | 0.2407 | 0.1916 |
| R-free | 0.2802 | 0.2129 |
| Number of atoms | 9497 | 10727 |
| RMS (bonds) | 0.003 | 0.010 |
| RMS (angles) | 0.710 | 1.32 |
| Ramachandran favored (%) | 88.40 | 96.01 |
| Ramachandran allowed (%) | 11.27 | 3.82 |
| Ramachandran outliers (%) | 0.33 | 0.16 |
| Rotamer outliers (%) | 7.07 | 2.45 |
| Clashscore | 11.59 | 8.24 |
| Average B-factor | 61 | 37 |
| Wilson B-factor | 62 | 25 |
