# Supplementary figures and images for: The presence of broadly neutralizing anti-SARS-CoV-2 RBD antibodies elicited by primary series and booster dose of COVID-19 vaccine
Source: PLoS Pathog. 2024 Jun 10;20(6):e1012246. doi: 10.1371/journal.ppat.1012246 (PMC11192315; doi:10.1371/journal.ppat.1012246)

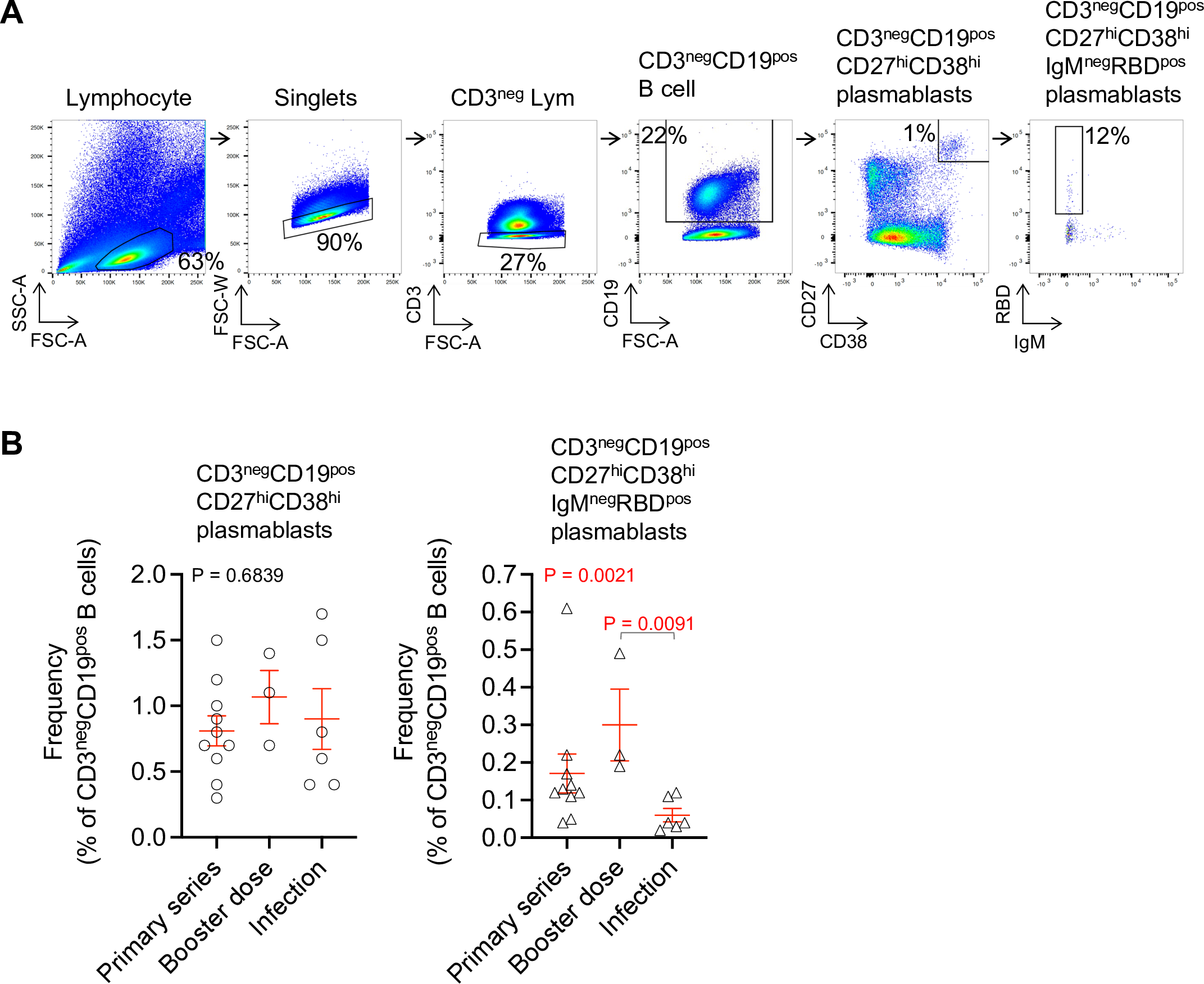

Supplement: S1 Fig — (A) Gating strategy for plasmablasts and IgMnegRBDpos plasmablasts. (B) Frequencies of plasmablasts and IgMnegRBDpos plasmablasts among donors after primary series (n = 10) and booster (n = 3) dose of COVID-19 vaccine and after infection (n = 6). B cell frequencies of two donors (donors 57, 59) after primary series of COVID-19 vaccine were unavailable because their flow cytometry files were misidentified. Each symbol represents a single donor and red line represents the mean and standard error of the mean. Kruskal-Wallis test with post-hoc Dunn’s multiple comparison was applied to analyze the difference in groups. The P value of less than 0.05 was considered significant. (TIF) [file ppat.1012246.s009.tif]

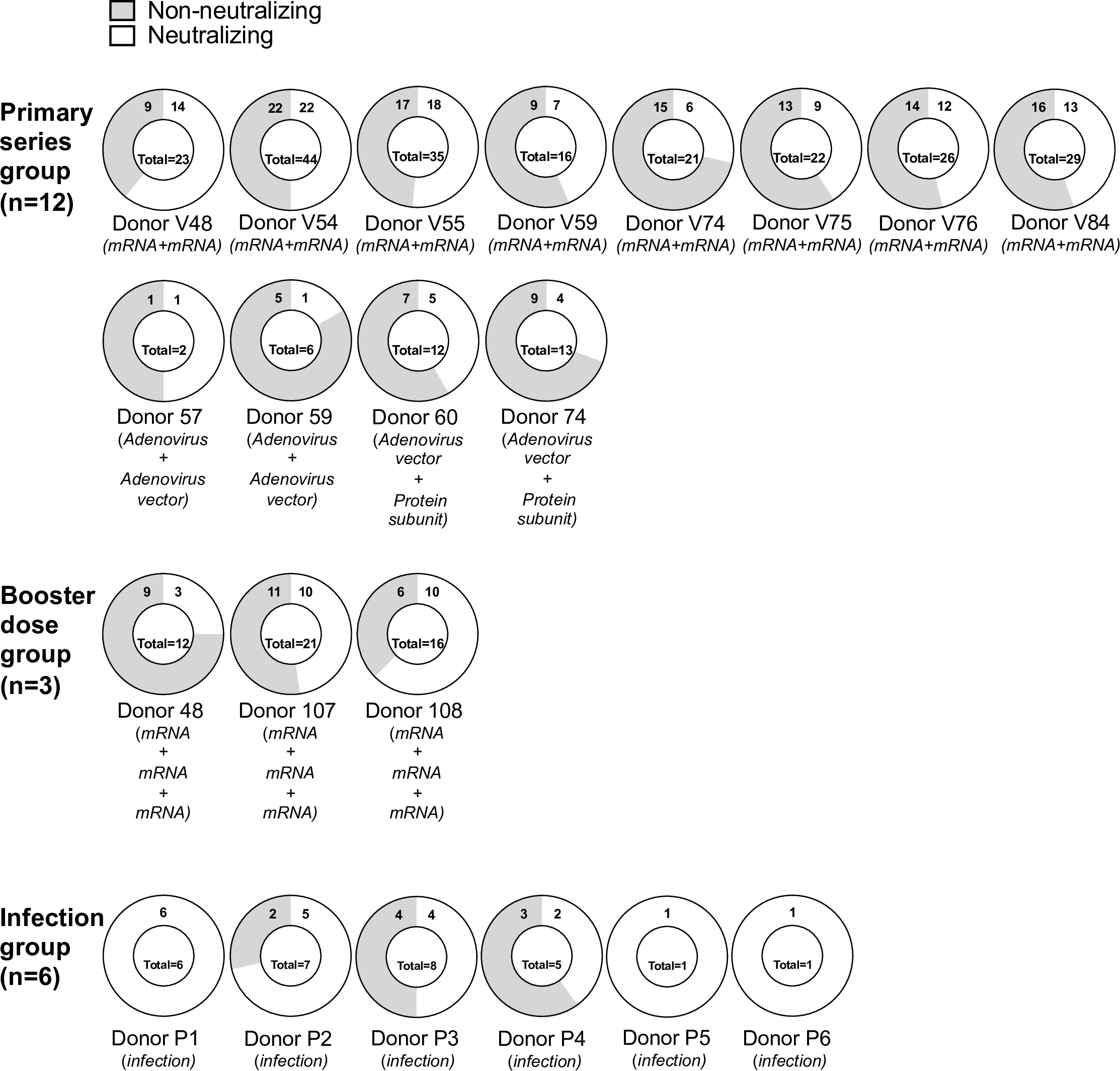

Supplement: S2 Fig — Donors after receiving COVID-19 vaccine primary series and booster dose and COVID-19 patients were enrolled. mRNA, mRNA-1273 vaccine; adenovirus vector, ChAdOx1 vaccine; protein subunit, MVC-COV1901 vaccine. The ChAdOx1 vaccine is an adenoviral vector COVID-19 vaccine that encodes a wild-type spike including the transmembrane domain. The MVC-COV1901 is a protein subunit COVID-19 vaccine based on the stable prefusion spike adjuvanted with CpG1018 and aluminum hydroxide. The mRNA-1273 vaccine is a mRNA-based COVID-19 vaccine that encodes the prefusion stabilized full-length spike. (TIF) [file ppat.1012246.s010.tif]

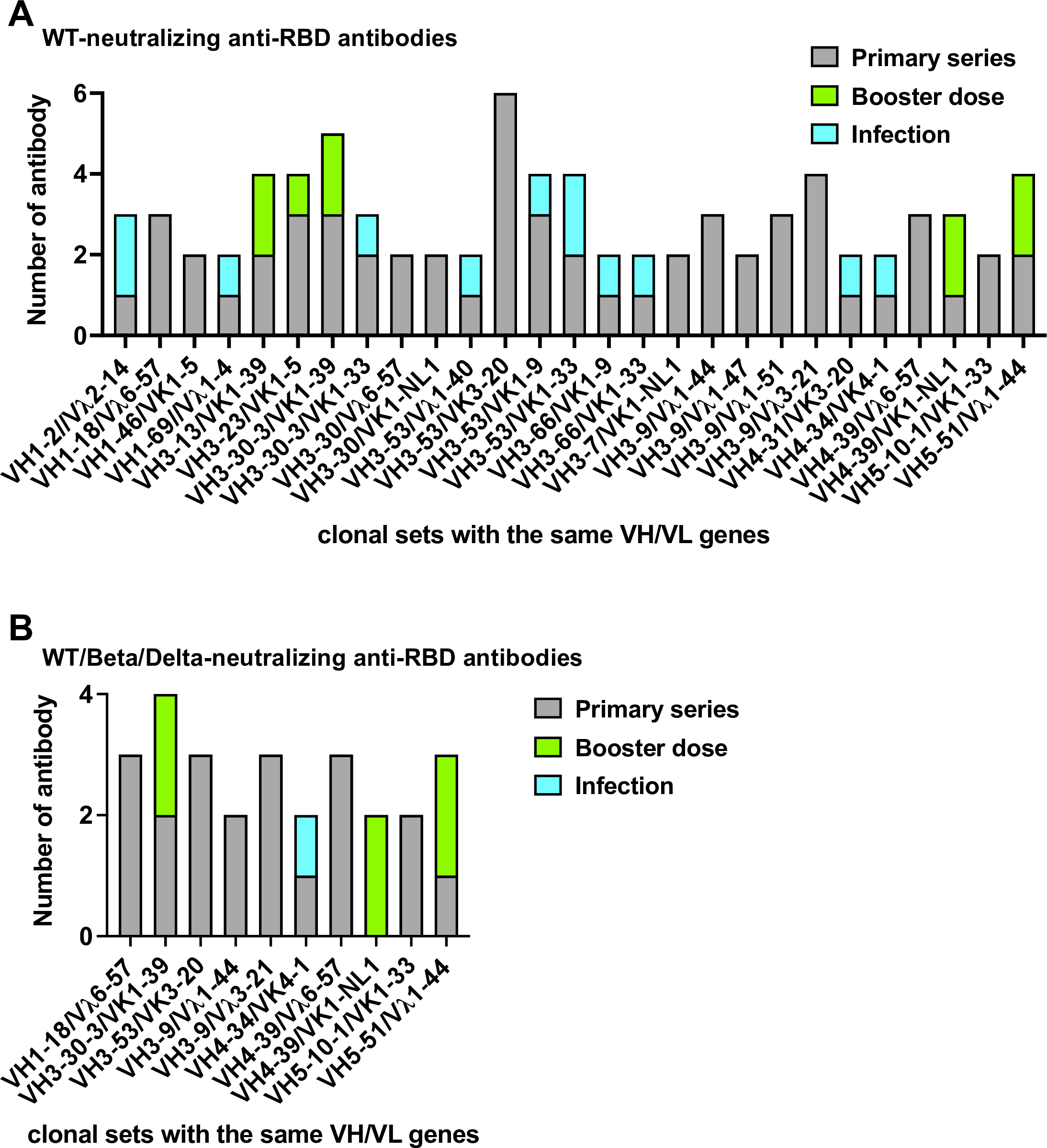

Supplement: S3 Fig — Clonal sets of (A) wild type-neutralizing and (B) cross-neutralizing anti-RBD antibodies. The clonal sets included multiple anti-RBD B cells with the same heavy and light chain variable region genes. WT, wild-type SARS-CoV-2 (Wuhan-1); Beta, Beta variant of SARS-CoV-2; Delta, Delta variant of SARS-CoV-2; VH, heavy chain variable region; VK, kappa chain variable region; Vλ, lambda chain variable region. (TIF) [file ppat.1012246.s011.tif]

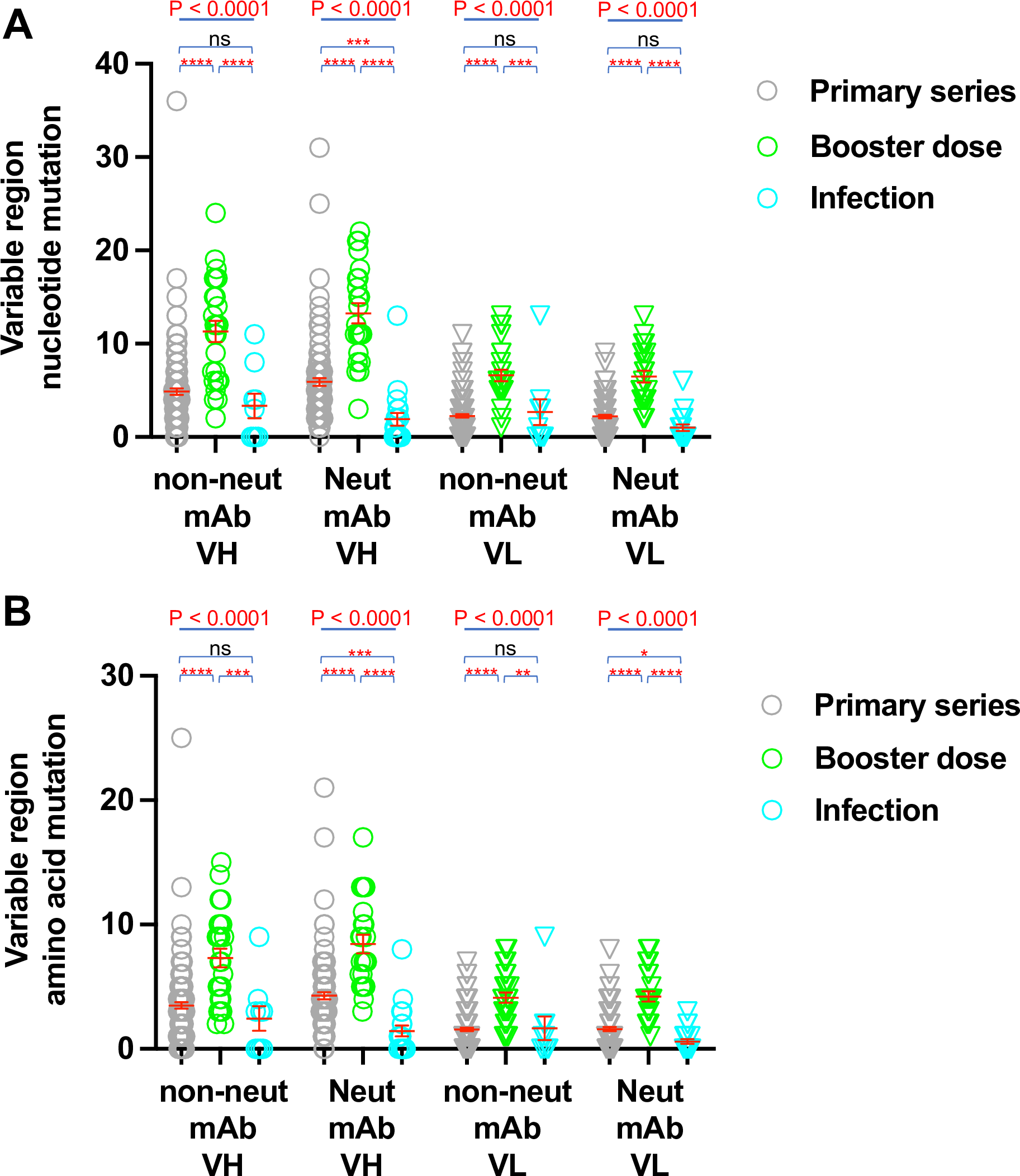

Supplement: S4 Fig — Analysis of variable region and (A) nucleotide and (B) amino acid mutations of anti-RBD antibodies. Each symbol represents an antibody and red line represents the mean and standard error of the mean. Statistical significance among subgroups was analyzed by two-way ANOVA and Tukey’s post hoc test. The results of post hoc comparisons between subgroups were displayed in the graph. *, P < 0.05; **, P < 0.01; ***, P < 0.001; ****, P < 0.0001; ns, not significant. VH, heavy chain variable region; VL, light chain variable region; mAb, monoclonal antibody; neut, neutralizing. (TIF) [file ppat.1012246.s012.tif]

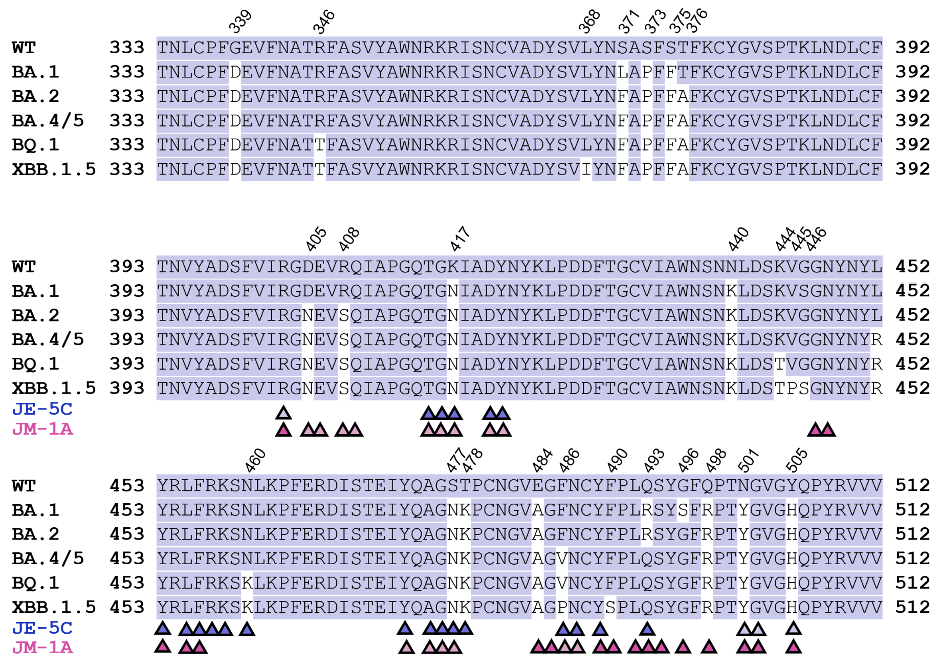

Supplement: S5 Fig — Sequences of RBD (partial, 333–512) of WT and Omicron subvariants are aligned and shaded for identical residues. Key variable residues of RBD are labeled and highlighted in white. RBD epitope residues for JE-5C (purple) and JM-1A (pink) are marked with triangles below the sequence, with dark colors for heavy chain and light colors light chain. Although the antibody-interacting residues overlap with the variable residues of RBD, most of the mutations do not affect their binding affinity or neutralization breadth. (PNG) [file ppat.1012246.s013.png]

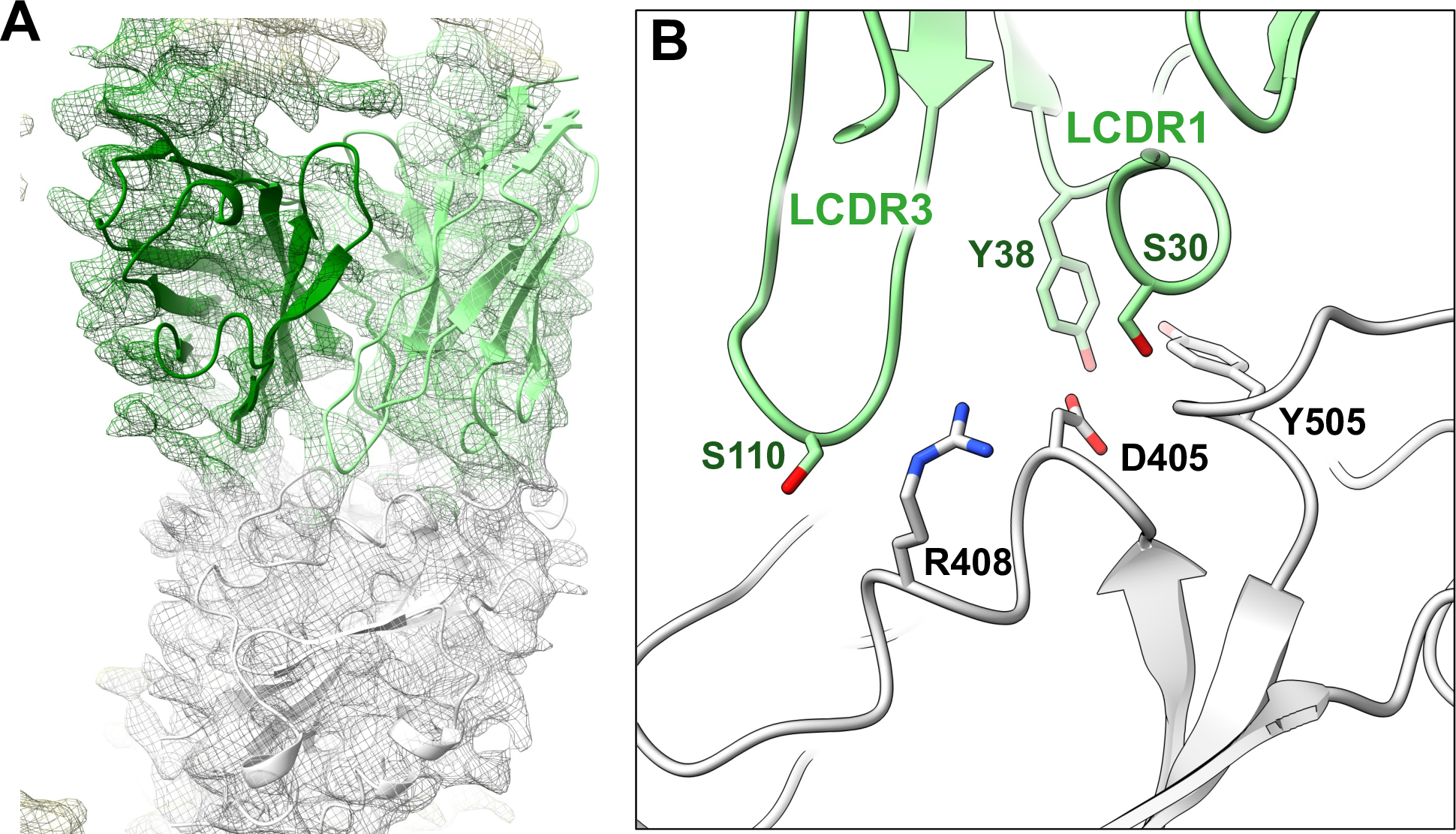

Supplement: S6 Fig — (A) Cryo-EM volume shown in mesh of the Spike/JH-8B structure at resolution 4.5Å in the interface between RBD (light grey) and JH-8B (green). (B) LCDR of JH-8B (green) is in vicinity of RBD (light grey), with potential interacting residues highlighted as sticks and labeled accordingly. HC, heavy chain; LC, light chain; LCDR, light chain complementarity-determining region. (TIF) [file ppat.1012246.s014.tif]

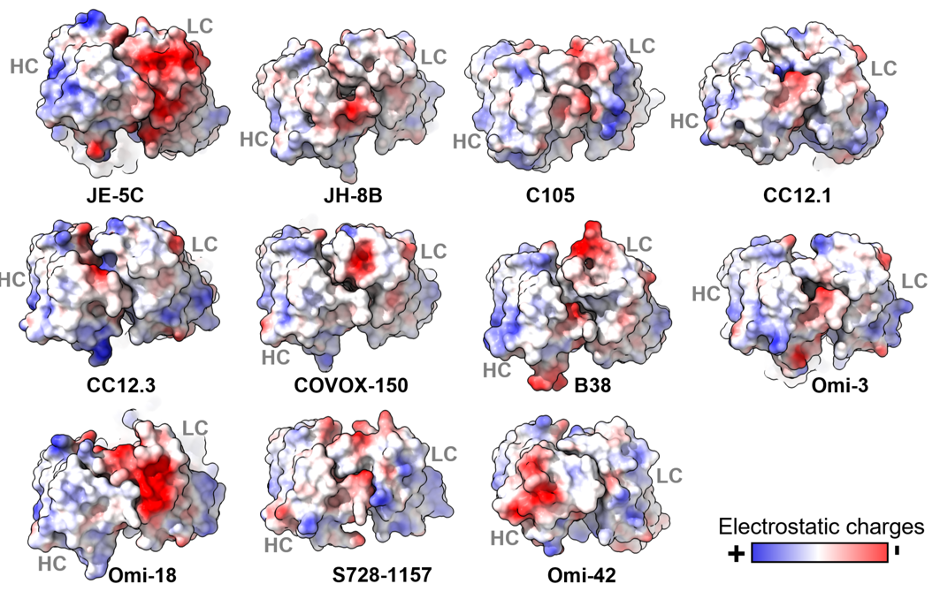

Supplement: S7 Fig — Top views of the 11 VH3-53-family antibody structures are shown with red color indicating negatively charged residues and blue positively charged. HC, heavy chain; LC, light chain. PDB code 6XCM for C105, 8CWV for CC12.1, 6XC4 for CC12.3, 7ZF8 for COVOX-150, 7ZB5 for B38, 7ZF3 for Omi-3, 7ZFB for Omi-18, 8D0Z for S728-1157, and 7ZR7 for Omi42. (PNG) [file ppat.1012246.s015.png]

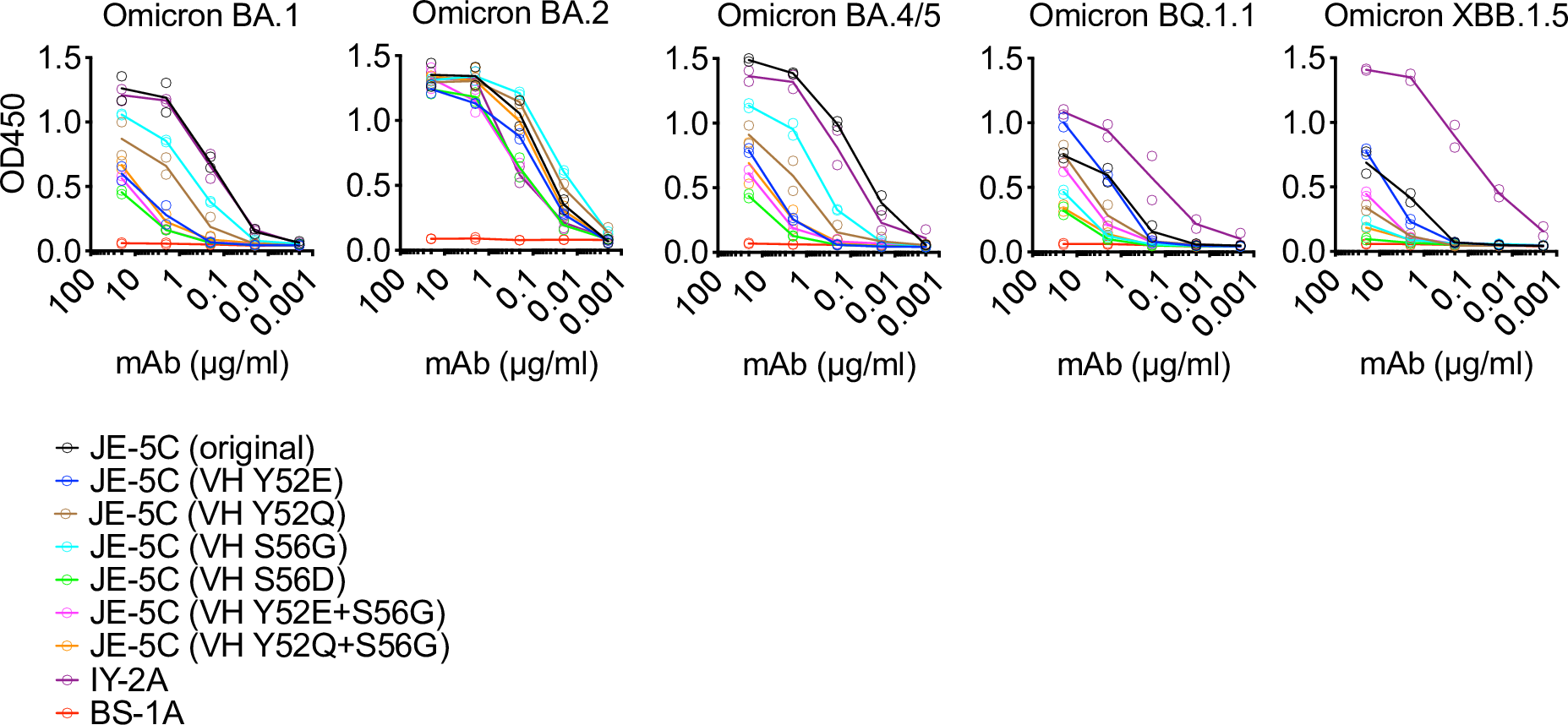

Supplement: S8 Fig — Original JE-5C antibody and site-directed mutants (Y52E, Y52Q, S56G, S56D, Y52E+S56G, Y52Q+S56G of heavy chain variable region) were tested for binding to RBD of various Omicron subvariants. Anti-RBD antibody IY-2A and anti-influenza H3 antibody BS-1A were included as controls. mAb, monoclonal antibody; VH, heavy chain variable region. (TIF) [file ppat.1012246.s016.tif]

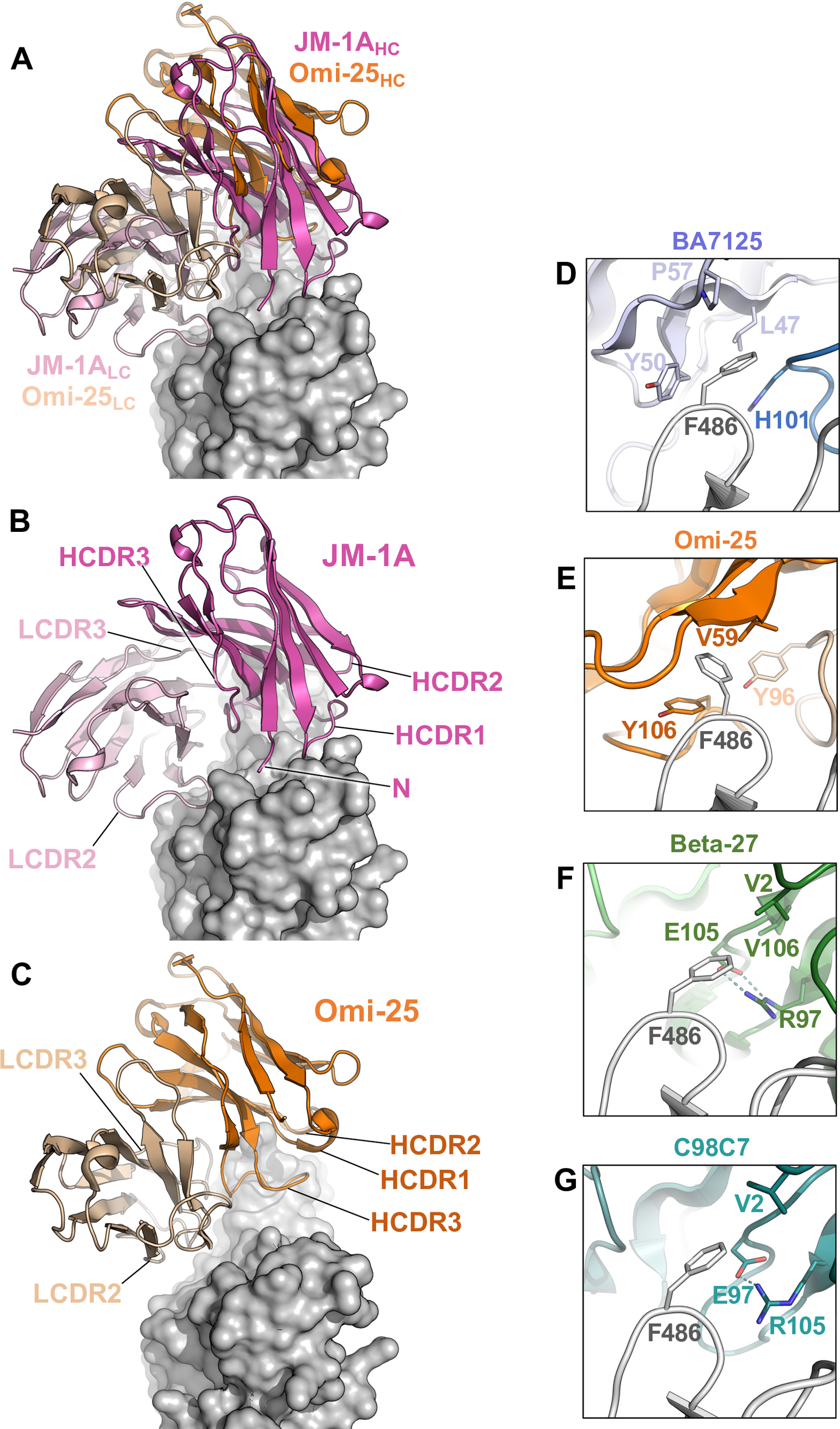

Supplement: S9 Fig — (A-C) JM-1A/RBD structure was superimposed with Omi-25/RBD structure with RBD shown as grey surface and Fabs in ribbon (JM-1A, pink; Omi-25, orange). CDRs and N-terminus are labeled. (D-G) Different local interfaces around the hotspot residue F486 of RBD in structures of BA7125 (D, blue), Omi-25 (E, orange), Beta-27 (F, green) and C98C7 (G, cyan). (TIF) [file ppat.1012246.s017.tif]

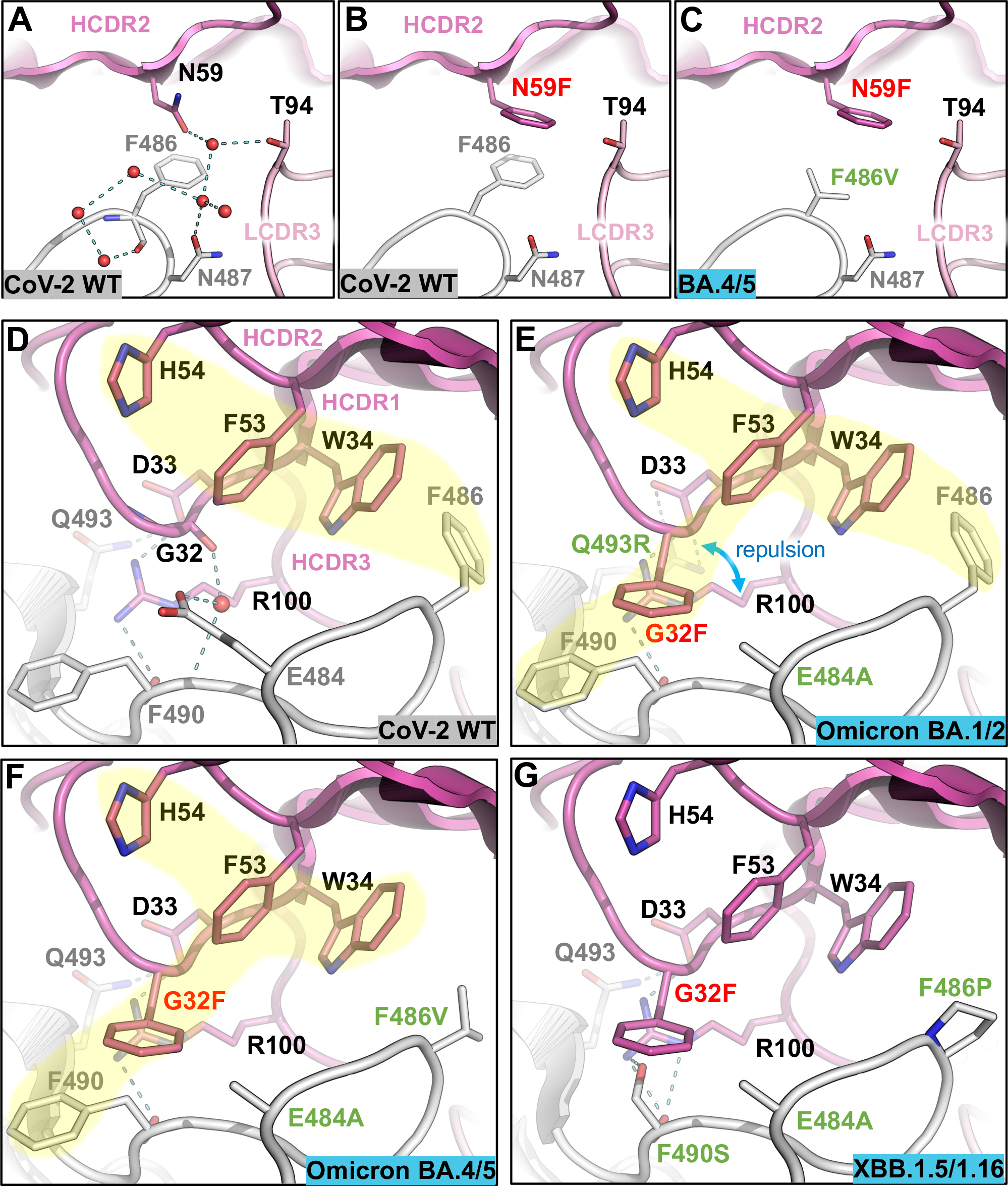

Supplement: S10 Fig — (A-C) JM-1A/RBD complex structure is shown as ribbons with key residues drawn as sticks and water molecules in red spheres. Mutated site (N59F, red) and RBD hotspot residue (F486V, green) are structurally modelled. CDRs, key residues and virus strains are labeled. (D-G) JM-1A/RBD complex structure is shown in the same way, with mutated site (G32F, red) and RBD hotspot residues (green) structurally modelled. Virus strains are labeled at the right bottom corner of each panel. A light-yellow shade highlights the multiple-ring aromatic stacking in (D-F). Hydrogen bonds are drawn as blue dashed lines. (TIF) [file ppat.1012246.s018.tif]

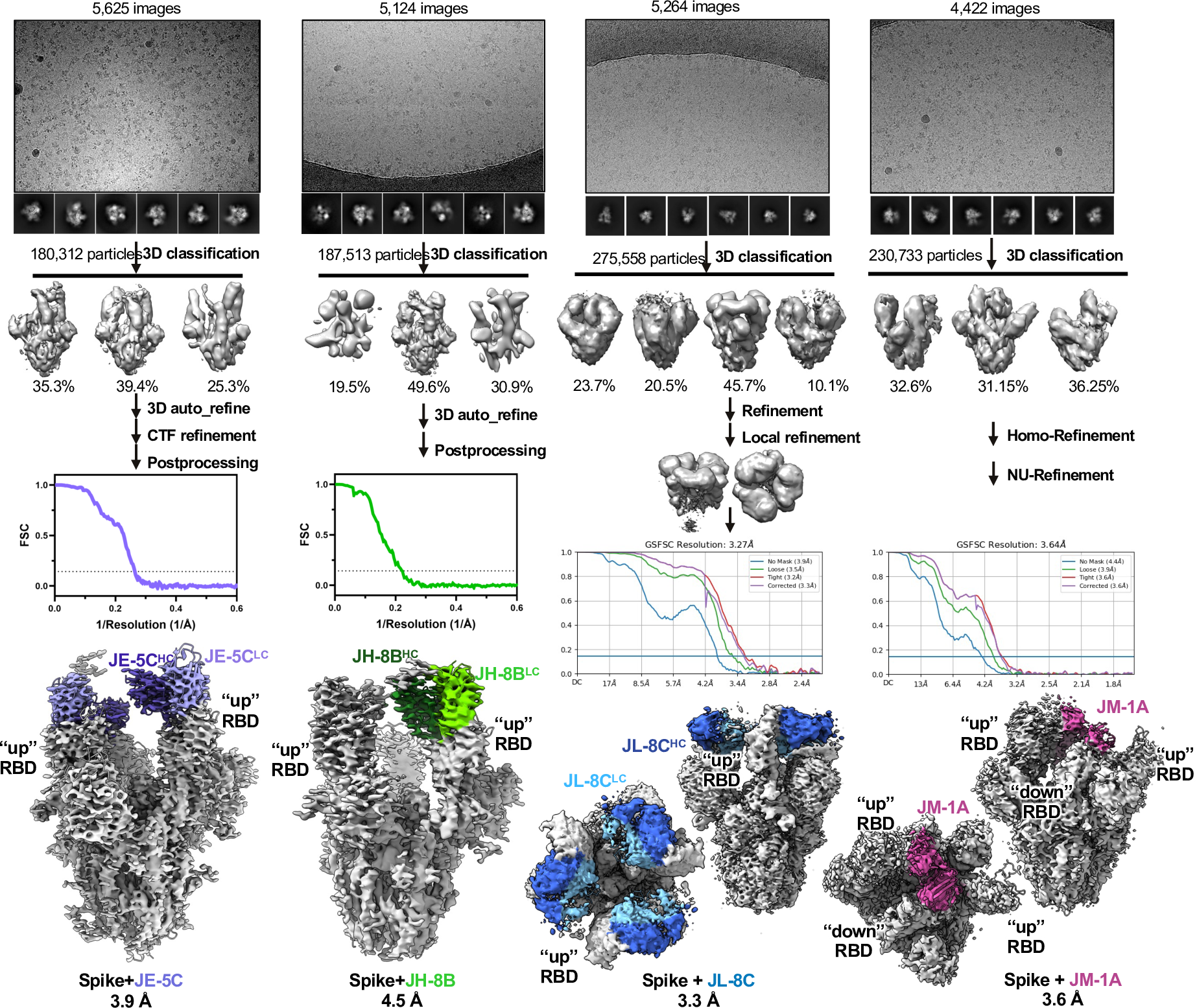

Supplement: S11 Fig — Raw micrographs, representative 2D classes, 3D classes, FSC curves and the fitted final map are shown for JE-5C, JH-8B, JL-8C, and JM-1A with particle number/percentage specified for each step. Cryo-EM volumes are colored white for Spike, purple for JE-5C, green for JH-8B, and blue for JL-8C, with darker colors indicating heavy chain and lighter colors light chain. All the visible RBDs in these structures adopt the up conformation. (TIF) [file ppat.1012246.s019.tif]
